# Correlates of HIV-1 control after combination immunotherapy

**DOI:** 10.1038/s41586-025-09929-5

**Published:** 2025-12-01

**Authors:** M. J. Peluso, D. A. Sandel, A. N. Deitchman, S. J. Kim, T. Dalhuisen, H. P. Tummala, R. Tibúrcio, L. Zemelko, G. M. Borgo, S. S. Singh, K. Schwartz, M. Deswal, M. C. Williams, R. Hoh, M. Shimoda, S. Narpala, L. Serebryannyy, M. Khalili, E. Vendrame, D. SenGupta, L. S. Whitmore, J. Tisoncik-Go, M. Gale, R. A. Koup, J. I. Mullins, B. K. Felber, G. N. Pavlakis, J. D. Reeves, C. J. Petropoulos, D. V. Glidden, M. H. Spitzer, L. Gama, M. Caskey, M. C. Nussenzweig, K. W. Chew, T. J. Henrich, S. A. Yukl, L. B. Cohn, S. G. Deeks, R. L. Rutishauser

**Affiliations:** 1 Department of Medicine, University of California, San Francisco, San Francisco, CA USA; 2 Department of Otolaryngology-Head and Neck Surgery, University of California, San Francisco, San Francisco, CA USA; 3 Department of Microbiology and Immunology, University of California, San Francisco, San Francisco, CA USA; 4 Department of Clinical Pharmacy, University of California, San Francisco, San Francisco, CA USA; 5 Department of Medicine, San Francisco Veterans Affairs Medical Center, San Francisco, CA USA; 6 Vaccine Research Center, National Institute of Allergy and Infectious Diseases, National Institutes of Health, Bethesda, MD USA; 7 Gilead Sciences, Foster City, CA USA; 8 Department of Immunology, Center for Innate Immunity and Immune Disease, School of Medicine, University of Washington, Seattle, WA USA; 9 Department of Microbiology, University of Washington, Seattle, WA USA; 10 Basic Research Laboratory, Center for Cancer Research, National Cancer Institute, Frederick, MD USA; 11 Labcorp-Monogram Biosciences, San Francisco, CA USA; 12 Department of Epidemiology and Biostatistics, University of California, San Francisco, San Francisco, CA USA; 13 Helen Diller Family Comprehensive Cancer Center, University of California, San Francisco, San Francisco, CA USA; 14 Parker Institute for Cancer Immunotherapy, University of California, San Francisco, San Francisco, CA USA; 15 Department of Clinical Investigation, The Rockefeller University, New York, NY USA; 16 Laboratory of Molecular Immunology, The Rockefeller University, New York, NY USA; 17 Howard Hughes Medical Institute, Chevy Chase, MD USA; 18 Department of Medicine, University of California, Los Angeles, Los Angeles, CA USA; 19 Fred Hutchinson Cancer Center, Seattle, WA USA; 20 Present Address: Department of Microbiology and Immunology, and the Institute on Infectious Diseases, University of Minnesota, Minneapolis, MN USA; 21 Present Address: Instituto Butantan, Sao Paulo, Brazil

**Keywords:** HIV infections, Immunological memory, DNA vaccines, Translational immunology

## Abstract

The identification of therapeutic strategies to induce sustained antiretroviral therapy (ART)-free control of HIV infection is a major priority^1^. Combination immunotherapy including HIV vaccination, immune stimulation, latency reversal and passive transfer of broadly neutralizing antibodies (bNAbs) has shown promise in non-human primate models^2–6^, but few studies have translated such approaches into people. Here we performed a single-arm, proof-of-concept study in ten people living with HIV on ART, combining the following three approaches: (1) therapeutic vaccination with an HIV Gag conserved element-targeted DNA + IL-12 prime/modified vaccinia Ankara (MVA) boost regimen followed by (2) administration of two bNAbs (10-1074, VRC07-523LS) and a toll-like receptor 9 agonist (lefitolimod) during ART suppression, followed by (3) repeat bNAb administration at the time of ART interruption (Clinicaltrials.gov: NCT04357821). Seven out of the ten participants exhibited post-intervention control after pausing ART, independent of residual bNAb plasma levels. Robust expansion of activated CD8^+^ T cells early in response to rebounding virus correlated with a lower median viral load after peak viraemia off ART. These data suggest that combination immunotherapy approaches might prove effective in inducing sustained control of HIV by slowing rebound and improving CD8^+^ T cell responses, and that these approaches should continue to be optimized.

## Abstract (web summary)

Combination immunotherapy approaches might be effective in inducing sustained control of HIV by slowing rebound and improving CD8^+^ T cell responses.

## Main

Identifying an intervention that can induce eradication (a cure) or sustained ART-free control (a remission) of HIV infection is a major public health priority^1^. To date, no single approach has demonstrated a consistent effect in either outcome^7^.

Modest effects on post-ART HIV rebound kinetics have been observed in some studies of T cell-based therapeutic vaccines^8,9^. Administration of bNAbs has been associated with HIV control in some people living with HIV after pausing ART (even after bNAbs have waned)^10–13^, which may be mediated at least in part by the formation of antigen–antibody immune complexes that activate endogenous HIV-specific cytotoxic CD8^+^ T and/or B cells (termed the vaccinal effect)^2,14–17^. Although the definition of HIV control in these studies varied, it generally occurred in 10–35% of participants^7,18^. Stricter levels of control (for example, sustained viral loads <1,000 copies per ml) were less common.

To date, immunotherapy studies in people living with HIV have generally included only one or two interventions. In this proof-of-concept study, we sought to determine whether a more complex combination strategy designed to target multiple pathways induces post-ART control in people living with HIV. Modelled after strategies that demonstrated positive effects in non-human primate (NHP) models of simian immunodeficiency virus (SIV) or simian–human immunodeficiency virus (SHIV) infection^3–6^, we conducted a three-stage study in ten people living with HIV on suppressive ART involving (1) therapeutic vaccination with an HIV Gag conserved element (CE)-targeted DNA/MVA regimen^19^ to enhance HIV-specific T cell responses; followed by (2) a combination of two relatively long-acting bNAbs (10-1074 (ref. ^10^) and VRC07-523LS^20^) and a potential latency reversal agent (lefitolimod^21^, a TLR9 agonist) during ART suppression to reduce the size of the HIV reservoir; and finally (3) administration of the bNAbs immediately before pausing ART and undergoing analytic treatment interruption (ATI) to potentiate host immune responses by slowing rebound and/or possibly inducing the vaccinal effect.

## Study participants

**Fig. 1:**
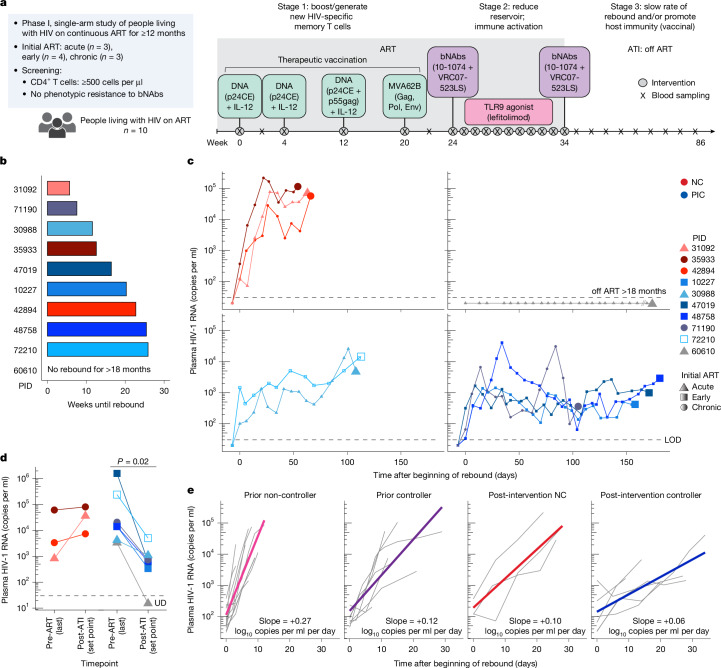
Combination immunotherapy promotes post-intervention control of HIV. **a**, Study schema: administration of combination immunotherapy including a DNA vaccine targeting CEs of HIV Gag, two bNAbs with a TLR9 agonist in ten people living with HIV on ART, followed by a second infusion of bNAbs immediately before an ATI.** b**, The time to HIV rebound after ART discontinuation. **c**, Plasma viral load rebound kinetics from the first day of rebound (*x* = 0); the numbers on the graph indicate set point (the median viral load from 2 weeks after peak viral load to the time of ART restart); the colours indicate the rebound phenotype (red, non-control/typical rebound; blue, viraemic post-intervention control; grey, no rebound for over 18 months off ART). The shape indicates the timing of ART after HIV acquisition: circle, chronic infection; triangle, acute infection (<1 month); square, early infection (1–6 months). End points indicate the day of ART restart, except for the participant with no rebound (grey). The empty square represents a participant with an HLA-B*57 allele. **d**, Comparison of the last available viral load before ART initiation (single value) versus the post-ART set point. Statistical analysis was performed using two-sided Wilcoxon signed-rank tests. **e**, HIV rebound slope (log_10_-transformed copies per ml per day) from the time of rebound to the time of peak viral load after pausing ART in people who were known controllers before starting ART (prior controller, *n* = 7) or not (prior non-controller, *n* = 13) in the absence of any immunotherapeutic intervention, versus non-controllers (*n* = 3) and viraemic post-intervention controllers (*n* = 6) from this trial. LOD, limit of detection; NC, non-control; PIC, post-intervention control; UD, undetectable. Source data

**Extended Data Fig. 1:**
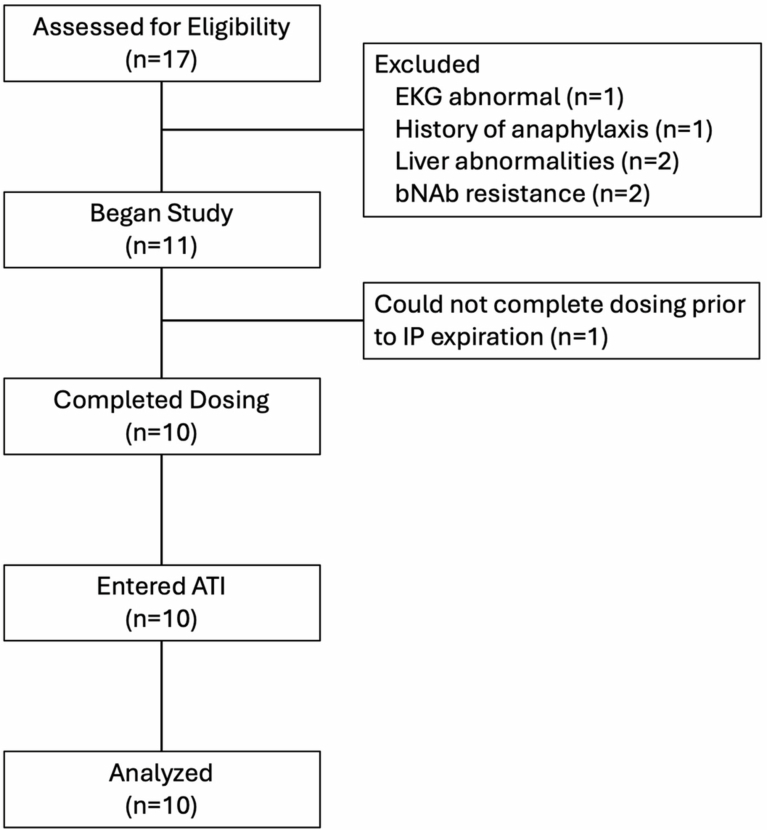
CONSORT diagram.

The final study (Fig. 1a) included ten individuals who had initiated ART during the acute, early or chronic phase of HIV infection (defined as <30 days, between 1 and 6 months, or ≥6 months after the estimated date of acquisition; *n* = 3, 4 and 3 participants, respectively; Methods; the CONSORT diagram is provided in Extended Data Fig. 1, a summary of the study population is provided in Extended Data Table 1 and a redacted study protocol is shown in Supplementary Note 1). The participants were required to have been consistently suppressed on ART for over 12 months, to have a screening CD4^+^ T cell count ≥500 cells per μl and to lack exclusionary comorbidities. We excluded participants whose reservoir provirus was determined to have significantly reduced phenotypic susceptibility to one or both antibodies; we did not exclude individuals whose susceptibility could not be defined (assay failures, attributed to low reservoir sizes in some participants).

## Safety of combination immunotherapy

Safety events are described in Supplementary Table 1. Notably, two participants experienced asymptomatic elevations in liver enzymes (one grade 3 event, one grade 4 event) that resolved over 2–3 months without long-term sequelae. Non-study-related causes of these laboratory abnormalities were considered to be most likely, although a relationship to study interventions could not be excluded. These abnormalities resulted in a temporary FDA hold, which resulted in the participants receiving a variable number of lefitolimod doses (median, 8; range, 1–9), as well as variability in the time between the first and second bNAb doses (range, 10–26.4 weeks); one participant did not receive the second infusion of VRC07-523LS owing to safety concerns. Participant-level clinical information is provided in Supplementary Table 2.

## Effect on HIV rebound kinetics

The median length of the ATI was 36.7 weeks (range, 14.7–77.1). HIV rebound, defined as the first of two consecutive quantifiable (>30 copies per ml) plasma HIV-1 RNA levels, occurred at a median of 16.4 weeks (range, 5.7–25.9) after ART interruption (Fig. 1b). Before rebound, three participants experienced 2–4 weeks during which plasma HIV RNA was intermittently detectable but not quantifiable using standard assays (full viral load data are provided in Supplementary Table 3).

Seven of the participants exhibited evidence of post-ART control. One participant met a prespecified criterion for achieving post-treatment control (based on the CHAMP cohort definition^22^) and was aviraemic for over 18 months off ART (Fig. 1c (grey line)). Six participants experienced rebound and then maintained a low viral load set point for several months (defined as the median of the viral loads measured starting 2 weeks after the peak viral load until ART restart), consistent with post-intervention control^7^, as follows: two of the low viraemic controllers exhibited a slow early rebound pattern (rebound slope, 33–58 copies per ml per day; calculated from time of rebound until the highest (peak) viral load within the first 6 weeks after rebound) and initially maintained viral loads near 1,000–2,000 copies per ml for 2–3 months, followed by a slow increase in viraemia (Fig. 1c (light blue lines)). Four exhibited oscillatory viral loads (one with a higher peak followed by control) with set points below 1,000 copies per ml (calculated over 3–6 months off ART; Fig. 1c (darker blue lines)). The remaining three participants (non-controllers; Fig. 1c (red lines)) exhibited a more typical^18,23^ rapid increase in HIV RNA levels (rebound slope, 823–9,462 copies per ml per day), with two having higher viral load set points (36,133 and 81,245 copies per ml) and one having an intermediate set point (7,415 copies per ml). All seven controllers resumed ART before meeting prespecified ART restart criteria, often due to COVID-related considerations (acute infection, need for a vaccine). Compared with the last available historical plasma viral loads documented before initiating ART, post-ART viral load set points were similar or higher in the three non-controllers but were significantly lower among the seven controllers (median viral load before ART of 14,477 copies per ml versus set point after pausing ART of 651 copies per ml; *P* = 0.02; Fig. 1d). Of the six viraemic post-intervention controllers, one had initiated ART during chronic infection (participant ID (PID) 71190) and one carried an HLA-B*57:01 allele (PID 72210). Five out of the seven post-intervention controllers (one aviraemic plus four viraemic) and one of the three non-controllers carried an HLA-B*35 allele (Supplementary Table 2). Plasma antiretroviral drug levels were confirmed to be undetectable in all participants throughout the ATI (Methods).

## Controllers have lower HIV rebound slope

To contextualize the HIV rebound kinetics observed in the participants from this immunotherapy trial, we compared them to rebound kinetics of people living with HIV on ART enrolled in a separate non-interventional ATI study conducted concurrently at the same site (NCT04359186). That study included twenty individuals, most of whom had initiated ART during chronic infection (Extended Data Table 1); 7 were known to have been spontaneous HIV controllers before initiating ART and 13 had not exhibited HIV control before ART initiation. Compared with the known prior non-controllers from the observational study, the slope of the virologic rebound was significantly lower for the known prior controllers (estimated population average +0.27 versus +0.12 log_10_-transformed copies per ml per day, respectively; *P* < 0.0001). Notably, the slope of the rebound was even lower for both the three non-controllers and the six viraemic post-intervention controllers from the immunotherapy trial (+0.10 and +0.06 log_10_-transformed copies per ml per day, respectively; *P* < 0.0001 comparing each of the two immunotherapy trial groups to the prior non-controllers; *P* = 0.0004 comparing between the two controller groups; Fig. 1e and Supplementary Table 4).

## bNAb exposure does not predict set point

**Fig. 2:**
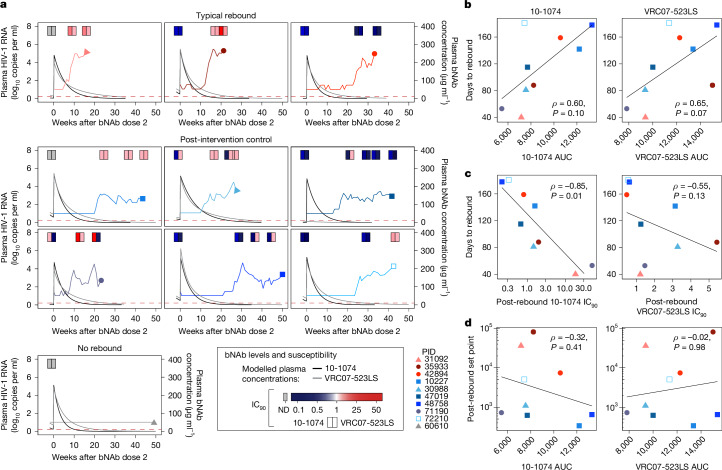
bNAb PK and susceptibility predict time to HIV rebound but not post-ART set point. **a**, Viral load and modelled bNAb concentrations over time after the second dose of bNAbs in each participant; bNAb line colours: black, 10-1074; grey, VRC07-523LS. At the top of each graph, phenotypic susceptibility of autologous HIV to neutralization by bNAbs (IC_90_) is depicted in pairs of shaded rectangles (left rectangle, 10-1074; right rectangle, VRC07-523LS). Susceptibility was measured from both cell-associated provirus at baseline (depicted in the left-most rectangles in each graph; grey indicates that the value could not be determined (ND)) as well as from post-rebound plasma virus at multiple timepoints, as indicated. **b**, The correlation between 10-1074 and VRC07-523LS exposure (area under the bNAb concentration–time curve (AUC) after the second dose of bNAbs) and time to rebound. **c**, The correlation between bNAb susceptibility (IC_90_) after rebound and the time to rebound after ATI. **d**, The correlation between bNAb exposure and the post-rebound viral load set point. Statistical analysis was performed using two-sided Spearman’s correlation (**b**–**d**). Source data

**Extended Data Fig. 2:**
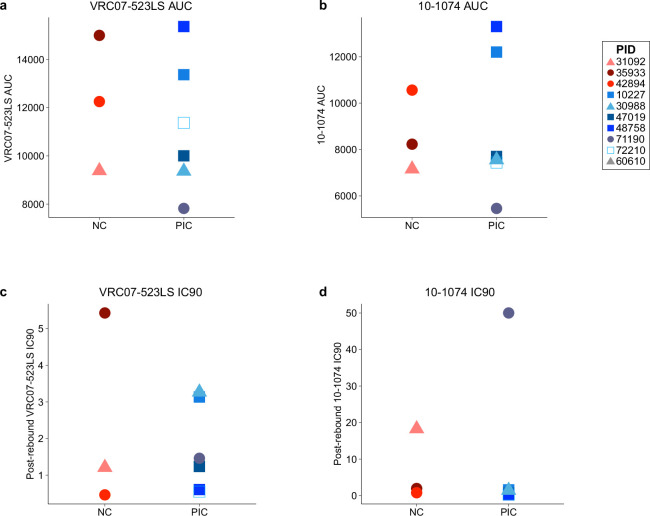
bNAb exposure and susceptibility do not relate to post-intervention control outcomes. Comparison between non-controllers (NC) and post-intervention controllers (PIC) in 10-1074 and VRC07-523LS **a, b**) exposure (AUC, area under the bNAb concentration-time curve following the second dose of bNAbs), and **c, d**) phenotypic susceptibility of autologous HIV to neutralization by bNAbs (inhibitory concentration [IC_90_]). Source data

We next sought to understand whether the altered rebound kinetics were related to variation in lefitolimod exposure and bNAb pharmacokinetics (PK). The number of lefitolimod doses was not associated with variation in viral load set point (*ρ* = −0.077, *P* = 0.85). Although phenotypic susceptibility of proviral sequences to the bNAbs at baseline could not be evaluated for three participants, missing this information did not obviously relate to outcomes (one was a non-controller, one a viraemic controller and the third was the aviraemic controller). The bNAb levels at rebound were variable (0.39–57.6 μg ml^−1^ for 10-1074, and 0.39–73.4 μg ml^−1^ for VRC07-523LS; Fig. 2a). As expected, higher bNAb exposure (area under the curve (AUC) after the last bNAb dose) trended towards being associated with later rebound (10-1074: *ρ* = 0.60, *P* = 0.10; VRC07-523LS: *ρ* = 0.65, *P* = 0.07; Fig. 2b). Higher phenotypic susceptibility of rebound virus to bNAbs (that is, lower 90% inhibitory concentration (IC_90_) at the first sample tested after rebound) was associated with a later time to rebound for 10-1074 but not for VRC07-523LS (10-1074: *ρ* = −0.85, *P* = 0.01; VRC07-523LS: *ρ* = −0.55, *P* = 0.13; Fig. 2c). By contrast, both bNAb exposure and susceptibility were not linked to differences in post-ART viral load set points (10-1074 AUC: *ρ* = −0.32, *P* = 0.41; VRC07-523LS AUC: *ρ* = −0.02, *P* = 0.98; 10-1074 IC_90_: *ρ* = 0.30, *P* = 0.44; VRC07-523LS IC_90_: *ρ* = −0.05, *P* = 0.91; Fig. 2d and Extended Data Fig. 2) or HIV rebound slopes (10-1074 AUC: *ρ* = 0.10, *P* = 0.81; VRC07-523LS AUC: *ρ* = 0.27, *P* = 0.49; 10-1074 IC_90_: *ρ* = 0.32, *P* = 0.41; VRC07-523LS IC_90_: *ρ* = 0.08, *P* = 0.84). Indeed, low set points were maintained over several months even as bNAb levels dropped (and/or the virus lost susceptibility to the bNAbs; Fig. 2a). Finally, anti-drug antibodies were detected transiently in two participants, although these antibodies had no obvious effect on bNAb levels (Supplementary Methods). Thus, increased bNAb susceptibility and exposure was associated with delayed viral rebound but could not directly account for the altered post-ART HIV rebound patterns and lower viral load slope and set points.

## Minimal effect on the HIV reservoir

**Fig. 3:**
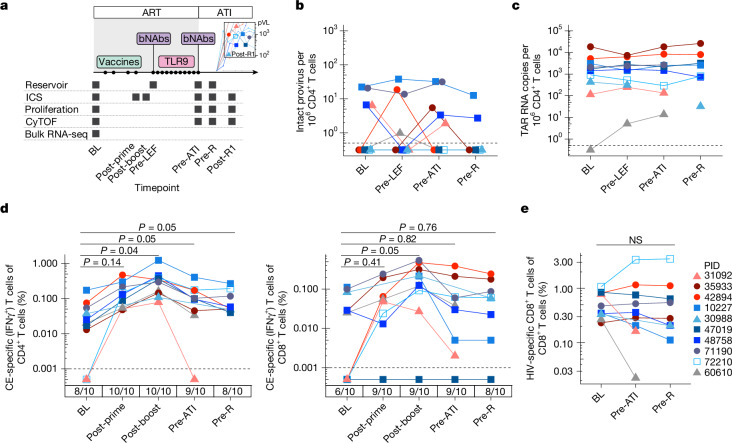
The impact of combination immunotherapy on the HIV reservoir and HIV-specific T cell responses. **a**, Longitudinal peripheral blood sampling timepoints (applies to Figs. 3–5). pVL, plasma viral load. **b**,**c**, CD4^+^ T cell-associated potentially intact HIV DNA, as measured by IPDA (**b**) and HIV RNA (transactivating response (TAR) region, indicating total initiated HIV transcripts) (**c**). **d**, The magnitude of IFNγ^+^ CE-specific CD4^+^ and CD8^+^ T cells as measured by ICS; the numbers below the *x* axis indicate the proportion of participants with detectable CE-specific responses at each timepoint. **e**, The magnitude of total (Gag + Pol + Nef + Env) HIV-specific CD8^+^ T cell responses by ICS. Statistical analysis was performed using linear mixed-effect analysis with Tukey’s multiple-comparison test. Timepoints (applies to all Figs): BL, baseline before interventions (that is, on ART); post-prime, the day of MVA vaccination (>8 weeks after last DNA vaccination); post-boost, 2 weeks after MVA vaccination; pre-LEF, immediately before lefitolimod dosing; pre-ATI, immediately before ATI; pre-R, the last PBMC sampling timepoint available before rebound (sampled within 1–4 weeks before HIV rebound); post-R1, the first PBMC sampling timepoint after rebound (for participants with a sample available ≤28 days after rebound, at a low viral load; all <3,000 copies per ml). NS, not significant. Source data

**Extended Data Fig. 3:**
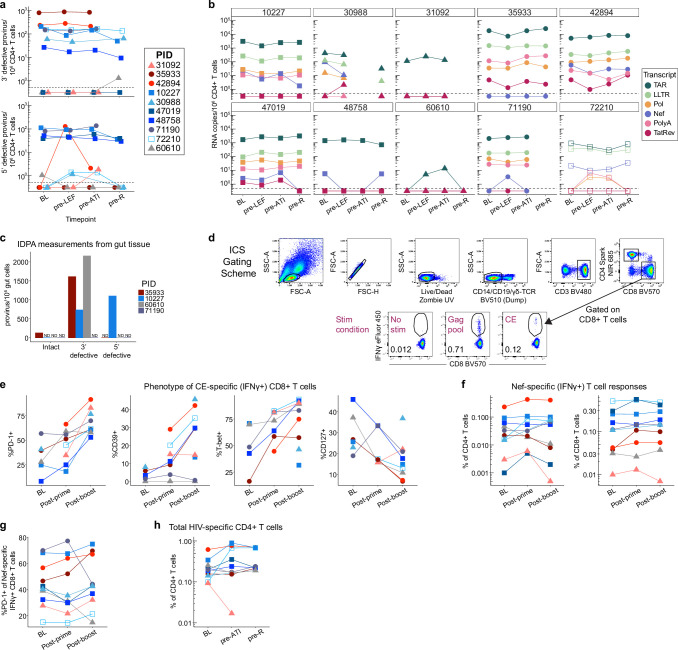
Longitudinal HIV reservoir measurements and HIV-specific T cell responses. **a**) Cell-associated 3′ and 5′ defective HIV DNA in peripheral blood CD4^+^ T cells by IPDA. b) Longitudinal cell-associated HIV RNA transcript measurements. **c**) IPDA measurements from gut tissue in select participants (ND, not detected). All sampled post-ATI. All participants were aviremic at the time of sampling. On ART: 35933, 10227, 71190. Off ART: 60610. **d**) Intracellular cytokine staining (ICS) gating scheme. **e**) Longitudinal phenotypes of HIV Gag/CE-specific (IFNγ^+^) CD8^+^ T cells. Missing points reflect timepoints with no or too few (<50) CE-specific CD8^+^ T cells to phenotype. **f**) Frequency of Nef-specific (IFNγ^+^) CD4^+^ and CD8^+^ T cells. g) PD-1 expression on Nef-specific CD8^+^ T cells. **h**) Frequency of total (IFNγ^+^) HIV-specific CD4^+^ T cells by ICS (sum: Gag+Pol+Nef+Env). IPDA, intact proviral DNA assay. Timepoints (applies to all figures): BL, baseline prior to interventions (i.e., on ART); Post-prime, day of MVA vaccination (>6 weeks after last DNA vaccination); Post-boost, 2 weeks after MVA vaccination; pre-LEF, prior to lefitolimod dosing; pre-ATI, prior to ATI; pre-R, last PBMC sampling timepoint available prior to rebound (sampled within 1-4 weeks prior to HIV rebound). Note: for PID 60610 (aviremic controller), the data shown at the “pre-R” timepoint was evaluated 44 weeks into the ATI. IFNγ, interferon gamma. ICS, intracellular cytokine staining. Source data

The HIV-1 DNA reservoir was estimated using digital droplet PCR (intact proviral DNA assay, IPDA)^24^. In general, intact HIV DNA levels were low before and after the interventions, mostly varying between 0 and 10 copies per million CD4^+^ T cells (sampling timepoints are shown in Fig. 3a,b, Extended Data Fig. 3a and Supplementary Table 5). Three viraemic post-intervention controllers (PIDs 30988, 47019 and 72210) had no measurable intact HIV DNA at any timepoint. No consistent changes in intact or defective HIV DNA were observed during any of the study stages, although the ability to detect such changes was limited by low levels at baseline. While all of the participants had some CD4^+^ T cell-associated HIV RNA transcripts detectable at some timepoint(s), these levels were low at baseline and did not change over the course of the trial (Fig. 3c and Extended Data Fig. 3b). There was no obvious relationship between pre-ATI HIV DNA or RNA levels and post-ART viral load set points (Supplementary Table 6). However, the aviraemic post-intervention controller (PID 60610) had a unique clinical history and virologic profile. This participant initiated pre-exposure prophylaxis without knowing that he had recently acquired HIV; his viral load was 3,343 copies per ml 9 days after starting pre-exposure prophylaxis and 3 days before starting a full antiretroviral regimen. Replication-competent HIV DNA was not detected in a leukapheresis sample collected 3 years after HIV acquisition^25^. In this trial, the participant had among the lowest levels of cell-associated HIV DNA and RNA in both blood and gut (Fig. 3b,c and Extended Data Fig. 3c).

## Effect on HIV-specific T cells

We next explored the relationship between intervention-elicited HIV-specific T cell responses and the control. Therapeutic vaccination elicited new or boosted pre-existing interferon gamma (IFNγ)^+^ Gag/CE-specific CD4^+^ and CD8^+^ T cell responses in all ten participants, as measured using intracellular cytokine staining (ICS; median magnitude at baseline before the vaccination versus at the peak of the response, 2 weeks after the MVA boost: CD4 0.03% versus 0.341% (*P*_adj_ = 0.03); CD8 0.026% versus 0.158% (*P*_adj_ = 0.03); Fig. 3d; the gating scheme is shown in Extended Data Fig. 3d). Gag/CE-specific CD8^+^ T cells upregulated activation markers (PD-1, CD39) and acquired a more effector-differentiated phenotype in response to the vaccine regimen (Extended Data Fig. 3e). The magnitude of both Gag/CE-specific CD4^+^ and CD8^+^ T cell responses contracted back to near-baseline levels by the time of the ATI, which occurred a median of 195 days after the MVA boost (range, 100–268 days). The T cell-boosting effect was specific to vaccine (Gag/CE) antigens (Extended Data Fig. 3f,g). Neither Gag/CE-specific nor total (summed Gag + Pol + Env + Nef) HIV-specific CD4^+^ or CD8^+^ T cell responses were significantly boosted during the period of bNAb-mediated viral suppression after ART was stopped (that is, between the pre-ATI and pre-rebound timepoints; Fig. 3e and Extended Data Fig. 3h). Furthermore, there was no correlation between pre-ATI HIV-specific T cell magnitude and post-ART viral load set point (Supplementary Table 6).

## Peri-rebound immune activation

**Extended Data Fig. 4:**
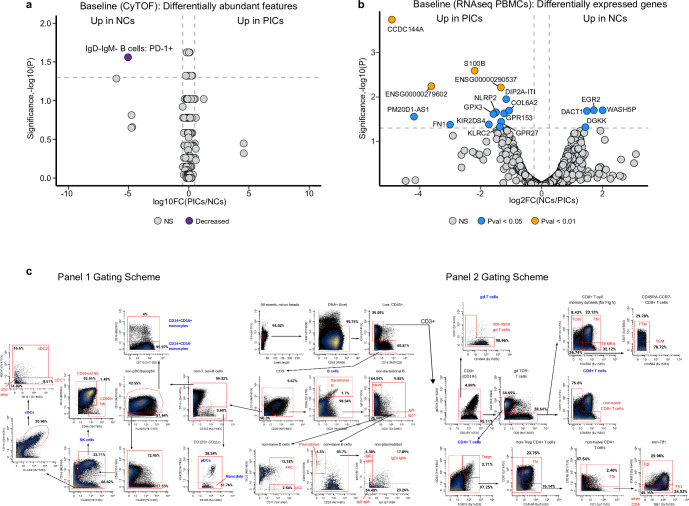
Minimal differences in baseline immune features between post-intervention controllers and non-controllers. **a**) Differentially abundant CyTOF features in the peripheral blood (by two-sided Wilcoxon rank-sum test with Benjamini-Hochberg adjustment for multiple comparisons), and **b**) differentially expressed genes in total peripheral blood mononuclear cells (PBMCs) between the viremic post-intervention controllers (n = 6) and non-controllers (n = 3) at baseline (on ART, prior to interventions). Differential expression analysis was performed using lmfit through limma using the empirical Bayes method to calculate a t-statistic. Significance cut-offs were set at a fold change > 1.5 and nominal *P* value < 0.05. **c**) CyTOF landmark and sub-landmark gating scheme. Populations labelled in blue represent landmark populations and populations labelled in red represent sub-landmark populations. CyTOF, cytometry time of flight. PICs, post-intervention controllers. NCs, non-controllers.

**Extended Data Fig. 5:**
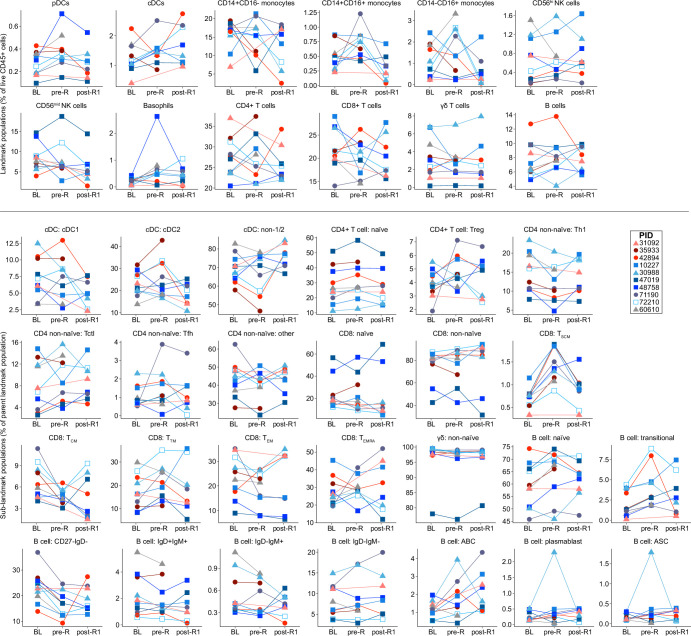
CyTOF landmark and sub-landmark populations. Frequency of major peripheral blood landmark immune cell populations over time (as a % of total live CD45+ cells) and sub-landmark populations over time (as a % of parent landmark populations). Source data

We next investigated whether broad immune cell activation, particularly after the ATI, might be associated with post-ART viral load set point. As unique virologic mechanisms may have contributed to post-intervention control in the aviraemic controller, we focused the following analyses on the six viraemic post-intervention controllers. At baseline, peripheral immune cell type and phenotypic marker frequencies as measured using high-dimensional mass cytometry (CyTOF) as well as broad gene expression patterns as assessed by bulk RNA-sequencing (RNA-seq) analysis of total peripheral blood mononuclear cells (PBMCs) were generally similar between the six viraemic post-intervention controllers and the three non-controllers (Extended Data Fig. 4a,b; see Supplementary Table 7 for the CyTOF panels and Extended Data Figs. 4c and 5 for major immune cell type gating and longitudinal frequencies).

**Fig. 4:**
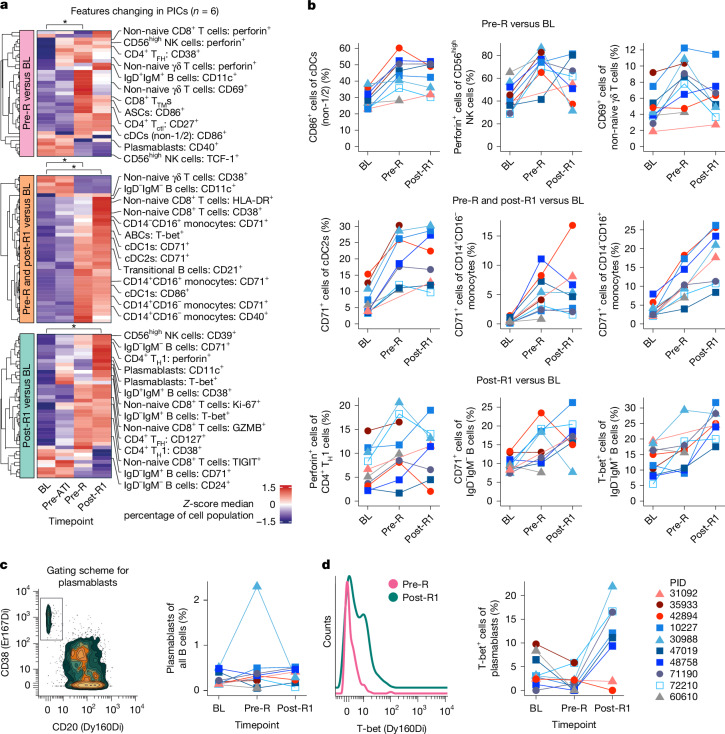
The sequential activation of innate and adaptive immune cells peri-rebound after combination immunotherapy. **a**, The subset of manually gated immune cell features of which the abundance changed significantly in the six viraemic post-intervention controllers (PICs) between baseline compared with pre-rebound (top), baseline compared with the first post-rebound timepoint (bottom) or baseline compared with both timepoints (middle), as measured using CyTOF. Statistical analysis was performed using the two-sided Wilcoxon signed-rank test; *P < 0.05. The features include cell types and phenotypes as listed in Supplementary Table 4; median values are *z* scored by feature. **b**, Participant-level changes in a subset of immune features from **a**; the plots also show data from non-controllers (red) and the aviraemic controller (grey). **c**, Gating (left) and longitudinal assessment (right) of total plasmablast abundance. **d**, Representative plot (left) and longitudinal assessment (right) of the frequency of T-bet^+^ plasmablasts. Timepoint definitions are provided in Fig. 3. For PID 60610 (aviraemic controller), data shown at the pre-R timepoint were generated from samples collected 44 weeks into the ATI. ABC, activated B cells; ASC, antibody-secreting cells; T_H_, T helper cells; T_FH_, T follicular helper cells; T_TM_, transitional memory T cells; T_ctl_, cytotoxic CD4^+^ T cells. Source data

We next examined what changes in broad immune cell types and phenotypes occurred over the course of the trial. We first focused on characterizing longitudinal samples from the six viraemic post-intervention controllers (Wilcoxon signed-rank test for each timepoint versus baseline, *P* < 0.05 was considered to be significant; the full analysis is provided in Supplementary Table 5). There were no significant changes in immune cell type frequencies or phenotypes between baseline and before pausing ART. By contrast, we observed three distinct time-dependent patterns of immune activation after ART was stopped (Fig. 4a). First, some immune cell activation occurred at the last off-ART pre-rebound timepoint (pre-R). This included increases in the frequency of conventional dendritic cells (cDCs, non-cDC1/2 phenotype) expressing CD86 as well as CD56^high^ natural killer (NK) cells expressing perforin (Fig. 4b (top row)). Second, multiple innate and adaptive immune cell types significantly upregulated activation markers (particularly CD71 on innate immune populations) just before and also early into rebound (post-R1; that is, the first PBMC sample available after quantifiable rebound, sampled within 30 days after rebound and before viral loads exceeded 3,000 copies per ml; Fig. 4b (middle row)). Third, multiple cell types exhibited immune activation only after rebound occurred. These changes included the expression of the antiviral transcription factor, T-bet, on non-naive IgD^−^IgM^+^ B cells (Fig. 4b (bottom row)).

For most of the above immune features profiled using CyTOF, viraemic post-intervention controllers and non-controllers showed similar changes compared to baseline during the peri-rebound period (that is, at pre-R and post-R1), whereas minimal changes were observed in the aviraemic controller sampled several months into the ATI (Fig. 4b (blue versus red versus grey lines, respectively); the full dataset is provided in Supplementary Table 5). However, a handful of features appeared to increase uniquely in the controllers at post-R1. Although the overall abundance of plasmablasts did not change consistently in either group (Fig. 4c), the frequency of plasmablasts that express T-bet increased markedly at post-R1 only in the six viraemic post-intervention controllers (median, 1.18% at pre-R versus 14.29% at post-R1; *P* = 0.03) and not in the non-controllers (4.00% versus 0.94%; Fig. 4d).

## Controllers respond robustly to rebound

**Fig. 5:**
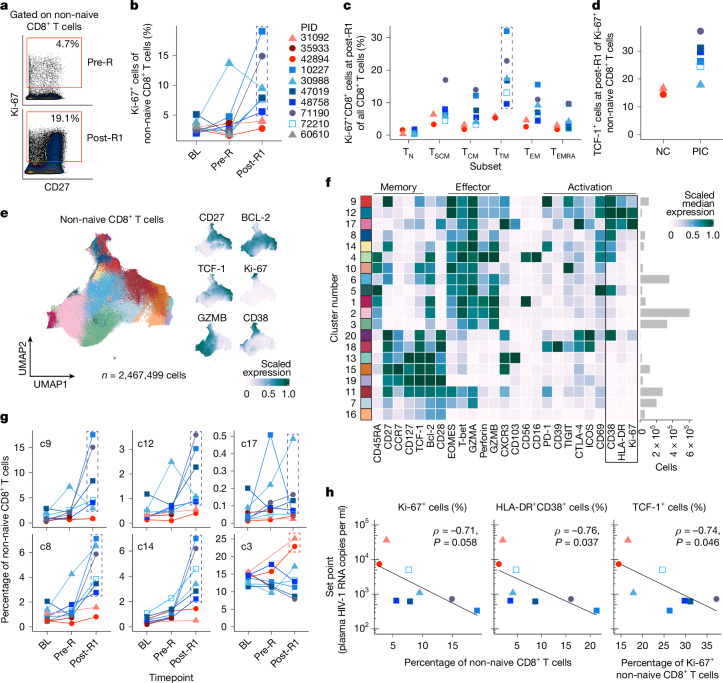
Post-intervention control of HIV is associated with a robust stem/memory-like, activated/proliferating CD8^+^ T cell response to rebound. **a**,**b**, The gating strategy (**a**) and the frequency of Ki-67^+^ non-naive CD8^+^ T cells by manual gating at the baseline, pre-R and post-R1 timepoints as measured using CyTOF (**b**) (timepoint definitions are shown in Fig. 3). **c**, The distribution of Ki-67^+^ cells across CD8^+^ T cell subsets at the post-R1 timepoint. T_N_, naive (CD45RA^+^CCR7^+^CD95^−^); T_SCM_, stem cell memory (CD45RA^+^CCR7^+^CD95^−^); T_CM_, central memory (CD45RA^−^CCR7^+^), T_TM_, transitional memory (CD45RA^−^CCR7^−^CD27^+^), T_EM_, effector memory (CD45RA^−^CCR7^−^CD27^−^), T_EMRA_, CD45RA-expressing effector memory (CD45RA^+^CCR7^−^). **d**, The frequency of TCF-1^+^ cells within the Ki-67^+^ non-naive CD8^+^ T cells at the post-R1 timepoint. **e**, Uniform manifold approximation and projection (UMAP) analysis of non-naive CD8^+^ T cells including cells from all participants at the baseline, pre-ATI, pre-R and post-R1 timepoints (left). Right, expression of individual markers on the UMAP. **f**, The scaled median expression of markers across all clusters (activation markers CD38, HLA-DR and Ki-67 are boxed for emphasis). **g**, The frequencies of clusters from **f** of non-naive activated CD8^+^ T cells across the baseline, pre-R and post-R1 timepoints; c9, c12, c17, c8 and c14 are activated and express CD38 ± HLA-DR ± Ki-67; cells in c3 have a T_EMRA_ phenotype. **h**, Two-sided Spearman correlations between each participant’s post-ART set point viral load and the frequency of select non-naive CD8^+^ T cell populations at the post-R1 timepoint. The analysis excludes PID 60610 (no rebound) and 35933 (no post-R1 sample). The dashed boxes in **b**,** c** and **g** encompass datapoints from post-intervention controllers. Source data

**Extended Data Fig. 6:**
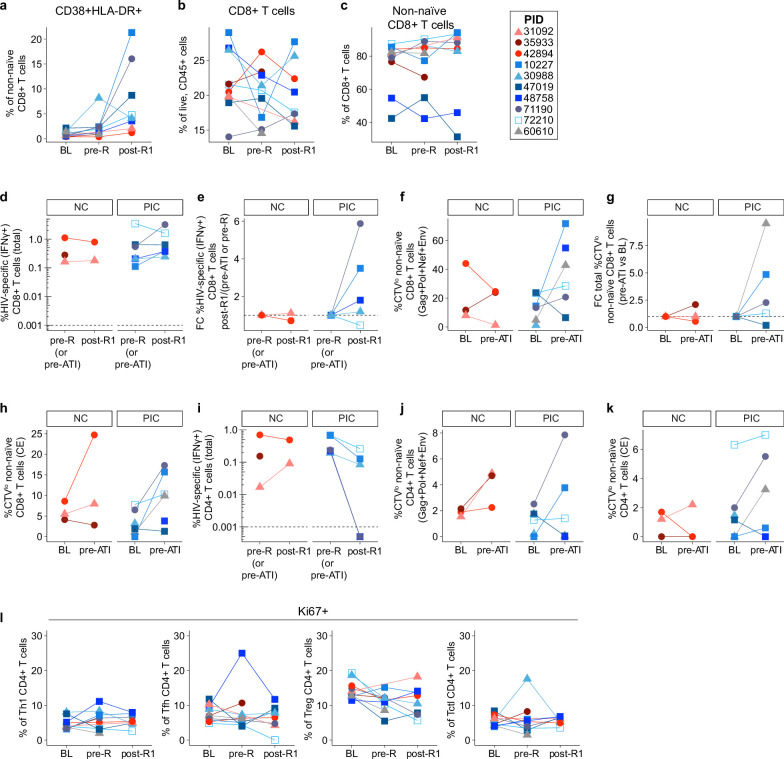
Additional T cell characterization. Longitudinal frequency at baseline (BL), before rebound (pre-R), and/or after rebound (post-R1) of: **a**) Activated (CD38^+^HLA-DR^+^) non-naïve CD8^+^ T cells, **b**) total CD8^+^ T cells, **c**) non-naïve CD8^+^ T cells, **d**) total HIV-specific CD8^+^ T cells (as measured by IFNγ expression after 6-hour in vitro stimulation with HIV Gag, Pol, Nef, or Env peptide pools [responses summed]). **e**) Fold change (FC) between pre-R and post-R1 in HIV-specific CD8^+^ T cell magnitude. **f**) Total HIV-specific CD8^+^ T cell proliferative response (as measured by the dilution of the dye, cell trace violet [CTV], after 6-day in vitro stimulation with HIV peptide pools). **g**) FC between BL and pre-ATI in CD8^+^ T cell proliferation in response to Gag+Pol+Nef+Env peptide pool stimulation. **h**) CE-specific CD8^+^ T cell proliferative response. Magnitude (**i**) or proliferative response (**j-k**) of HIV-specific CD4^+^ T cell responses to Gag+Pol+Nef+Env peptide pools (**i-j**) or CE peptide pool (**k**). **l**) Frequency of Ki67^+^ cells within different CD4^+^ T cell subsets. Within-group differences were not significant in **d**), **f**), **h**), **i**), **j**), **k**) (linear mixed effects models). Source data

Notably, in the above analysis, only post-intervention controllers had a robust increase in the frequency of activated non-naive CD8^+^ T cells expressing Ki-67 (a cell cycle marker) early in response to rebounding virus (median (range), 2.65% (1.96–5.11) at baseline versus 2.85% (1.83–13.71) at pre-R versus 8.69% (5.78–19.11) at post-R1; *P* = 0.03 (post-R1 versus baseline)), whereas the non-controllers did not (2.60% (2.45–3.05) at baseline versus 2.63% (1.54–3.72) at pre-R versus 3.33% (2.72–3.94) at post-R1; Fig. 5a,b). A similar pattern was observed in the controllers using the combination of activation markers HLA-DR and CD38 (Extended Data Fig. 6a). The fraction of Ki-67^+^CD8^+^ T cells at post-R1 in the controllers was highest in cells with a transitional memory phenotype (CD45RA^−^CCR7^−^CD27^+^; Fig. 5c), which is the phenotype of a majority of HIV-specific CD8^+^ T cells in people living with HIV on ART^26^.

Three out of the six post-intervention controllers also exhibited one or more of the following changes in their CD8^+^ T cells at the post-R1 timepoint: an increase in the frequency of total CD8^+^ T cells, total non-naive CD8^+^ T cells and/or HIV-specific CD8^+^ T cells as measured by ICS (Extended Data Fig. 6b–e). Moreover, some post-intervention controllers had an increase between baseline and pre-ATI in HIV-specific CD8^+^ T cell proliferation in response to in vitro stimulation with vaccine-matched (CE) or consensus sequence HIV clade B overlapping peptide pools (Extended Data Fig. 6f–h). However, some of these changes were also observed in the non-controllers. In contrast to the dynamic CD8^+^ T cell responses to rebound, there was no consistent increase in the frequency of HIV-specific CD4^+^ T cells based on ICS or any subset of CD4^+^ T cells expressing Ki-67 at post-R1 (Extended Data Fig. 6i–l).

## Controllers expand TCF-1^+^CD8^+^ T cells

**Extended Data Fig. 7:**
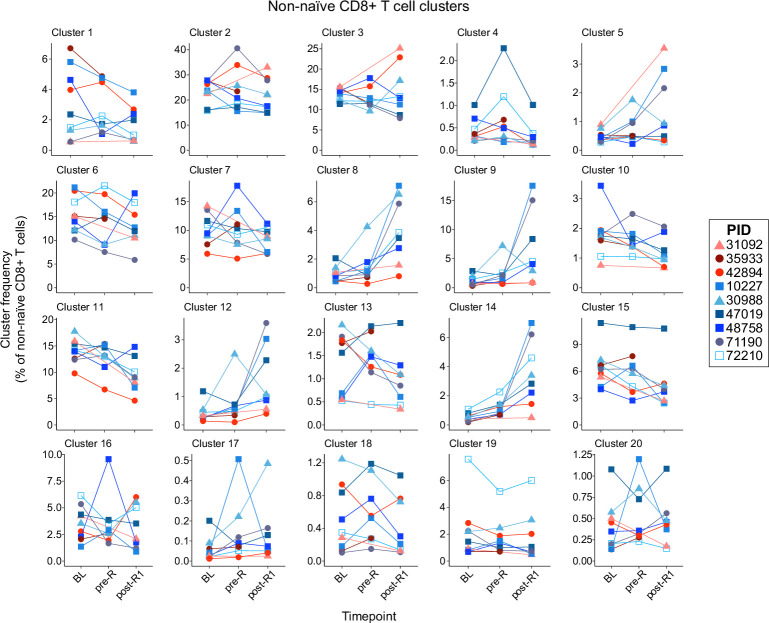
Longitudinal non-naïve CD8^+^ T cell cluster abundances by CyTOF. Source data

We and others have found that individuals who control HIV spontaneously in the absence of ART (elite controllers) have HIV-specific CD8^+^ T cells that express high levels of the T cell-memory-associated transcription factor TCF-1, which supports their ability to robustly proliferate and differentiate into potent antiviral secondary effector cells in response to antigen stimulation^26–29^. Here TCF-1 expression was higher in the responding Ki-67^+^ non-naive CD8^+^ T cells from post-intervention controllers than in those from non-controllers at post-R1 (Fig. 5d). Unbiased clustering of the non-naive CD8^+^ T cells further revealed that, during rebound, post-intervention controllers uniquely expanded multiple distinct, activated (that is, co-expressing CD38 with Ki-67, HLA-DR and/or CD69), CD45RA^−^ non-naive CD8^+^ T cell populations that express some effector-associated proteins (such as granzyme A, T-bet; see cluster 9 (c9), c12, c17, c8 and c14; Fig. 5e–g and Extended Data Fig. 7). Some of these clusters more closely resemble a precursor exhausted phenotype (c9: TCF-1^+^CD27^+^PD-1^+^) whereas others have a more terminally differentiated effector phenotype (c14: perforin^+^granzyme B^+^; c8: perforin^−^granzyme B^+^). Notably, non-controllers did not expand any of these activated non-naive CD8^+^ T cell populations. Instead, across timepoints, they tended to have a larger population of CD45RA^+^CCR7^−^ (T_EMRA_ phenotype) non-naive CD8^+^ T cells that lack classical T cell memory markers (c3; Fig. 5g).

Notably, at post-R1, a higher frequency of activated non-naive CD8^+^ T cells that express Ki-67 or HLA-DR/CD38 as well as a higher frequency of TCF-1^+^ cells within the Ki-67^+^ non-naive CD8^+^ T cells was associated with the subsequent establishment of a low viral load set point post-ART (*ρ* = −0.71, *P* = 0.058; *ρ* = −0.76, *P* = 0.037; *ρ* = −0.74, *P* = 0.046, respectively; Fig. 5h). This is in contrast to the multiple virologic and immunologic factors measured before the ATI that did not correlate with viral load set point after ATI.

## Discussion

In this proof-of-concept mechanistic study of people living with HIV who received a combination immunotherapy regimen, we observed atypical post-ART HIV rebound kinetics in seven out of ten participants. During the post-ART period, one exhibited no detectable rebound for more than 18 months off ART and six exhibited a slow initial rebound rate (slope) followed by heterogeneous patterns of rebound and then viral load control around 1,000 copies per ml for months despite bNAbs waning and/or loss of viral susceptibility to bNAbs. The slope of the rebound in the viraemic controllers was lower than that observed in a concurrent cohort of spontaneous HIV controllers who paused ART without a preceding intervention. The proportion of participants achieving this degree of short-term viral control after pausing ART (7 out of 10) is higher than has been observed in historical cohorts, even among individuals who initiated ART early after HIV acquisition (10–20%)^7–9,11,12,18,22,23,30,31^. Notably, a recent study in NHPs demonstrated comparable rates of SHIV control (7 out of 10) after the administration of a similar combination therapy regimen (that is, therapeutic vaccine, TLR agonist, and bNAbs); rates of control were much lower in the control arm (no intervention, 0 out of 15) and the two arms in which only part of this regimen was administered (9 out of 24)^5^.

We conducted multiple mechanistic studies to understand the contributors to this effect. We found no correlation between the estimated size of the HIV reservoir, bNAb or lefitolimod exposure, or pre-interruption immune responses and the degree of post-intervention control. By contrast, we found a robust expansion of circulating activated Ki-67^+^CD8^+^ T cells in response to rebounding virus exclusively in the viraemic post-intervention controllers. There was a correlation between the frequency of the activated CD8^+^ T cells during rebound as well as their expression of the stem/memory marker TCF-1 and the establishment of a lower viral load set point. Rare control of HIV or SIV without ART or after pausing ART in the absence of other interventions has also been associated with strong proliferative HIV/SIV-specific CD8^+^ T cells^26–28,32,33^. To our knowledge, our study is the first to demonstrate that, after combination immunotherapy, individuals with a more robust in vivo CD8^+^ T cell response to emerging virus after pausing ART go on to establish better control of HIV.

To capture the early differences in CD8^+^ T cell responses to viral rebound, it was critical to evaluate a timepoint after the start of rebound but before viral loads diverged. It is likely that the CD8^+^ T cells that are activated/proliferate in response to rebound (that is, the expanded cells expressing Ki-67 ± HLA-DR ± CD38) are HIV specific, based on studies of acute-phase infections in mice and humans (including acute HIV)^34–36^ and the fact that they are preferentially found within a T cell memory subset typical for HIV-specific CD8^+^ T cells (transitional memory)^26^. Notably, at this early rebound timepoint, controllers expanded populations of both activated TCF-1^+^ stem/memory CD8^+^ T cells as well as more terminally differentiated activated/cytotoxic (granzyme B^+^) CD8^+^ T cells. We hypothesize that the activated TCF-1^+^CD8^+^ T cells initially enable a potent response to the initial burst of viral replication in rebound and subsequently serve as a longer-lived pool of CD8^+^ T cells with regenerative potential that can continually give rise to new cytotoxic effector cells, leading to sustained control. This model is consistent with observations made in mice with chronic infection^37,38^ and in other clinical settings in humans (such as SARS-CoV-2 clearance^39^ or cancer response to immunotherapy^40–42^). In this light, these results support continued efforts towards optimizing therapeutics to generate a population of HIV-specific T cells with regenerative potential to promote effective long-term control of HIV.

These findings raise the important question of how the therapeutic interventions enabled a highly effective CD8^+^ T cell response against HIV rebound in vivo. The DNA/MVA therapeutic vaccine regimen (stage 1) boosted or elicited new HIV-specific CD8^+^ T cells targeting CEs in Gag in nine out of ten participants; these vaccine-elicited T cells may have contributed to viral control. Although we cannot rule out a potential effect of the combination of the TLR9 agonist and the bNAbs (stage 2), we did not detect consistent evidence of latency reversal, reservoir reduction or enhanced immunity. However, the administration of the two bNAbs at the time of treatment interruption (stage 3) was associated with broad innate and adaptive immune activation before rebound. Notably, bNAbs given during periods of virus expression (early ART, post-ART) have been associated with post-ART control in previous clinical trials^10–13^. This may occur either through a classical ‘vaccinal effect’ mediated by bNAb–HIV immune complexes^2,14–17^, and/or because the slow washout of bNAbs given concurrently with ART interruption blunts the typically explosive rebound viraemia, which can impair immune responses. Either of these scenarios might enable boosted and/or newly primed HIV-specific CD8^+^ T cells to out-compete the emerging virus by being more optimally stimulated under low-antigen conditions^7^. While the findings from our study indicate that a combination approach (such as bNAbs with therapeutic vaccination) might prove to be more effective than bNAbs alone, this hypothesis needs to be tested in larger, placebo-controlled, randomized trials.

This study was designed as a proof of concept for an HIV combination immunotherapy approach that included therapeutic vaccination, a TLR agonist and bNAbs. To that end, we targeted enrolment towards people living with HIV who initiated ART early after HIV acquisition, as these individuals generally have lower reservoir sizes, less HIV sequence diversity and preserved immune function^36,43–46^. Notably, the aviraemic controller started ART very early and had the lowest measures of cell-associated HIV DNA or RNA in the cohort. Although we did not target enrolment towards those with any specific genetic features, our cohort also included six participants (including five of the seven post-intervention controllers) with an HLA-B*35 allele. This allele has been associated with more rapid progression of HIV infection^47^ and, recently, with post-treatment control in one large study of post-treatment controllers^31^, although not in another^23^. The possible influence of HLA type on CD8^+^ T and NK cell responses should be explored further. It is our perspective that, even with these favourable characteristics, it is highly unlikely that seven out of ten would have exhibited this degree of post-ART control. However, without these favourable characteristics, the outcomes may have been less impressive.

This study has important limitations. First, as this was the first triple combination immunotherapy study conducted in people living with HIV, and as our aim was to generate the rationale for future, well-powered studies, we chose to conduct a single-arm study. Given the complexity of the combination intervention, the impracticality of maintaining blinding and ethical concerns regarding the use of a placebo group, we felt that a fully powered randomized clinical study would not have been feasible when this study was designed. Such studies are now ongoing or planned (NCT04319367, NCT05245292, NCT04340596, NCT06484335, NCT04983030, NCT06071767, NCT06205602, NCT05719441, NCT05300035). Second, study pauses due to the COVID-19 pandemic as well as the FDA-mandated safety pause resulted in variability in how the second stage was conducted. Still, all of the participants received each of the interventions and viral rebound kinetics did not appear to differ on the basis of variability in the clinical schedules. Third, most of our participants had very low reservoir sizes, limiting the potential to detect changes in HIV RNA or DNA, and limiting the generalizability of our findings. Finally, although we did not identify specific immune or viral features before rebound that predicted post-ART outcomes, this may be due to assay limitations (for example, the in vitro measures of CD8^+^ T cell functional capacity that we tested may not accurately reflect their in vivo response potential) and/or a need to evaluate different immune responses (for example, autologous antibody activity, particularly given the increased T-bet expression in plasmablasts from controllers after rebound).

In this HIV combination immunotherapy trial, we observed a correlation between low post-ART viral load set points and a robust proliferative CD8^+^ T cell response early in the course of viral rebound. This finding provides a proof of concept for the design of future combination immunotherapy interventions in people with the goal of inducing a potent and effective immune response that can achieve sustained ART-free HIV remission.

## Methods

### Participants

For the combination immunotherapy trial (UCSF-amfAR study; NCT04357821), the participants were recruited from the University of California, San Francisco-based SCOPE cohort (NCT00187512), which enrols and prospectively measures adults living with HIV. In brief, interested SCOPE participants completed a screening visit at which their eligibility for the trial was assessed. The trial enrolled adults (aged 18–65 years) with a history of confirmed HIV infection on ART for at least 12 months without any interruptions of more than 14 consecutive days within the preceding year, who were on a stable regimen that did not include a non-nucleoside reverse transcriptase inhibitor for at least the 4 weeks before enrolment. The study was enriched for those who initiated ART during the 6 months after HIV acquisition. Those who were known to have spontaneously controlled HIV before ART initiation were not eligible. Participants were required to have plasma HIV RNA levels below the limit of quantification on all available determinations in the preceding 24 months (blips, or isolated single values above the level of quantification but <200 copies per ml were allowed), with screening CD4^+^ T cell count ≥500 cells per μl. We excluded participants who had a CD4^+^ T cell nadir <350 cells per μl, active hepatitis B or C infection, or other comorbidities (additional screening and exclusion criteria are shown in the Supplementary Methods). Although participants with clinical evidence of pre-ART spontaneous control were ineligible, one individual had an HLA allele associated with spontaneous control of HIV (HLA-B*57:01)^48^. Importantly, we screened for susceptibility to the two bNAbs and excluded those determined to have significantly reduced phenotypic susceptibility to 10-1074 and VRC07-523LS using the PhenoSense monoclonal antibody assay (Labcorp-Monogram Biosciences), defined as IC_50_ values in the top 10% for at least one of the antibodies (10-1074, >50 μg ml^−1^; VRC07-523LS, >0.3834 μg ml^−1^) or in the top 25% for both antibodies (10-1074, >0.1386 μg ml^−1^; VRC07-523LS, >0.2024 μg ml^−1^), based on values derived from a previous assessment in another unrelated study (A5257)^49^.

For the Prospective Observational ATI study (SCOPE-ATI study; NCT04359186), see the Supplementary Methods. Clinical data and biospecimens for both studies were collected between 2020 and 2023.

Both studies were approved by the UCSF IRB. Informed consent was obtained from all of the participants. All of the participants were counselled extensively regarding the risks of HIV transmission during the treatment interruption^50^ and could opt into sociobehavioural research^51,52^. As the studies occurred in the context of the COVID-19 pandemic, extensive plans to address COVID-19 risk mitigation^53^ and accommodate SARS-CoV-2 vaccination^54,55^ were developed.

### Immunotherapy trial design

The study interventions are shown in Fig. 1. At baseline and approximately 4 weeks later, the participants received a DNA vaccine, which included a p24 CE 1/2 plasmid (4 mg) with an IL-12 DNA plasmid (2 mg) split into two separate injections administered through electroporation using the TriGrid Delivery System (TDS-IM v1.0; Ichor Medical Systems). At 12 weeks, they received the p24CE 1/2 plasmid (4 mg) with a p55gag plasmid (2 mg) and IL-12 plasmid (2 mg) split into two separate injections administered through electroporation. At approximately week 20, the participants received an MVA62B boost (10^8^ half-maximum tissue culture infectious dose (TCID_50_) per ml; 1 ml) through intramuscular injection. At week 24, the participants received intravenous infusions of 10-1074 (30 mg per kg) and VRC07-523LS (20 mg per kg). From weeks 25–33, the participants received weekly subcutaneous injections of 60 mg of lefitolimod (2 injections per dose, 1–9 doses; see the ‘Safety of combination immunotherapy’ section and Supplementary Table 2). At approximately week 34, they received a second infusion of each of the two bNAbs and 48 h later interrupted ART. Clinical laboratory values, including plasma HIV RNA levels, were checked weekly from week 34 to 58 and then at least every other week for the remainder of the ATI. ART was reinitiated for any one of the following outcomes: (1) plasma HIV RNA > 50,000 copies RNA per ml for 4 weeks; (2) plasma HIV RNA > 10,000 copies RNA per ml for 6 weeks; (3) plasma HIV RNA > 2,000 copies RNA per ml for 12 weeks; or (4) plasma HIV RNA > 400 copies RNA per ml for 24 weeks. ART could also be reinitiated for confirmed CD4^+^ T cell decline below 350 cells per µl, the presence of severe acute retroviral syndrome, concerns about HIV transmission risk, acute SARS-CoV-2 infection or need for COVID-19 or Mpox vaccination, or at the request of the participant or their primary care clinician. After reinitiation of ART, the participants were seen at approximately 1, 2 and 6 months to confirm virologic suppression.

### Clinical measurements

At each visit, the participants underwent an assessment for adverse events, which were categorized in terms of relatedness (definitely, probably, possibly or not related) and severity using the NIH Division of AIDS Table for Grading the Severity of Adult and Pediatric Adverse Events, Corrected Version 2.1 (July 2017). Blood was collected for safety testing as outlined in the Supplementary Methods. Plasma HIV RNA levels were initially measured using the Abbott Real Time HIV-1 PCR assay (limit of quantitation 40 copies per ml) and subsequently with the Hologic Aptima assay (limit of quantitation, 30 copies per ml); for the purposes of this analysis, all plasma HIV RNA levels <30 copies per ml were considered to be unquantifiable. All laboratory measurements were performed in the clinical laboratory at Zuckerberg San Francisco General Hospital. The beginning of viral rebound (day 0) was defined as the first day of two consecutive quantifiable plasma viral loads. Peak viral load after rebound was defined as the maximum viral load within the first 6 weeks after time of rebound. The viral load rebound slope was defined as the slope of a linear regression between time of rebound and time of peak viral load for each participant. Post-rebound viral set points were defined as the median of the viral loads measured starting 2 weeks after the peak viral load until ART restart. The protocol defined post-treatment control as maintaining a viral load ≤400 copies per ml at two-thirds of the timepoints for a period of ≥24 weeks, consistent with the definition used in the CHAMP study of post-treatment control^22^. For our analyses, we also applied a definition of post-intervention control consistent with literature that emerged since the initial design of the study and has been used to interpret clinical outcomes across multiple trials, as a set point of around 1,000 copies per ml^7^.

See Supplementary Table 2 for participant-level viral load outcomes and Supplementary Table 3 for complete post-ATI viral load dataset.

### Biospecimen collection and storage

At most study visits, peripheral blood was collected by standard blood draw or leukapheresis and stored as serum, plasma and viable cryopreserved PBMCs, as described previously^26^. Serum and plasma were stored at −80 °C and PBMCs were stored in liquid nitrogen.

### Plasma levels of antiretroviral drugs during ART interruption

For 2–3 timepoints per participant throughout the study-defined ART interruption, plasma antiretroviral drug levels were measured using NIH Clinical Pharmacology Quality Assurance (CPQA)-approved high-performance liquid chromatography–tandem mass spectrometry (LC–MS/MS) or ultra-high-performance LC with a photodiode array detector (UPLC–PDA) methods, previously described (tenofovir^56^) and as described in the Supplementary Methods (emtricitabine and dolutegravir assays).

### 10-1074 and VRC07-523LS levels and susceptibility testing

The 10-1074 and VRC07-523LS PK assays were performed at the NIH Vaccine Research Center, using a previously described Meso Scale Discovery-based platform and as described in the Supplementary Methods^57^. The PhenoSense monoclonal antibody assay (Labcorp-Monogram Biosciences) was used to evaluate susceptibility of autologous HIV to neutralization by 10-1074 and VRC07-523LS. Full-length envelope sequences were amplified from cell-associated HIV DNA at baseline or plasma-derived HIV RNA after rebound and cloned into an envelope expression vector. The assay could not be performed on baseline samples from three participants (PIDs 31092, 10227, 60610), presumably owing to low reservoir size. Pseudovirion producer cells were co-transfected with envelope expression vectors and a HIV genomic reporter vector, with *env* replaced by luciferase. Luciferase reporter pseudovirions were collected and tested for susceptibility to 10-1074 and VRC07-523LS in a cell-based neutralization assay. The concentration of antibody required to inhibit infection by 50% or 90% (IC_50_, IC_90_) as well as the maximum percentage inhibition was assessed (the full dataset is provided in Supplementary Table 3).

### HIV DNA and RNA measurements

CD4^+^ T cells were isolated from cryopreserved PBMCs (5 × 10^6^ to 3 × 10^7^ cells) by immunogenic negative selection (StemCell Technologies) and then counted. Total cellular DNA and RNA were extracted from each sample using TRI Reagent (Molecular Research Center). The extracted DNA samples were fragmented using QIAshredder columns (Qiagen)^58^. DNA and RNA concentrations were measured using the NanoDrop One microvolume spectrometer (Thermo Fisher Scientific). The IPDA was performed in a duplex ddPCR assay (FAM and VIC), as described previously (450–750 ng of DNA input per well, 7–8 replicates per sample)^24,59^, and HIV transcription profiling was performed using a modification of previously described methods^60^. Both assays are described fully in Supplementary Methods.

### ICS analysis

The magnitude and phenotype of HIV-specific CD4^+^ and CD8^+^ T cells was characterized by flow cytometry using ICS. Cryopreserved PBMCs were thawed and rested overnight at 37 °C 5% CO_2_. One million cells per condition were incubated for 6 h at 37 °C under 5% CO_2_ with HIV peptide pools at a concentration of 1 μg ml^−1^ per peptide (full or individual smaller CE peptide pools (provided by B.K.F.), or HIV clade B overlapping peptide pools (Gag, Pol, Nef, or Env; from BEI Resources NIH/NIAID)), SEB (positive control) or R10 medium plus DMSO (negative control) in the presence of brefeldin A (Sigma-Aldrich), monensin (Sigma-Aldrich) and anti-CD107a antibodies (BD Biosciences). Cells were then collected and first stained in PBS with viability stain (Zombie UV; BioLegend) for 10 min at 25 °C and then with surface staining markers in PBS + 1% FBS with Brilliant Stain Buffer Plus (BD Biosciences) for 30 min at 25 °C. After surface staining, cells were fixed and permeabilized for 45 min at 4 °C and then stained with intracellular antibodies overnight at 4 °C using the eBioscience FOXP3/Transcription Factor Staining Buffer Set according to the manufacturer’s protocol (Thermo Fisher Scientific). Flow cytometry panels are shown in Supplementary Table 7. The samples were resuspended in PBS + 1% PFA and stored at 4 °C before acquiring on a Cytek 5L Aurora (Cytek Biosciences) using SpectroFlo (v.3.0.3). Compbead anti-mouse, -rat and -hamster particles (BD Biosciences) were stained as reference controls for unmixing except that heat-killed PBMCs were used as a Zombie UV staining unmixing reference. Longitudinal samples from the same participants were run on the same experiment day. To measure interexperiment variability, PBMCs from the same donor without HIV were stimulated with SEB (or incubated with DMSO) and stained in parallel for each experiment. HIV-specific T cell responses were measured based on the fraction of total CD4^+^ or CD8^+^ T cells that expressed IFNγ after peptide stimulation and after subtraction of background IFNγ staining present in a control unstimulated well containing only an equivalent concentration of DSMO (FlowJo v.10, BD Biosciences). Total HIV-specific T cell responses were calculated based on the summed IFNγ^+^ cells after stimulation with Gag-, Pol-, Nef- or Env-overlapping peptide pools.

### In vitro HIV-specific T cell proliferation

Thawed PBMCs in R10 medium were rested overnight at 37 °C under 5% CO_2_. Cells were then labelled with 0.5 mM cell trace violet (CTV) (Thermo Fisher Scientific) according to the manufacturer’s instructions. One million CTV-labelled cells were stimulated for 6 days at 37 °C under 5% CO_2_ in R10 medium plus DMSO (negative control) or R10 containing 0.1 μg ml^−1^ HIV peptide pools composed of peptides derived from Gag CE sequences, HIV clade B overlapping peptide pools (as described above; from BEI Resources) or broad T cell activation as a positive control (ImmunoCult human CD3/CD28 T cell activator; StemCell Technologies) according to the manufacturer’s instructions. After stimulation, the cells were stained for surface proteins and then intracellular proteins and then analysed as described above (on the Cytek Aurora system running SpectroFlo v.3.2.1). Flow cytometry panels are provided in Supplementary Table 7. Proliferative responses were measured as the fraction of CD4^+^ or CD8^+^ T cells that diluted CTV (that is, the percentage of CTV^lo^ cells) after stimulation with HIV peptide pools. Background proliferation present in the negative control wells was subtracted.

### CyTOF analysis

CyTOF analyses were performed over the course of five separate experiments, as described previously^61^. PBMCs were thawed and only samples with over 70% viability were used for analysis. We stained 2–4 million cells per panel in two mass cytometry panels (CyTOF panels are shown in Supplementary Table 7) according to a previously published protocol^62^ with modifications as described in the Supplementary Methods.

### Bulk RNA-seq analysis

Bulk RNA-seq analysis was performed on PBMCs from the baseline timepoint that had been cryopreserved and then thawed. RNA was isolated using the RNeasy Micro Kit (Qiagen) according to the manufacturer’s instructions. Total RNA was quantified using the Qubit RNA Assay Kit and the RNA quality was assessed using the Agilent 4200 TapeStation instrumentation (Agilent). mRNA-seq libraries were constructed using the KAPA mRNA HyperPrep Kit with an input of 50 ng total RNA. The Qubit DNA assay kit was used to determine library concentration and the Agilent 4200 TapeStation instrumentation was used to determine the library size distribution and quality. Equimolar amounts of sample were pooled before loading onto the Illumina NovaSeq 6000 instrument (Illumina) for sequencing. The samples were demultiplexed using bcl2fastq (Illumina). Adapters with low-quality ends were trimmed from FASTQ files using Trim Galore (v.0.6) and quality analysis performed using FastQC (v.0.11.2). Reads mapping to ribosomal RNA and globin were removed using Bowtie2 (v.2.4.2)^63^, resulting in 25 million reads per sample for further analyses. The remaining sequences were aligned to the human GRCh38 genome (Ensembl v112) using STAR (v.2.7.10b)^64^. Alignment results showed that over 90% of reads mapped to the human genome.

### Statistical analyses

#### Slope of HIV rebound modelling

Linear mixed effects modelling was performed in R (v.4.3) using the lmerTest package (v.3.1; two-sided *t*-test with Satterthwaite’s approximation for d.f.) for viral rebound curves from the time of rebound to the time of peak viral load (as defined above: the highest viral load within the first 6 weeks after rebound) or the time of ART restart if that came first, with fixed effects for the post-rebound group on the slope of rebound and random effects for between-participant variability.

#### PK and PK–PD analyses

We described plasma bNAb PK using population PK modelling approaches (including data from all ten participants) in Monolix software (Lixoft v.2023R1) using one- and two- compartment models for 10-1074 and VRC07-523LS, respectively, including participant-specific variation in clearance (both bNAbs) and volume of the central compartment (VRC07-523LS only). Residual variability was described using a proportional error model. Model details are included in Supplementary Table 8. We performed two-sided Spearman’s correlations to determine the relationship between bNAb exposure (area under the concentration-time curve, AUC; peak concentration, *C*_max_), bNAb susceptibility (post-rebound IC_90_) and rebound kinetics (time to rebound, post-ART set point and slope of rebound for the nine participants who experienced viral rebound).

#### ICS and T cell proliferation analysis

Changes in frequencies over time were determined using linear mixed-effect analysis with two-sided Tukey’s multiple-comparison test (GraphPad Prism v.10). The full background-subtracted dataset is provided in Supplementary Table 5.

#### CyTOF manual gating analysis

After data acquisition, normalization and debarcoding of samples was performed using Premessa (v.0.3.4; https://github.com/ParkerICI/premessa). FCS files were imported into CellEngine (CellCarta). Traditional hierarchical gating was applied to identify 12 standard^61^ landmark immune populations and 27 sub-landmark populations (see Extended Data Fig. 4c for gating strategy and Extended Data Fig. 5 for the identity and longitudinal frequency of these populations). Within each of the parent cell types, we manually gated positive and negative populations of biologically relevant phenotypic markers from the two mass cytometry panels (the full dataset is provided in Supplementary Table 5). For each of the parent cell types, we only included phenotypic markers for which we could clearly gate a positive population above the background antibody staining levels. Batch effects were minimized by using the same PBMC control samples in each experiment from people without HIV. For the final analysis, we excluded phenotypic features that were present at very low frequencies (that is, we included only phenotypic features for which at least 10% of the samples had >5% marker positive; a total of 303 features were included in the testing). To determine significant changes in the manually gated landmark, sub-landmark and phenotypic features over time, the frequency of each feature as the percentage of the parent population for each sample was exported and log_10_-transformed with a constant of 1/1 × 10^6^. Two-sided Wilcoxon signed-rank tests were performed on the log-transformed values for timepoint comparisons (pre-ATI, pre-R or post-R1 each with baseline) for the six viraemic post-intervention controllers only. Owing to the small sample size, we did not adjust for covariates, and the significant *P* values for the changes in features over time were not adjusted using multiple-testing correction methods.

#### CyTOF clustering analysis of non-naive CD8^+^ T cells

For clustering analysis of non-naive CD8^+^ T cells, all manually gated live CD45^+^ single cells were downloaded as .fcs files from CellEngine and first batch corrected using cyCombine (v.0.2.19; https://github.com/biosurf/cyCombine). Batch-corrected files were re-uploaded to CellEngine and gated on total CD8^+^ T cells. Non-naive CD8^+^ T cell clusters were selected for downstream analysis. The FCS files containing non-naive CD8^+^ T cells were used in downstream clustering analysis using Catalyst (v.1.28.0; https://github.com/HelenaLC/CATALYST), which performs clustering using FlowSOM and ConsensusClusterPlus. Detailed clustering and visualization settings are provided in the Supplementary Methods.

#### Bulk RNA-seq differential gene expression analysis

Statistical processing and analysis of RNA-seq count data was performed in the R statistical computing environment (R core team 2023). Genes were filtered by a cross-sample mean of 50 or greater and then normalized using edgeR with calcNormFactors using trimmed mean of *M* values^65^ normalization and log_2_-normalized using voom^66^. Differential expression analysis was performed on baseline samples between viraemic controllers (*n* = 6) and non-controllers (*n* = 3) using lmfit through limma (v.3.1)^67^. Significance cut-offs were set at log_2_-transformed fold change of >1.5 and nominal *P* value < 0.05.

### Reporting summary

Further information on research design is available in the Nature Portfolio Reporting Summary linked to this article.

## Online content

Any methods, additional references, Nature Portfolio reporting summaries, source data, extended data, supplementary information, acknowledgements, peer review information; details of author contributions and competing interests; and statements of data and code availability are available at 10.1038/s41586-025-09929-5.

## Supplementary information

Supplementary File 1 Study protocol (redacted).

Reporting Summary

Supplementary Methods Supplementary Methods.

Supplementary Table 1 Adverse Events determined to be definitely, probably or possibly related to the vaccine regimen (A), Lefitolimod, VRC07-523LS, or 10-10741 (B); or that occurred during the ATI (C); or that were determined to be not related (or probably not related) to the study interventions (D).

Supplementary Table 2 Participant-level clinical and outcome data.

Supplementary Table 3 Post-ATI viral load, PK and bNAb susceptibility data (full time course for all participants).

Supplementary Table 4 Statistical testing results comparing HIV rebound slopes (two-sided *t*-test with Satterthwaite’s approximation for degrees of freedom as part of a linear mixed effects model), related to Figure 1e.

Supplementary Table 5 Complete reservoir measurements, flow and mass cytometry data and analysis.

Supplementary Table 6 Correlation analyses (two-sided Spearman’s test): pre-ATI viral reservoir and HIV-specific T cell measurements versus post-ART set points.

Supplementary Table 7 Flow and mass cytometry panels.

Supplementary Table 8 bNAb population PK model parameters.

## Source data

Source Data Fig. 1

Source Data Fig. 2

Source Data Fig. 3

Source Data Fig. 4

Source Data Fig. 5

Source Data Extended Data Fig. 2, 3, 5, 6, 7

## Figures and Tables

**Extended Data Table 1:**
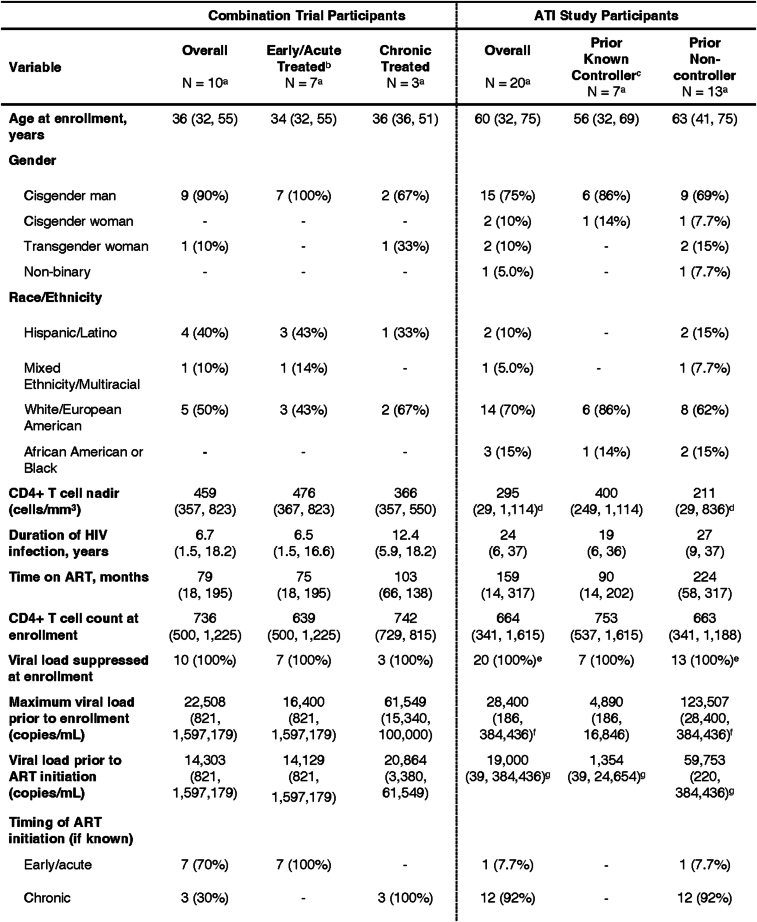
Participant characteristics

Footnotes: ^a^Median (Range); n (%). ^b^Three participants began antiretroviral therapy during the acute phase of infection (“acute”; <30 days after estimated HIV acquisition and likely prior to peak viremia) and four participants began antiretroviral therapy between 1 and 6 months after HIV acquisition (“early”). ^c^Of the 7 prior known controllers in the observational ATI study, 2 were previously elite controllers and 5 were low viremic controllers (sustained viral loads <2,000 copies/mL). ^d^Data on CD4^+^ T cell nadir were missing from 2 individuals in the prior non-controller group. ^e^A single individual in the prior non-controller group had a transient blip of 67 copies/mL at enrolment. ^f^Data on maximum viral load prior to enrolment were missing from 5 prior non-controllers. ^g^Data on viral load prior to ART initiation were missing from 1 prior known controller and 4 prior non-controllers.

## Data Availability

Bulk RNA-seq data are available at the Gene Expression Omnibus (GSE288962). The human genome used for RNA-seq alignment (GRCh38, Ensembl) is available online (https://www.ncbi.nlm.nih.gov/datasets/genome/GCF_000001405.26/). Full processed (gated and/or clustered) mass cytometry and flow cytometry data are provided in Supplementary Table 5. De-identified raw .fcs files with mass cytometry data were deposited at Mendeley (10.17632/3wfkd6rrht.1). Raw flow cytometry .fcs files will be made available on request. Source data are provided with this paper.

## Code availability

Bulk RNA-seq code is available on the Gale Laboratory GitHub page (https://github.com/galelab and https://github.com/galelab/Rutishauser_post_intervention_HIV_control).
