# Supplementary material for: Correlates of HIV-1 control after combination immunotherapy
Source: Nature. 2025 Dec 1;650(8100):187–95. doi: 10.1038/s41586-025-09929-5 (PMC12872443; doi:10.1038/s41586-025-09929-5)
Supplement: Supplementary file 1 — Study protocol (redacted). [file 41586_2025_9929_MOESM1_ESM.pdf]

nature portfolio

https://doi.org/10.1038/s41586-025-09929-5

Supplementary information

Correlates of HIV-1 control after combination immunotherapy

In the format provided by the authors and unedited

Clinical Research Protocol

Combinatorial therapy with a therapeutic conserved element DNA vaccine, MVA vaccine boost, TLR9 agonist and broadly neutralizing antibodies:

A proof-of-concept study aimed at inducing an HIV remission

Protocol Number:	18-26957
Version Date:	Version 2.2
Investigational Product:	• HIV DNA (p24CE and p55gag) and IL-12 DNA plasmids<br>• MVA/HIV62B (MVA62B)<br>• TLR9 agonist (lefitolimod)<br>• Combination bNAbs (10-1074 and VRC07-523LS)
IND Number:	18488
Development Phase:	Phase I/IIA
Sponsor:	University of California, San Francisco
Funding Organization:	amfAR
Principal Investigator:	Steven G. Deeks
Coordinating Center:	UCSF Data Coordinating Center

Approval:

PI or Sponsor Signature (Name and Title) ____________________ Date ____________

This confidential information about an investigational product is provided for the exclusive use of investigators of this product and is subject to recall at any time. The information in this document may not be disclosed unless federal or state law or regulations require such disclosure. Subject to the foregoing, this information may be disclosed only to those persons involved in the study who have a need to know, with the obligation not to further disseminate this information.

Protocol Signature Page

I have read the protocol specified below. In my formal capacity as Investigator, my duties include ensuring the safety of the study subjects enrolled under my supervision, as outlined in the protocol. It is understood that all information pertaining to the study will be held strictly confidential and that this confidentiality requirement applies to all study staff at this site. I will conduct the study in accordance with the provisions of this protocol and all applicable protocol-related documents. I agree to conduct this study in compliance with United States (US) Health and Human Service regulations (45 CFR 46); applicable U.S. Food and Drug Administration regulations; standards of the International Conference on Harmonization Guideline for Good Clinical Practice (E6); Institutional Review Board/Ethics Committee determinations; all applicable in-country, state, and local laws and regulations; and other applicable requirements (e.g., US National Institutes of Health, Division of AIDS) and institutional policies.

____

Signature of Investigator of Record **Date (Month/Day/Year)**

____

Investigator of Record (PRINT NAME)

Full Protocol Title: Combinatorial therapy with a therapeutic conserved element DNA vaccine, MVA vaccine boost, TLR9 agonist and broadly neutralizing antibodies: A proof-of-concept study aimed at inducing an HIV remission

Protocol Date: 09032019

LIST OF ABBREVIATIONS

AA	amino acids
ADDC	Antibody-dependent cellular cytotoxicity (ADCC)
AE	Adverse event
ALT	Alanine aminotransferase
ALVAC	canary pox virus
ART	Antiretroviral therapy
AST	Aspartate aminotransferase
ATI	Analytic treatment interruption
ATI	Analytic treatment interruption
BGH	bovine growth hormone
bNAb	broadly neutralizing antibody
BUN	Blood urea nitrogen
CDC	Centers for Disease Control and Prevention
CE	conserved element
CMV	cytomegalovirus
COT-M	HIV-1 Group M Center-of-Tree
CPK	creatine phosphokinase
CRF	Case report form
CRP	C-reactive protein
CTL	cytotoxic T lymphocyte
DAIDS	Division of AIDS (NIAID)
DMC	Data Monitoring Committee
DSMB	Data Safety Monitoring Board
ECL	electrochemiluminescence
EDTA	ethylenediaminetetraacetic acid
ELISA	enzyme-linked immunosorbent assay
EP	in vivo electroporation
EP	Electroporation
FDA	Food and Drug Administration
Gag	group specific antigen (capsid proteins)

GCP	Good Clinical Practice
GGT	Gamma-glutamyl transferase
GLP	Good Laboratory Practice
GM-CSF	granulocyte-macrophage colony-stimulating factor
HBV	hepatitis B virus
hCMV	human cytomegalovirus
HIPAA	Health Insurance Portability and Accountability Act of 1996
HIV	human immunodeficiency virus
HLA	human leukocyte antigen
HPV	human papilloma virus
HVTN	HIV Vaccine Trials Network
HXB2	Reference HIV-1 genome sequence
ICH	International Conference on Harmonisation
ICS	intracellular cytokine staining
IFN-γ	IFN-γ
IL-12	interleukin-12
IM	Intramuscular
IRB	Institutional Review Board
IV	Intravenous
MAb	Monoclonal antibody
MVA	Modified Vaccinia virus Ankara
p15Gag	proteolytic processing intermediate of the HIV-1 p55Gag precursor protein, consisting of p2 (spacer peptide 1); nucleocapsid protein, p7; p1 (spacer peptide 2,) and p6 protein; located at C-terminus of p55gag precursor
p17Gag	proteolytic processing product (matrix protein) of HIV-1 p55Gag located at N terminus of the p55Gag precursor
p24CE pDNA	general term defining the vaccine consisting of the mixture of 2 DNA plasmids expressing p24CE1 & p24CE2 proteins or the dual promoter plasmid p24CE1/2 expressing both p24CE1 and p24CE2 proteins
p24CE1	pDNA vaccine encoding the p24CE1 protein, consisting of seven selected highly conserved segments of the HIV-1 p24Gag capsid protein; p24CE1 differs by 7 AA, 1 AA per CE, from the p24CE2 protein

p24CE1/2	multivalent HIV-1 M group CE pDNA vaccine encoding 2 proteins (p24CE1, p24CE2), each consisting of seven selected highly conserved segments of the HIV 1 p24Gag capsid protein
p24CE2	pDNA vaccine encoding the p24CE2 protein, consisting of seven selected highly conserved segments of the HIV-1 p24Gag capsid protein; p24CE2 differs by 7 AA, 1 AA per CE, from the p24CE1 protein
p24Gag	proteolytic processing product (capsid protein) of HIV-1 p55Gag; located between p17 and p15 of the p55Gag precursor protein
p27CE pDNA	general term defining the vaccine consisting of the mixture of 2 DNA plasmids expressing HIV p27CE1and p27CE2 proteins
p27CE1	pDNA vaccine encoding the p27CE1 protein, consisting of seven selected highly conserved segments of the SIV-1 p27Gag capsid protein; p27CE1 differs by 6 AA, from the p27CE2 protein
p27CE2	pDNA vaccine encoding the p27CE2 protein, consisting of seven selected highly conserved segments of the SIV-1 p27Gag capsid protein; p27CE1 differs by 6 AA, from the p27CE2 protein
p27Gag	proteolytic processing product (capsid protein) of SIV p57Gag; located between p19 and p15 of the p57Gag precursor protein
p55gag pDNA	pDNA vaccine encoding the unprocessed HIV-1 p55Gag precursor protein
p57gag pDNA	pDNA vaccine encoding the unprocessed SIV p57Gag precursor protein
PBMC	peripheral blood mononuclear cell
pDNA	plasmid DNA (or DNA plasmid)
PI	Principal Investigator
PK	Pharmacokinetic
polyA	polyadenylation signal
PRD	Participant Reminder Diary
RM	rhesus macaque
RNA	ribonucleic acid
SAE	Serious adverse event
SIV	simian immunodeficiency virus
SP	signal peptide
SV40	simian vacuolating virus 40
TDS-IM	TriGrid™ Delivery System-Intramuscular
VEEV	Venezuelan equine encephalitis virus

VRC	Vaccine Research Center, NIAID
VSV	Vesicular stomatitis virus

PROTOCOL SYNOPSIS

**TITLE**	Combinatorial therapy with a conserved element DNA vaccine, MVA vaccine boost, TLR9 agonist and broadly neutralizing antibodies: A proof-of-concept study aimed at inducing an HIV remission
**SPONSOR**	University of California, San Francisco
**PRINCIPAL INVESTIGATOR**	Steven G. Deeks, MD
**FUNDING ORGANIZATION**	amfAR
**NUMBER OF SITES**	One
**RATIONALE**	Combination approaches will almost certainly be required to generate durable control of HIV in the absence of antiretroviral therapy (a "remission").<br><br>Based on studies in "elite" controllers and other chronic virus infections, durable control of HIV will likely require potent and sustained HIV-specific CD8+ T cells that target conserved epitopes. A DNA plasmid vaccine designed to generate subdominant responses to conserved regions within gag (the "p24 Conserved Element", or p24CE vaccine) has demonstrated impressive immunogenicity in macaques, particularly when boosted with a viral vector (MVA).<br><br>In a recent study of SIV-infected macaques on effective antiretroviral therapy, a therapeutic vaccine strategy alone was insufficient in preventing SIV rebound after antiretroviral therapy (ART) was discontinued. In contrast, this same vaccine strategy followed by a TLR7 agonist resulted in durable control of SIV in a subset of animals.<br><br>A recent unpublished study also demonstrated that the combination of a bNAb and a TLR7 agonist during ART can reduce and apparently eliminate the reservoir through mechanisms that have yet to be defined.<br><br>Recent published data from the SIV non-human primate model suggests that bNAbs can alter the immune response of untreated animals if present during acute bursts in viremia (acute infection). Strong and effective CD8+ T cell responses are generated in this setting, presumably as the bNAbs and HIV antigens generate immune complexes that stimulate antigen-presenting cells.<br><br>A number of studies of elite and post-treatment controllers suggest that clearance of HIV during ART or control of HIV after ART may require potent NK cell responses, in addition to potent CD8+ T cell responses. Env-specific antibodies capable of stimulating antibody-dependent cellular cytotoxicity (ADCC) by recruiting NK cells and other effector responses may also be critical, suggesting that any effective therapeutic

	curative study will need to enhance such responses. A common approach to enhance these responses is to co-administer an immunomodulatory (such as TLR9 agonist) with bNABs, hence generating a potent NK cell response and the capacity for ADCC.
**STUDY DESIGN**	A single arm study of twenty HIV-infected adults on effective ART will be performed.<br><br>All subjects will receive a combination regimen administered during ART and during an analytic treatment interruption. Our strategy has five stages; (1) IL-12 adjuvanted p24CE DNA prime (p24CE/IL-12) at Weeks 0 and 4, (2) IL-12 adjuvanted DNA boost (p24CE plus p55gag) at Week 12, (3) MVA/HIV62B (MVA62B) boost at Week 20, (4) single dose of two bNAbs (VRC07-523LS and 10-1074, which target CD4 binding site and V3 loop, respectively) at week 24 with a TLR9 agonist (lefitolimod) administered weekly between Weeks 25 and 33 (9 doses), (5) ATI with single dose of VRC07 and 10-1074 at Week 34 with follow-up off ART through at least Week 46 (expected) and on or off ART (depending on outcome) through Week 86.<br><br><u>Stage 1:</u> Subjects will first be vaccinated with IL-12 adjuvanted HIV DNA vaccines expressing 7 unique conserved elements (CE) from p24 (gag). HIV vaccine and IL-12 DNA will be administered by electroporation at Weeks 0 and 4. The goal is to generate responses to conserved regions while avoiding induction or expansion of immunodominant responses to more variable regions.<br><br><u>Stage 2:</u> These responses will then be boosted with IL-12 adjuvanted DNA vaccine containing p24CE and full-length gag (p55) at Week 12. This strategy is expected to boost CE-specific T cell response magnitude and breadth.<br><br><u>Stage 3:</u> Subjects will receive an MVA boost at Week 20; this vaccine is expected to further boost the CD8+ T cell responses.<br><br><u>Stage 4:</u> A single dose of two bNAbs (VRC07-523LS and 10-1074) will be administered intravenously at Week 24, with therapeutic levels expected to persist through at least Weeks 32-34. Starting at Week 25 and continuing through Week 33, subjects will receive a series of a TLR9 agonist (lefitolimod), administered weekly by subcutaneous injection. This stage has three goals: to enhance DNA/MVA vaccine responses, to generate potent NK cell responses and to reverse latency (which will allow vaccine-induced T cells and bNAbs to reduce the reservoir size).<br><br><u>Stage 5:</u> Subjects will discontinue ART at Week 34. An additional dose of the bNAbs will be administered ~~on~~ two days prior to treatment interruption. Therapeutic bNAb levels (and continued viral suppression) are expected through at least Week 42. The goal of this

	stage is to continue reservoir reduction (ADCC activities during pre-viral rebound state) and to enhance CTL responses (during the post-viral rebound state, when antibodies will capture and present antigen to APCs).<br><br>Should this approach work, viral load would be expected to rebound in all individuals a few weeks after the bNAbs levels decrease to subtherapeutic levels. This acute rebound would be followed by a new lower viral load set-point and perhaps a long-term remission.
**PRIMARY OBJECTIVE**	• To determine the safety and tolerability of combination regimen including a DNA vaccine, MVA boost, TLR9 agonist and bNAbs<br>• To determine the impact of this 5-stage regimen on post-treatment control of viral load during a treatment interruption
**SECONDARY OBJECTIVES**	• To determine the immunogenicity of this 5-stage regimen<br>• To determine the anti-reservoir activity of this this 5-stage regimen
**RATIONALE FOR TARGET POPULATION**	We carefully considered the ideal target population for this combination trial to maximize the impact of the study. Several lines of evidence from observational studies and randomized controlled trials suggest that individuals starting ART early (e.g., within the first 6 months of infection) have reduced chronic immune activation, improved adaptive immune function, and lower HIV reservoir size than individuals starting ART late (e.g., after 2 years of infection). For all these reasons, early ART individuals would be expected to have a more robust immunologic response to a therapeutic vaccine and have a more manageable reservoir size to contain (and eliminate) with the TLR9 agonist/bNAb strategy than those starting ART late. Indeed, the remission/cure outcomes noted in the non-human primate studies that provide the rationale for this study were all performed in animals treated during the acute phase of the infection. Thus, to establish proof of principle that this therapeutic strategy can contain and/or eliminate reservoirs in the absence of therapy, we will primarily enroll individuals who started ART early. We also recognize that there are practical limitations to enrolling a full 20 participants who started ART this early and recognize the value of testing this intervention in at least some individuals who started ART in later stages of infection to begin to address the potential generalizability of our therapeutic strategy in the vast majority of HIV-infected individuals who started ART late. We thus chose to focus on early ART participants for our primary analysis (and powering the study to detect clinically meaningful levels of post-treatment control) and include a small subset of chronically infected individuals for an exploratory analysis to develop preliminary data that might inform the next set of studies in chronically infected

	individuals should our strategy be successful in those who start early. Finally, we propose enrolling these two groups concurrently as awaiting the outcome in one subgroup before proceeding with the next would extend the length of the study from 2 years to up to 4 years, unnecessarily delaying scientific progress well beyond 2022.
**NUMBER OF SUBJECTS**	Twenty HIV-infected adults on effective ART, with N=15 having initiated ART during acute infection (within 6 months of estimated infection date) and N=5 having initiated ART during chronic infection (6 months or more after estimated infection date).
**SUBJECT SELECTION CRITERIA**	Main Inclusion Criteria:<br>1. Willing and able to provide written informed consent<br>2. Male, female, or transgender<br>3. Age ≤65 years at the time of enrollment.<br>4. HIV-1 infection, documented by any of the following:<br>a. Licensed rapid HIV test or HIV enzyme or chemiluminescence immunoassay (E/CIA) test kit at any time prior to study entry and confirmed by a licensed Western blot or a second antibody test by a method other than the initial rapid HIV and/or E/CIA<br>b. 4th Generation HIV-1 Ag/Ab test<br>c. HIV-1 antigen or plasma HIV-1 RNA viral load as confirmed by the Principal Investigator<br>d. Research assay result compatible with a diagnosis of HIV infection (e.g. detectable intracellular HIV DNA or viral outgrowth assay) as confirmed by the Principal Investigator<br>5. On continuous antiretroviral therapy for at least 12 months without any interruptions of greater than 14 consecutive days within the last 1 year, and on a stable regimen that does not include an non-nucleoside reverse transcriptase inhibitor (NNRTI) for at least 4 weeks at the time of the first study product administration, without plans to modify ART during the study period<br>6. ART initiated within 6 months of estimated infection date (early ART, n=15) or after six months of their estimated infection date (late ART, n=5). A participant must meet the definition for early infection using an estimated infection date by at least one method: EID, EDDI, or Fiebig staging (I-V) at diagnosis.<br>7. Screening plasma HIV RNA levels below the level of quantification on all available determinations in past 12 months (isolated single values above the level of

	quantification but < 200 copies/mL will be allowed if they were preceded and followed by unquantifiable viral load determinations)<br>8. Screening CD4+ T-cell count ≥ 500 cells/mm^3<br>9. Creatinine Clearance (CrCl) > 60 mL/min via Cockroft-Gault method at screening<br>10. The following laboratory criteria must be met at screening:<br>• Absolute neutrophil count (ANC) ≥ 1000 neutrophils/mm^3<br>• Hemoglobin ≥ 10.0 g/dL<br>• Platelet count ≥ 100,000/uL<br>• Aspartate aminotransferase (AST) ≤ 2x upper limit of normal (ULN)<br>• Alanine aminotransferase (ALT) ≤ 2x ULN<br>• Total and direct ≤ ULN<br><br><u>Main Exclusion Criteria</u>:<br>1. Subjects receiving a non-nucleoside reverse transcriptase inhibitor and unable to change to a different regimen<br>2. Pregnant, breastfeeding, or unwilling to practice birth control (see below) during participation in the study<br>3. Acceptable birth control is defined as the following:<br>a. For female participants of childbearing potential, two of the following forms of contraception are required, one of which must be a barrier method: (1) condoms (male or female) with or without a spermicidal agent, (2) diaphragm or cervical cap with spermicide, (3) intrauterine device (IUD) with published data showing that expected failure rate is < 1% per year, (4) tubal ligation, (5) hormone-based contraceptive such as oral birth control pills<br>b. Male participants participating in sexual activity that could lead to pregnancy must agree to at least one reliable method of contraception of the above listed<br>4. High-level resistance to both 10-1074 and VRC-07 as defined using the PhenoSense Neutralizing Antibody Assay (Monogram Biosciences). The clinical thresholds for defining activity with this or other assays are unknown. We will exclude individuals whose virus susceptibility (IC50) is in the top 10% for at least one of the antibodies or in the top 25% for both antibodies. See Protocol section 9.11 for detail.

	Any history of an HIV-associated malignancy, including Kaposi's sarcoma and any type of lymphoma, or virus-associated cancers.<br>5. Active or recent non-HIV-associated malignancy requiring systemic chemotherapy or surgery in the preceding 36 months or for whom such therapies are expected in the subsequent 12 months; minor surgical removal of localized skin cancers (squamous cell carcinoma, basal cell carcinoma) are not exclusionary<br>6. CD4+ T cell nadir <350 cells/mm^3 during the chronic phase of infection (beginning 6 months following the estimated infection date and confirmed on repeat testing). Individuals with CD4+ T cell values <350 cells/mm^3 within 6 months of the estimated infection date which subsequently normalize will be eligible for inclusion.<br>7. Evidence of HIV "elite" control prior to ART as defined by having HIV RNA levels below the limit of detection without exposure to ART, using commercially available assays at that time<br>8. History of current active hepatitis B (HBV) infection defined as positive HBV surface antigen test.<br>9. Active hepatitis C (HCV) infection. Subjects must be HCV antibody negative or have evidence of cleared HCV infection. If the subject HCV antibody positive, an unquantifiable HCV RNA result (<LLOQ, either target detected or target not detected) within 42 days prior to study entry is required. Those who are currently receiving HCV antiviral therapy or those who have received HCV treatment in the last 6 months prior to study entry will be excluded.<br>10. Chronic liver disease including known compensated cirrhosis or uncompensated cirrhosis, as defined by the presence of ascites, encephalopathy, esophageal or gastric varices, or persistent unexplained jaundice.<br>11. Active and poorly controlled atherosclerotic cardiovascular disease (ASCVD), as defined by 2013 ACC/AHA guidelines, including a previous diagnosis of any of the following: (a) acute myocardial infarction, (b) acute coronary syndromes, (c) stable or unstable angina, (d) coronary or other arterial revascularization, (e) stroke, (f) transient ischemic attack (TIA), or (g) peripheral arterial disease presumed to be of atherosclerotic origin.<br>12. Presence of significant abnormalities on electrocardiogram

	13. History of potential immune-mediated medical conditions (see Appendix A). Individuals with isolated Raynaud's phenomenon or localized disease requiring topical therapy alone will not be excluded.<br>14. Serious illness requiring systemic treatment and/or hospitalization in the 3 months prior to study enrollment<br>15. Concurrent treatment with immunomodulatory drugs, and/or exposure to any immunomodulatory drug in the 4 weeks prior to study enrollment (e.g. corticosteroid therapy equal to or exceeding a dose of 15 mg/day of prednisone for more than 10 days, IL-2, interferon-alpha, methotrexate, cancer chemotherapy). Use of inhaled or nasal steroid is not exclusionary.<br>16. Exposure to any vaccination within 7 days of study enrollment or exposure to any experimental vaccination within 90 days of study enrollment. Study participants will be encouraged to get the influenza vaccine and any other routine vaccinations (including those for hepatitis A, hepatitis B and varicella zoster) prior to screening for the study. Routine influenza or other clinically-required vaccinations during the study period will be allowed.<br>17. Exposure to any experimental therapies within 90 days of study entry. Study subjects will not be allowed to receive other experimental therapies during the course of the study.<br>18. Serious medical or psychiatric illness that, in the opinion of the investigator, would interfere with the ability to adhere to study requirements or to give informed consent.<br>19. Active drug or alcohol use or dependence that, in the opinion of the site investigator, would interfere with adherence to study requirements or to give informed consent.<br>20. Serious medical or psychiatric illness that, in the opinion of the investigator, would interfere with participation in the trial may interfere with interpretation of study results or place the subject at risk for harm or injury.<br>21. Acute or chronic bleeding or clotting disorder that would contraindicate IM injections, or use of blood thinners (e.g. anticoagulants or antiplatelet drugs) within 2 weeks of Day 0<br>22. Less than two acceptable sites available for IM injection considering the deltoid and anterolateral quadriceps muscles

23. Individuals in whom the ability to observe possible local reactions at the eligible injection sites is, in the opinion of the investigator, unacceptably obscured due to a physical condition or permanent body art, or who have keloids or hypertrophic scars located within 2 cm of intended treatment site.

24. Implanted electronic medical devices (e.g., cochlear implant, pacemaker, implantable cardioverter defibrillator); ii) sinus bradycardia (defined as < 50 beats per minute on exam); or iii) history of cardiac arrhythmia (e.g., supraventricular tachycardia, atrial fibrillation, or frequent ectopy). Please note, sinus arrhythmia is not excluded.

25. Metal implants or implantable medical device within the intended treatment site (i.e. electroporation area)

26. ECG with clinically significant findings, or features that would interfere with the assessment of myo/pericarditis, including any of the following:(1) conduction disturbance (complete left or complete right bundle branch block or nonspecific intraventricular conduction disturbance with QRS $\geq$ 120 ms, PR interval $\geq$ 220ms, any 2nd or 3rd degree AV block, or QTc prolongation ($>$ 450ms)); (2) repolarization (ST segment or T wave) abnormality that will interfere with the assessment of myo/pericarditis; (3) significant atrial or ventricular arrhythmia; (4) frequent atrial or ventricular ectopy (eg, frequent premature atrial contractions, 2 premature ventricular contractions in a row); (5) ST elevation consistent with ischemia; (6) evidence of past or evolving myocardial infarction. Serious medical or psychiatric illness that, in the opinion of the site investigator, would interfere with the ability to provide informed consent, adhere to study requirements, affect interpretation of the study results or place subject at risk for harm, e.g., a history of myo/pericarditis."

27. Known allergy/sensitivity or any hypersensitivity to components of study drug or their formulation, e.g., egg allergy (inability to consume eggs or baked goods containing eggs), allergy to any amide-type local anesthetic (such as bupivacaine (Marcaine®), lidocaine (Xylocaine®), mepivacaine (Polocaine® Carbocaine®), etidocaine (Duranest®), or prilocaine (Citanest®), EMLA® cream.

	28. Individuals in whom a skin-fold measurement of the cutaneous and subcutaneous tissue for eligible injection sites exceeds 40 mm.<br>29. History of severe allergic reactions (e.g., angioedema, anaphylaxis) or severe reactions to prior vaccination (e.g., severe injection site reaction, anaphylaxis).<br>30. Chronic or active neurologic disorder which, in the opinion of the investigator, would place the participant at risk of harm or injury. Note that individuals with stable headaches, stable peripheral neuropathy, and stable post-herpetic neuralgia may be included.<br>31. Confirmed syncopal episode determined or suspected to be of cardiac origin within 12 months of screening.<br>32. Significant or progressive ophthalmologic disease, including significant signs or symptoms of active disease.
**TEST PRODUCT, DOSE, AND ROUTE OF ADMINISTRATION**	• HIV DNA vaccine (p24CE and p55gag) plasmids<br>• IL-12 DNA plasmid<br>• MVA/HIV62B (MVA62B) vaccine<br>• TLR9 agonist (lefitolimod, MGN1703)<br>• bNAb (VRC07-523LS and 10-1074)
**DURATION OF SUBJECT PARTICIPATION AND DURATION OF STUDY**	Subjects will be observed on study for up to 112 weeks.
**CONCOMITANT MEDICATIONS**	All subjects should be maintained on a stable antiretroviral drug regimen through the end of Stage 4 and then interrupt therapy.
**PRIMARY ENDPOINTS**	• Proportion of individuals experiencing a grade 3 or greater adverse event, including signs/symptoms, lab toxicity, or clinical event, that is definitely, probably, or possibly related to the study treatment.<br>• Proportion of treated individuals who achieve post-treatment control (PTC) of viral load, defined as having an undetectable viral load (<50 copies RNA/mL) at 24 weeks after initial rebound in viremia for those who rebound or through week 24 of the interruption for those who do not exhibit a rebound
**SECONDARY ENDPOINTS**	• Number of unsolicited adverse events within 28 days after administration of each study agent.<br>• Number of any severe adverse event, medically attended adverse event, or potentially immune-mediated medical

	condition from the time of administration of the first study injection through 12 months after administration of the final study injection.<br>• Occurrence of two consecutive measurements of HIV RNA >200 copies/mL using conventional clinical assays.<br>• Number, magnitude, polyfunctionality, and cytotoxic phenotype of CE-targeted T cell responses elicited by the vaccination and maintained in the peripheral blood throughout the study as measured by intracellular cytokine staining (ICS) after stimulation with CE-derived peptides. Responses to Gag, Pol, Env, and Nef overlapping peptide pools will be evaluated in parallel<br>• Frequency of circulating CD4+ T cells harboring replication-competent HIV as measured using multiplex digital droplet PCR assay to quantify the *total* number of intact proviruses
**OTHER EVALUATIONS**	• Phenotypic characteristics (e.g., expression of PD-1, TIGIT, CD160, 2B4, Tbet, TCF-1) of *de novo* vaccine-induced (CE-specific) HIV-specific CD8+ T cell populations (as detected using MHC class I-specific tetramers) compared to longitudinally-studied pre-existing HIV-specific CD8+ T cell populations<br>• Phenotypic characteristics (e.g., expression of CD69, Ki-67) of innate immune cells using mass cytometry (CyTOF)<br>• Transcriptomic analysis of FACS-sorted tetramer-detectable HIV-specific CD8+ T cell populations using RNA-Seq<br>• Transcriptomic analysis of longitudinally-collected whole blood samples<br>• Functional features of HIV-specific antibodies (ADCC, phagocytosis, complement-mediated destruction, neutrophil activation, and dendritic cell uptake)<br>• Plasma HIV RNA (single copy assay), cell-associated RNA, cell-associated DNA, Tat/Rev Inducible HIV RNA Assay (TILDA)
**SAFETY EVALUATIONS**	All subjects will be followed for possible adverse events (AEs) throughout their involvement in the study. Routine blood work will be performed on a regular basis. AEs will be graded according to Version 2.1 (July 2017) of the NIH/NIAID Division of AIDS Table for Grading the Severity of Adult and Pediatric Adverse Events.
**EFFICACY EVALUATIONS**	All subjects will undergo a treatment interruption designed to evaluate the primary outcome of "post-treatment control" (PTC). The proportion

	of individuals who achieve PTC will be tabulated and a 2-sided 95% confidence interval obtained by exact calculations for the binomial distribution. The proportion will also be compared to historical controls, with a P-value obtained by likelihood ratio test. For those who initiated ART during acute infection, a literature review indicates that no more than 15% PTC would be expected without intervention. For those who initiated during chronic infection, no more than 5% PTC would be expected. These maximum expected rates will serve as the null hypothesis for our P-value calculations. Evaluation of the acute group will be the primary analysis, while evaluation of the chronic group will be exploratory and provide initial preliminary information on generalizability.<br><br>Using standard and innovative measures of immunogenicity, we will determine the capacity of our strategies to stimulate broad, functional T cell responses against conserved element HIV epitopes, and comprehensively characterize the impact of the vaccine on several innate and adaptive immune parameters. The size of the active and latent reservoirs before, during and after vaccination will be measured.
**SAMPLE SIZE JUSTIFICATION**	For the acute group, observing 6 or more PTC among N=15 (40%) will result in $P<0.05$ versus the null hypothesis of 15%, and the study will have ≥80% power if the true chance of PTC with intervention is ≥47%.<br><br>For the chronic group, observing even one PTC (20%) would provide some indication of promise, and observing 2 PTC (40%) would produce $P<0.05$ versus the null hypothesis of 5%.
**PLANNED INTERIM ANALYSES**	A Safety Monitoring Committee (SMC) will be convened and will meet biannually to review all adverse events and the conduct of the study. Serious adverse events will be monitored by the committee on an ongoing basis throughout the study.

TABLE OF CONTENTS

1 BACKGROUND 23
1.1 Study background and rationale....23
1.1.1 CD8+ T cell immunity in a therapeutic setting 23
1.1.2 Prime-boost vaccine regimen followed by a TLR7 agonists....24
1.1.3 TLR7 agonists during ART can induce an SIV cure/remission....24
1.1.4 Emerging role of ADCC responses in a therapeutic setting 25
1.1.5 TLR7 agonists and bNAbs induce an SIV cure/remission 25
1.1.6 The impact of bNAbs on CTL when administered during acute viremia....26
1.1.7 Summary....26
1.2 Description of Products and Previous Human Use 27
1.3 Product Dosing....29
1.4 Overview of p24CE and p55gag DNA Vaccines....31
1.4.1 CE vaccine overview 31
1.4.2 p24CE1/2 pDNA vaccine 32
1.4.3 Immunogenicity of p24CE pDNA vaccine in macaques....34
1.4.4 Immune responses induced by HIV CE and HIV gag pDNA vaccines....34
1.4.5 Development of DNA prime-boost regimens....35
1.4.6 MVA62B boost of CE vaccinated macaques 37
1.4.7 Immunogenicity of the CE pDNA vaccine regimen during ART 39
1.4.8 Durability of HIV CE-specific immune responses....40
1.4.9 Humoral responses to p24CE pDNA and p55gag pDNA vaccination 40
1.4.10 Overall Nonclinical Toxicology Summary....40
1.5 Overview of the IL-12 DNA plasmids....41
1.5.1 IL-12 DNA as DNA vaccine adjuvant....41
1.5.2 Safety of *IL-12* pDNA in Clinical Trials 41
1.5.3 HVTN 060 and 063: Use of p37gag pDNA and IL-12 pDNA....46
1.5.4 HVTN 070 and 080: Use of $p55^{gag}$ pDNA, *IL-12* pDNA and EP 48
1.5.5 HVTN 087: pIL-12 as an adjuvant for DNA prime/VSV boost strategy 51
1.6 Overview of the Ichor TriGrid Delivery System (TDS-IM v1.0)....52
1.7 Overview of MVA62B 53
1.7.1 MVA62B in homologous and heterologous prime/boost regimens....53
1.7.2 Safety and tolerability in HIV-uninfected individuals....53
1.7.3 Safety and Immunogenicity in HIV-uninfected adults....54
1.7.4 MVA62B in a therapeutic setting (GV-TH-01)....55
1.7.5 Safety issues 56
1.8 Overview of lefitolimod (MGN1703)....56

1.8.1 Mechanism of action 57
1.8.2 Pharmacology of lefitolimod 57
1.8.3 Immunology 59
1.8.4 Phase I study of lefitolimod in colorectal cancer 59
1.8.5 Lefitolimod in HIV Disease 60
1.8.6 Lefitolimod may induce HIV production and reduce the reservoir size 63
1.9 Overview of 10-1074 64
1.9.1 Physical, Chemical and Pharmaceutical Properties of 10-1074 64
1.9.2 Nonclinical Pharmacology 64
1.9.3 Non-clinical Toxicology 65
1.9.4 Clinical Development 66
1.9.5 Clinical experience with 10-1074 in combination with other antibodies 66
1.9.6 Mechanism of action 67
1.9.7 Summary of Preclinical and Clinical Data with 10-1074 67
1.9.8 Dose and administration of 10-1074 67
1.10 Overview of VRC07-523LS 67
1.10.1 Potency and efficacy of VRC07 in preclinical studies 68
1.10.2 Pre-clinical toxicity studies of VRC07 68
1.10.3 Clinical experience with VRC07 68
1.10.4 Pharmacokinetic Parameters of VRC07-523LS in Adults 69
1.11 Analytic treatment interruptions 70
2 STUDY RATIONALE 77
2.1 Study Rationale and Hypothesis 77
2.2 Risk / Benefit Assessment 78
2.3 Program Overview 78
3 STUDY OBJECTIVES 80
3.1 Primary Objectives 80
3.2 Secondary Objective 80
4 STUDY DESIGN 81
4.1 Study Overview 81
5 CRITERIA FOR EVALUATION 82
5.1 Primary Endpoints 82
5.2 Secondary Endpoint 82
5.3 Other Endpoints 82
6 SUBJECT SELECTION 83
6.1 Study Population 83
6.2 Inclusion Criteria 85
6.3 Exclusion Criteria 86
7 CONCURRENT MEDICATIONS 90
8 STUDY TREATMENTS 91
8.1 Product management 91

8.2 HIV DNA Expression Plasmids....91
8.3 GENEVAX® IL-12 pDNA Vaccine Adjuvant....95
8.4 TDS-IM v1.0 Electroporation Device....97
8.5 MVA62B....100
8.6 10-1074....101
8.7 VRC07-523LS....101
8.8 Lefitolimod....102
9 STUDY PROCEDURES 104
9.1 Concomitant Medications....104
9.2 Demographics and Medical History....104
9.3 Clinical Assessment....104
9.4 Physical Examination....105
9.5 Clinical Laboratory Measurements....105
9.6 HIV Clinical Laboratory Measures....106
9.7 Cryopreservation of Plasma....106
9.8 Cryopreservation of PBMCs....106
9.9 Collection of PAXgene specimen....106
9.10 Pharmacokinetic Measurements....106
9.11 PhenoSense Neutralizing Antibody Assay....106
9.12 Cellular Immunologic Measurements....107
9.13 Transcriptomic Measurements....108
9.14 Antibody Measurements....109
9.15 Virologic Measures....109
9.16 Sample processing and specimen management....109
10 EVALUATIONS BY VISIT 110
10.1 Overview....110
10.2 Procedures: Staggered enrollment....110
10.3 Screening and baseline evaluations....110
10.4 p24CE and IL-12 DNA vaccinations (Stage 1)....111
10.5 p24CE, p55^{gag} and IL-12 DNA vaccinations (Stage 2)....112
10.6 MVA vaccination (Stage 3)....112
10.7 Combination bNAbs and lefitolimod administrations (Stage 4)....113
10.8 Combination bNAbs and treatment interruption phase (Stage 5)....113
10.9 ART resumption phase (Stage 6)....114
10.10 End of study....115
10.11 Premature discontinuation....115
10.12 Schedule of events....116
11 EXPERIENCE REPORTING AND DOCUMENTATION 122
11.1 Adverse Events....122
11.2 AE Severity....122
11.3 AE Relationship to Study Drug....123

11.4 Serious Adverse Events (SAE) 123
11.5 Serious Adverse Event Reporting 123
11.6 Unanticipated (Serious) Adverse Device Effect (UADE) 124
11.7 Medically attended adverse events 124
11.8 Local reactogenicity 124
11.9 Acute Retroviral Syndrome 127
11.10 Procedures for Documenting Pregnancy During the Trial 128
11.11 Reporting of Device Related Complaints or Deficiencies 128
12 CLINICAL MANAGEMENT ISSUES 130
13 DISCONTINUATION AND REPLACEMENT OF SUBJECTS 131
13.1 Early Discontinuation 131
13.2 Withdrawal of Subjects from the Study 131
13.3 Replacement of Subjects 131
14 PROTOCOL VIOLATIONS 132
15 STATISTICAL METHODS AND CONSIDERATIONS 133
15.1 Data Sets Analyzed 133
15.2 Safety Analysis 133
15.3 Safety Endpoints 133
15.4 Primary Efficacy Endpoint: Post-treatment Control 134
15.5 Secondary Other Efficacy Endpoints 135
15.6 Sample Size 136
16 DATA COLLECTION, RETENTION AND MONITORING 138
16.1 Clinical trial infrastructure 138
16.2 Data Collection Instruments 139
16.3 Research Electronic Data Capture (REDcap) 139
16.4 Biologic specimen collection, processing and storage 139
16.5 Data Management Procedures 141
16.6 Data Quality Control and Reporting 142
16.7 Archival of Data 142
16.8 Availability and Retention of Investigational Records 142
16.9 Monitoring 142
16.10 Safety Monitoring Committee (SMC) 143
16.11 Subject Confidentiality 144
17 ADMINISTRATIVE, ETHICAL, REGULATORY CONSIDERATIONS 145
17.1 Protocol Amendments 145
17.2 Institutional Review Boards and Independent Ethics Committees 145
17.3 Informed Consent Form 146
17.4 Publications 146
17.5 Investigator Responsibilities 146

18 REFERENCES 148
19 **CONSENT TO PARTICIPATE IN A RESEARCH STUDY 159**
19.1 What experimental medications will be used? 160
20 APPENDIX A: POTENTIAL IMMUNE-MEDIATED MEDICAL CONDITIONS 177
21 APPENDIX B: PROTOCOL UPDATES 180

1 BACKGROUND

Although antiretroviral therapy (ART) decreases HIV-associated mortality, it does not appear to completely restore health, for reasons that remain unclear. In addition, while prevention approaches have seen some significant successes in the past few years, the epidemic continues to grow both locally and globally. Perhaps the only way to fully address these and other limitations is to effectively eradicate HIV from infected persons. While complete eradication may never be feasible, a “functional cure” in which patients are able to maintain undetectable viral loads indefinitely in the absence of therapy may be possible. The best evidence for this is the existence of “elite controllers” and more recently individuals treated during very early HIV infection (“post-treatment controllers”).

Our program is based on the premise that durable remission in HIV disease will require generating a potent and sustained virus-specific CD8+ T cell response that is able to continuously clear and/or suppress virus-producing cells that arise from reactivation of the latent pool. Previous efforts to boost CD8+ T cell immunity in a therapeutic setting have largely failed, in part because the effector responses in vaccinated individuals were weak, transient, and targeted immunodominant epitopes that often have already escaped[3-5]. Given the dissemination of the viral reservoir and the rapid rate of virus spread during a treatment interruption, it is likely that the virus overwhelmed any vaccine-mediated T cell responses.

Historical efforts aimed at “auto-vaccinating” HIV infected individuals with their own virus via scheduled treatment interruptions, aimed at priming more effective immunity and ultimate control of viremia, resulted in relatively disappointing results[6]. This failure is likely due to three major barriers. First, pre-existing immune exhaustion within the HIV-specific CD8+ T cell population may have hampered the induction of *de novo* highly effective responses. Second, exposure to autologous virus may have simply recalled immunity to highly immunodominant responses that had already failed due to the selection for escape mutations. Third, the number of effector cells able to contain a robust rebounding virus population may have been insufficient, thus allowing the virus to outpace the immune response (“too little, too late”).

1.1 Study background and rationale

It is generally accepted that combination approaches will be required to generate durable control of HIV in the absence of antiretroviral therapy (a “remission”).

1.1.1 CD8+ T cell immunity in a therapeutic setting

Based on studies in “elite” controllers and other chronic virus infections, durable control of HIV will likely require potent and sustained HIV-specific CD8+ T cells that target conserved epitopes. Multiple groups have over the past two decades attempted to use a therapeutic vaccine to generate an effective HIV-specific T cell response in HIV-infected adults on effective antiretroviral. These vaccines have generally been safe, well-tolerated and safe, but none has shown any consistent effect on the reservoir or on the viral load “set-point” after antiretroviral therapy was discontinued. Multiple factors likely account for these failures, including the concern that these vaccines generate weak responses to immunodominant responses that already failed to control the infection. Ideally, future efforts would seek to generate more potent and sustained responses that target conserved areas of the virus. Specifically, a vaccine designed to shift responses from the highly variable immunodominant epitopes that typically dominate the immune response to HIV towards more conserved areas might prove effective. In this study, we will prime the immune response

with a DNA plasmid vaccine designed to generate subdominant responses to conserved regions within Gag (the "p24 Conserved Element", or p24CE vaccine). In our pre-clinical non-human primate studies, we have demonstrated impressive immunogenicity with this vaccine, particularly with the initial response is boosted with a heterologous viral vector (MVA)[7-9].

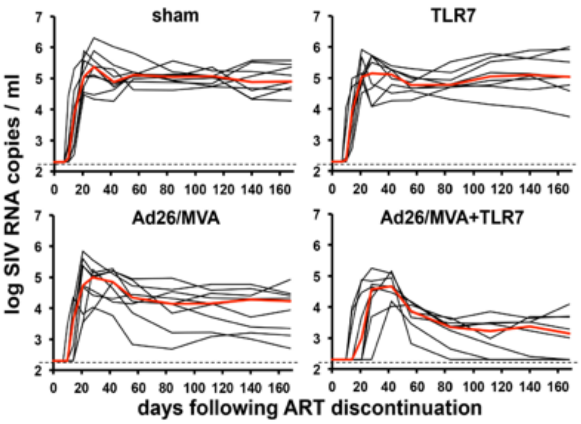

1.1.2 Prime-boost vaccine regimen followed by a TLR7 agonists

It is unlikely, however, that a prime/boost vaccine will prove sufficient, particularly in chronic infection in which immune responses tend to be blunted. We will hence further boost the vaccine effectiveness by administering a TLR9 agonist after the prime/boost phase. Our rationale is based in large part on a study performed by Barouch and colleagues[10]. In this study of 40 SIV-infected macaques on effective ART, animals were assigned to receive (during ART) either sham, a TLR7 agonist (GS-9620, given orally), an Ad26 prime/MVA boost vaccine, or an Ad26/MVA vaccine followed by repeated doses of a TLR7 agonist. In the combination arm, Ad26 was administered twice (Weeks 24 and 36 of ART) followed by the first MVA boost (Week 48 of ART). During peak immunity (two weeks after first MVA administration) the TLR7 agonist (GS-9860) was initiated and given every two weeks for 10 doses (Weeks 50 to 70). The second MVA dose was given at Week 60.

The rationale for administering the TLR7 agonist after the prime/boost regimen was based on several factors, including (1) need to avoid stimulating an type I interferon-mediated inhibitory effect that may reduce the effectiveness of MVA, (2) to boost any memory responses that generated two weeks after MVA (the predicted peak of the effector response) during latency reversal to generate a "shock and kill" effect and (3) to address any potential regulatory concerns associated with co-administration of a vaccine and a potential vaccine adjuvant.

The use of the Ad26/MVA/TLR7 agonist was associated with a more effective reduction of the reservoir during ART and a sustained T cell response after ART was discontinued. This effect of reservoir reduction and sustained T cell responses resulted in improved control of SIV after ART was discontinued (see Figure). Consistent with this model, the total breadth of the responses to Gag, Pol and/or Env prior to ART interruption was associated with post-interruption viral load setpoint.

In our proposed study, we will follow our DNA/MVA prime/boost regimen with a TLR9 agonist that has already been shown to be safe and active in HIV-infected adults.

1.1.3 TLR7 agonists during ART can induce an SIV cure/remission

In a recent publication by Whitney and colleagues, two studies of Gilead's TLR7 agonists (GS-986 or GS-9620, both administered orally) in antiretroviral drug treated SIV-infected macaques were performed[11]. In each study, TLR7 agonists caused reproducible increases in NK and T cell activation. This inflammatory effect appeared to cause latency reversal and a dramatic reduction in the reservoir size, leading to a delayed rebound after ART was interrupted. Remarkably, two

animals had no detectable virus during adoptive transfer experiments 448 days after treatment was interrupted, suggesting a complete cure had been achieved.

1.1.4 Emerging role of ADCC responses in a therapeutic setting

Although the vast majority of studies have focused on enhancing CD8+ T cell responses, it is likely that other components of the host immune response (particularly ADCC and other antibody-mediated effector responses) might be necessary to generate a response. Antibody therapy in combination with adjuvants that enhance cytotoxicity might be necessary to achieve the type of response needed to durably control HIV in the absence of therapy.

Recent data from studies of elite and post-treatment controllers suggest that clearance of HIV during ART or control of HIV after ART may require potent NK cell responses, in addition to potent CD8+ T cell responses. Env-specific antibodies capable of stimulating antibody-dependent cellular cytotoxicity (ADCC) by recruiting NK cells and other effector responses may also be critical, suggesting that any effective therapeutic curative study will need to enhance such responses. A common approach to enhance these responses is to co-administer an immunomodulatory (such as TLR9 agonist) with bNAbs, hence generating a potent NK cell response and the capacity for ADCC.

1.1.5 TLR7 agonists and bNAbs induce an SIV cure/remission

In a recently presented but unpublished study, Barouch and colleagues studied the effect of PGT121, a bNAb that targets V3 glycan, and a TLR7 agonist GS9620, in SHIV-infected rhesus monkeys who were started on ART during "hyperacute" infection (day 7 of their infection). After nearly 2 years of effective ART, the animals were treated with either (1) PGT121 every 2 weeks for 5 doses, (2) GS-9620 every two weeks for 10 doses, (3) both PGT121 and GS-9620, or (4) sham controls. In the combination arm, GS-9620 was given alone for five doses. PGT121 was then started and five more dose of GS-9620 were administered (the antibody was timed such that therapeutic levels would be achieved during the periods of optimal GS-9620-mediated latency reversal). Sixteen weeks after the last antibody infusion (when no more antibody was detected), ART was interrupted. The reservoir was low in all animals at the time of PGT121 and GS9620 were started.

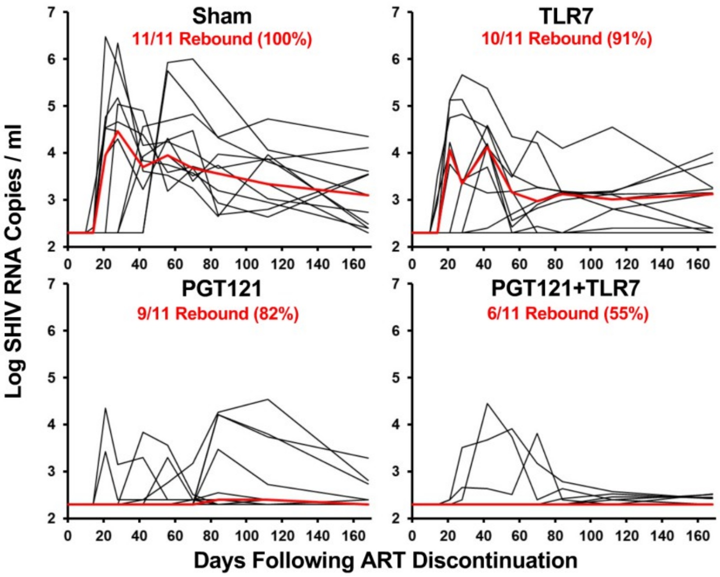

These interventions had no consistent effect on SIV-specific CD8+ T cell responses but PGT121 alone and the combination of the two interventions caused an apparent clearance of all detectable virus in some animals. After ART was interrupted, virus rebounded rapidly in the control arm. There was a modest delay and lower set-point in those receiving just GS-9620 or PGT121. In contrast, virus rebound was not detected in approximately half (5 of 11) of the animals receiving the combination. Of those exhibiting a rebound, the level of viremia

was low and eventually became low or undetectable (<400 copies RNA/mL).

These data suggest that the combination of a bNAb and a TLR7 agonist during ART reduces the reservoir size through unknown reasons, although a "shock and kill" effect is assumed. This same combination also induces a sustained host environment that causes virus control in absence of ART.

1.1.6 The impact of bNAbs on CTL when administered during acute viremia

Recent data from the SIV non-human primate model suggests that bNAbs can alter the immune response in treated animals undergoing a treatment interruption. In a model of macaque simian/human immunodeficiency virus (SHIV) infection, the administration of a combination of bNAbs (3BNC117 and 10-1074) during the early acute phase lead to potent but apparently incomplete control of SIV infection [12]. Residual low-level viremia persisted during bNAb therapy. As the bNAb levels waned, virus rebounded transiently. This was followed by a prolonged period of sustained virus control in the absence of any additional therapy. Antibody-mediated depletion of CD8+ T cells lead to virus rebound, suggesting that bNAb therapy generated a potent adaptive immune response, possibly as a consequence of the immune complexes (antibody and antigen) that formed. These complexes have the potential to stimulate dendritic cells and other antigen-presenting cells.

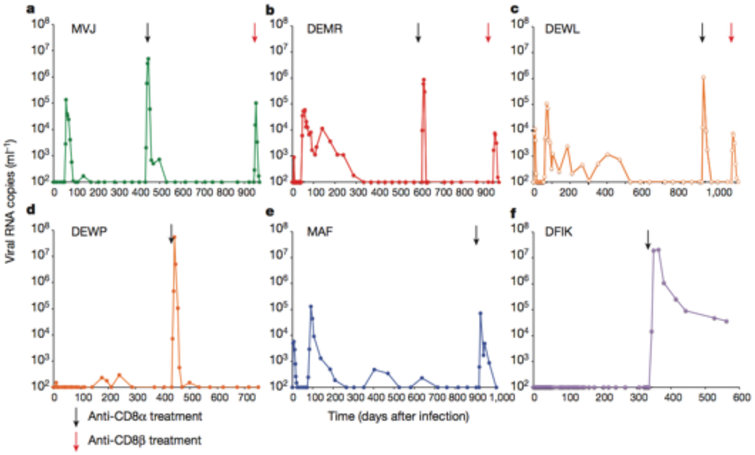

Of note, treatment of the SHIV-infected macaques with ART for a period of time comparable to the bNAb therapy did not result in durable post-treatment control, providing additional evidence for an immunomodulatory effect of bNAbs independent of direct virus suppression.

1.1.7 Summary

Based on these studies, we predict that sequential exposure to (a) a highly immunogenic heterologous prime/boost vaccine (DNA/MVA) vaccine, (b) a combination of bNAbs and a TLR9 agonist and (c) bNAb administration during a treatment interruption will reduce the reservoir size (during ART) and generate a robust host response that results sustained control of HIV replication in absence of therapy.

1.2 Description of Products and Previous Human Use

Product Name	IND Number	Product Description	Previous Use
**p24CE1/2 plasmid DNA**	BB-IND 17457 held by Division of AIDS, NIAID	A multivalent HIV-1 M group conserved element (CE) plasmid DNA vaccine encoding selected sequences from the human immunodeficiency virus antigen, p24gag protein	Currently being evaluated in a phase 1 study in HIV-uninfected volunteers to evaluate safety and immunogenicity
**p55^{gag} plasmid DNA**		A pDNA vaccine encoding the full-length p55gag sequence	
**IL-12 DNA plasmid (GENEVAX® IL-12 pDNA)**	IND BB-MF 11998 held by Profectus Biosciences	A dual promoter expression plasmid that expresses the two genes encoding human IL-12 subunits p35 and p40 under separate regulatory control	Multiple phase 1 and phase 2 studies have performed without any safety concerns
**MVA/HIV62B (MVA62B)**	BB-IND 12930 held by Division of AIDS<br><br>BB-IND 12161 held by GeoVax	Double recombinant Modified Vaccinia Ankara expressing HIV-1 *gag, pol* and *env* genes.	Multiple phase 1 and phase 2 studies that evaluated a DNA prime, MVA boost regimen
**10-1074**<br>**(unmodified non-LS version)**	IND 123713 held by Rockefeller University	10-1074 is a monoclonal antibody (IgG1λ isotype) that targets the base of the V3 loop.	10-1074 has been studied alone in uninfected and infected subjects (Caskey, Nat Med 2017) and is now being studied in combination with a second antibody (NCT02824536).
**VRC07-523LS (VRC07)**	IND 1330027 held by VRC and NIAID/NIH	VRC07-523LS is a monoclonal antibody that targets the CD4-binding site on the HIV envelope spike	VRC07-523LS has been tested in HIV-uninfected subjects only; the antibody is similar to VRC01 and VRC07, both shown to be safe and effective in HIV-infected adults

**Lefitolimod (MGN1703)**	IND 104781 held by Mologen	Lefitolimod or MGN1703 is a synthetic DNA-based immunomodulatory molecule with two single-stranded loops separated by a double-stranded stem. The 116-nucleotide dumbbell-shaped is enriched for immunomodulatory GC-motifs (similar to CpG motifs).	Lefitolimod has been studied in hundreds of individuals with cancer and is now in phase III testing for colorectal cancer. Lefitolimod has undergone phase I/II assessments in HIV-infected adults on ART (n=15).

1.3 Product Dosing

Product Name	Dose	Administration route	Frequency	Comment
**p24CE1/2 plasmid DNA**	2-4 mg (see comment)	IM injection by electroporation (TriGrid Delivery System v1.0)	Weeks 0, 4 and 12	Week 0:<br>• 2mg in injection site #1<br>• 2mg in injection site #2<br>Week 4:<br>• 2mg in injection site #1<br>• 2mg in injection site #2<br>•<br>Week 12:<br>• 2mg in injection site #1<br>2mg in injection site #2
**p55^{gag} plasmid DNA**	2mg (see comment	IM injection by electroporation (TriGrid Delivery System v1.0)	Week 12	Week 12:<br>• 1mg in injection site #1<br>1mg in in injection site #2
**IL-12 plasmid DNA**	2mg (see comment)	IM injection by electroporation (TriGrid Delivery System v1.0)	Weeks 0, 4 and 12	Week 0:<br>• 1mg in injection site #1<br>• 1mg in in injection site #2<br>Week 4:<br>• 1mg in injection site #1<br>• 1mg in in injection site #2<br>Week 12:<br>• 1mg in injection site #1

				1mg in in injection site #2
**MVA/HIV62B (MVA62B)**	10^8 TCID50	IM injection (deltoid)	Week 20	MVA boosting typically scheduled for 8 weeks after last DNA prime
**10-1074**	30 mg/kg	Intravenous infusion	Weeks 24 and 34	Half-life of 20-23 days; therapeutic levels (> 10 ug/mL) expected for at least 8 weeks; pre-ATI infusion to occur two days before ART is interrupted; 10-1074 will be administered first, the line will be flushed, and then VRC07-532LS will be administered
**VRC07-523LS (VRC07)**	20 mg/kg	Intravenous infusion	Weeks 24 and 34	Formal PK analysis pending; therapeutic levels (> 10 ug/mL) expected for at least 12 to 16 weeks; pre-ATI infusion to occur two days before ART is interrupted; 10-1074 will be administered first, the line will be flushed, and then VRC07-532LS will be administered
**Lefitolimod (MGN1703)**	60 mg	Subcutaneous injections	9 weekly doses (weeks 25-33)	60 mg subcutaneously once weekly, administered subcutaneously as two 2-mL bilateral injections<br><br>As of Protocol Version 2.2, no further lefitolimod will be administered

1.4 Overview of p24CE and p55gag DNA Vaccines

1.4.1 CE vaccine overview

[redacted]

[redacted]

[redacted]

[redacted]

1.4.2 p24CE1/2 pDNA vaccine

[redacted]

[redacted]

[redacted]

- [redacted]
- [redacted]
- [redacted]
- [redacted]
- [redacted]
- [redacted]

[redacted]

[redacted]

[redacted]

[redacted]

[redacted]

[redacted]

1.4.3 Immunogenicity of p24CE pDNA vaccine in macaques

[redacted]

1.4.4 Immune responses induced by HIV CE and HIV gag pDNA vaccines

[redacted]

[redacted]

1.4.5 Development of DNA prime-boost regimens

[redacted]

[redacted]

[redacted]

[redacted]

[redacted]

[redacted]

[redacted]

[redacted]

1.4.6 MVA62B boost of CE vaccinated macaques

[redacted]

[redacted]

[redacted]

[redacted]

[redacted]

[redacted]

[redacted]

1.4.7 Immunogenicity of the CE pDNA vaccine regimen during ART

[redacted]

[redacted]

[redacted]

[redacted]

[redacted]

[redacted]

1.4.8 Durability of HIV CE-specific immune responses

[redacted]

1.4.9 Humoral responses to p24CE pDNA and p55gag pDNA vaccination

[redacted]

1.4.10 Overall Nonclinical Toxicology Summary

[redacted]

[redacted]

1.5 Overview of the IL-12 DNA plasmids

1.5.1 IL-12 DNA as DNA vaccine adjuvant

[redacted]

[redacted]

[redacted]

1.5.2 Safety of *IL-12* pDNA in Clinical Trials

[redacted]

[redacted]

[redacted]

[redacted]

[redacted]

[redacted]

1.5.3 HVTN 060 and 063: Use of p37gag pDNA and IL-12 pDNA

[redacted]

[redacted]

[redacted]

[redacted]

[redacted]

[redacted]

1.5.4 HVTN 070 and 080: Use of p55^{gag} pDNA, *IL-12* pDNA and EP

[redacted]

[redacted]

[redacted]

[redacted]

[redacted]

[redacted]

[redacted]

[redacted]

[redacted]

[redacted]

[redacted]

[redacted]

[redacted]

1.5.5 HVTN 087: pIL-12 as an adjuvant for DNA prime/VSV boost strategy

[redacted]

[redacted]

[redacted]

[redacted]

[redacted]

[redacted]

[redacted]

1.6 Overview of the Ichor TriGrid Delivery System (TDS-IM v1.0)

[redacted]

[redacted]

[redacted]

[redacted]

[redacted]

[redacted]

[redacted]

1.7 Overview of MVA62B

[redacted]

1.7.1 MVA62B in homologous and heterologous prime/boost regimens

[redacted]

1.7.2 Safety and tolerability in HIV-uninfected individuals

[redacted]

[redacted]

[redacted]

[redacted]

[redacted]

[redacted]

1.7.3 Safety and Immunogenicity in HIV-uninfected adults

[redacted]

[redacted]

1.7.4 MVA62B in a therapeutic setting (GV-TH-01)

[redacted]

[redacted]

1.7.5 Safety issues

[redacted]

[redacted]

1.8 Overview of lefitolimod (MGN1703)

[redacted]

[redacted]

[redacted]

[redacted]

1.8.1 Mechanism of action

[redacted]

[redacted]

1.8.2 Pharmacology of lefitolimod

[redacted]

[redacted]

[redacted]

[redacted]

[redacted]

1.8.3 Immunology

[redacted]

1.8.4 Phase I study of lefitolimod in colorectal cancer

[redacted]

[redacted]

[redacted]

1.8.5 Lefitolimod in HIV Disease

[redacted]

[redacted]

[redacted]

[redacted]

[redacted]

[redacted]

[redacted]

[redacted]

[redacted]

1.8.6 Lefitolimod may induce HIV production and reduce the reservoir size

[redacted]

[redacted]

[redacted]

[redacted]

1.9 Overview of 10-1074

[redacted]

1.9.1 Physical, Chemical and Pharmaceutical Properties of 10-1074

[redacted]

[redacted]

1.9.2 Nonclinical Pharmacology

[redacted]

[redacted].

[redacted]

1.9.3 Non-clinical Toxicology

[redacted]

[redacted]

1.9.4 Clinical Development

[redacted]

[redacted]

[redacted]

[redacted]

[redacted]

1.9.5 Clinical experience with 10-1074 in combination with other antibodies

[redacted] n [redacted]

[redacted]

[redacted]

1.9.6 Mechanism of action

[redacted]

1.9.7 Summary of Preclinical and Clinical Data with 10-1074

[redacted]

1.9.8 Dose and administration of 10-1074

[redacted]

1.10 Overview of VRC07-523LS

[redacted]

[redacted]

[redacted]

[redacted]

1.10.1 Potency and efficacy of VRC07 in preclinical studies

[redacted]

1.10.2 Pre-clinical toxicity studies of VRC07

[redacted]

1.10.3 Clinical experience with VRC07

[redacted]

[redacted]

1.10.4 Pharmacokinetic Parameters of VRC07-523LS in Adults

[redacted]

[redacted]

1.11 Analytic treatment interruptions

There are no viable biomarkers for the size of the reservoir. Current virologic assays either do not measure the relevant reservoir (e.g., HIV DNA levels) or are laborious, have limited range, and lack validation (e.g., the virus outgrowth assay). Similarly, the immunologic correlates that predict control of HIV in absence of therapy are unknown, in part because we do not know the correlates of post-treatment control.

Given the lack of a valid surrogate marker of cure research the only real option is to turn to clinical outcomes. Since the goal of any curative intervention is to extend the degree of ART-free virus suppression, the inconvenient truth is that there is really is no viable way to truly define how a drug works other than to interrupt therapy and measure the outcome. Interrupting ART remains the only "gold-standard" test of a cure or remission.

We are aware that interrupting ART has potential for significant risks. We will use a conservative study design to minimize risk. All individuals will be on a regimen that includes antiretroviral drugs with rapid clearance. This will minimize the chance that drugs will persist long enough to select for drug resistance in the rebounding virus. We will only include individuals with high CD4+ T cell counts to minimize the risk of developing clinically relevant immunodeficiency once the virus rebounds. We will monitor individuals during the study, and resume therapy for a persistently high viral load, low CD4+ T cell count or the development of symptomatic disease.

Individuals who interrupt drugs that persist for days to weeks are at the risk of developing a drug resistant variant. This risk is particularly relevant when interrupting non-nucleoside reverse transcriptase inhibitors (NNRTIs), as these drugs often have very long half-lives and as resistance to these drugs is easy to generate. We will exclude individuals who are on an NNRTI-based regimen unless they can be switched to another regimen.

Subjects will be counseled during the informed consent process regarding the benefits and risks of interrupting antiretroviral therapy. Specifically, subjects will be informed about the risk that an ATI has on transmission. This risk will be re-enforced throughout the study and discussed at each visit during the treatment interruption phase.

<u>ART re-initiation criteria.</u> The most optimal way to interrupt therapy in HIV remission studies is the topic of intense debate within the field. Some have argued that ART should be resumed once detectable viremia is concerned, as we are doing in our local studies[130] and as being done in ACTG 5345 (a treatment interruption protocol using time-to-rebound as an endpoint). Others have argued that for a study to be truly informative, high-level viremia may be needed; such an approach is being taken in some of the more recently approved studies[5] and a recently posted gene therapy study (NCT03617198). A study we are performing with GS-9620 has criteria that are in the middle of these extremes; in this study, we are effectively allowing people to have viremia for over 10,000 copies RNA/mL for up to four weeks before resuming ART (NCT03060447).

Our protocol is based on three recently completed studies of SIV/SHIV-infected macaques. In each study, an immunotherapy was given during ART and then treatment was discontinued. Most cases of a remission had a period of acute but transient viremia before virus control was observed. Viremia was often of high magnitude and lasted for weeks.

The first study providing rationale for our protocol was performed by Borducchi, Barouch and colleagues [10]. In this study of SIV-infected macaques, a prime-boost vaccine strategy followed by a TLR7 agonists resulted in either partial control or an apparent remission. High levels of viremia that persisted for weeks were required before the immune system could achieve virus control (see Figure).

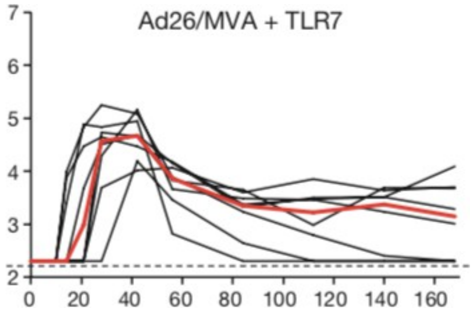

The second study was performed by Nishimura, Nussenzweig, Martin and colleagues[131]. In this study of SHIV-infected macaques, a short-course of bNAbs resulted in sustained virus control after a period of post-antibody acute viremia. At least four of the six animals who eventually controlled SHIV had a period of acute viremia. This resurgence in virus replication may be necessary to stimulate and expand the virus-specific T cell population.

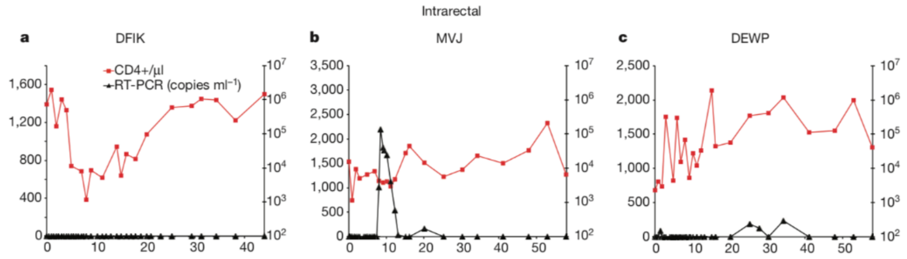

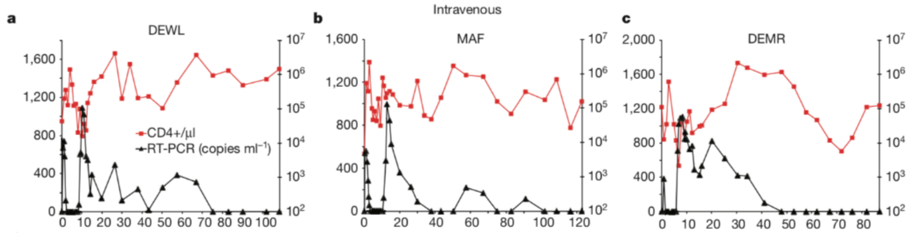

The final study was performed by Barouch and colleagues and has not yet been published (Barouch et al, CROI 2018). In this study, a bNAB was combined with at TLR7 agonist during ART. A few of the treated macaques appeared to have been cured. The remaining had a period of acute viremia before control was obtained.

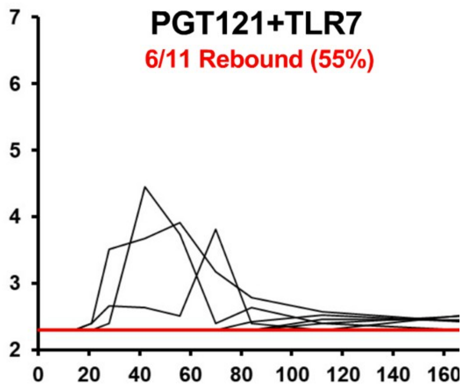

In formulating our study design, we also considered what happens among those individuals who are on ART, interrupt therapy and eventually achieve control ("post-treatment controllers") as this is the clinical phenotype we are trying to generate therapeutically. Few studies have carefully characterized the acute viremic stage in post-treatment controllers. In a recent study performed at the NIH, people who started therapy during early infection, stopped therapy and eventually exhibited some degree of control generally but not universally had a brief period of relatively low-level viremia. Still, some had very high-level viremia for a few weeks (see Figures)[5]

Similar data were recently reported by an extended collaboration that included several post-treatment controllers from our group (the CHAMP Cohort)[132].

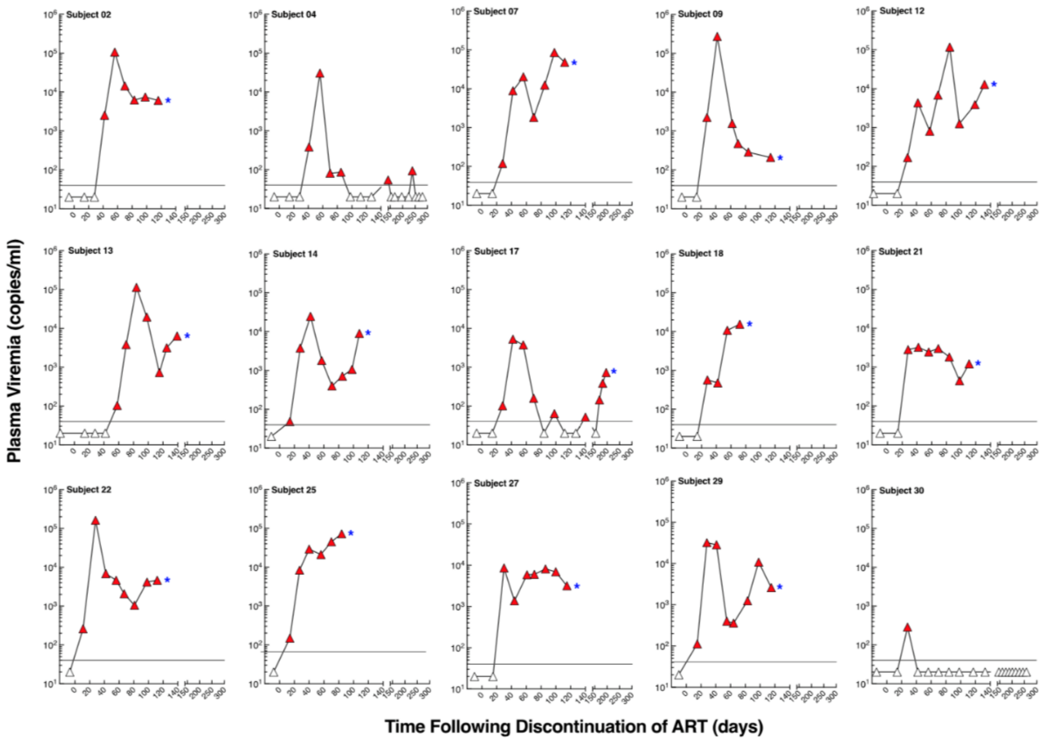
Subject 02
Subject 04
Subject 07
Subject 09
Subject 12
Subject 13
Subject 14
Subject 17
Subject 18
Subject 21
Subject 22
Subject 25
Subject 27
Subject 29
Subject 30
Plasma Viremia (copies/ml)
Time Following Discontinuation of ART (days)

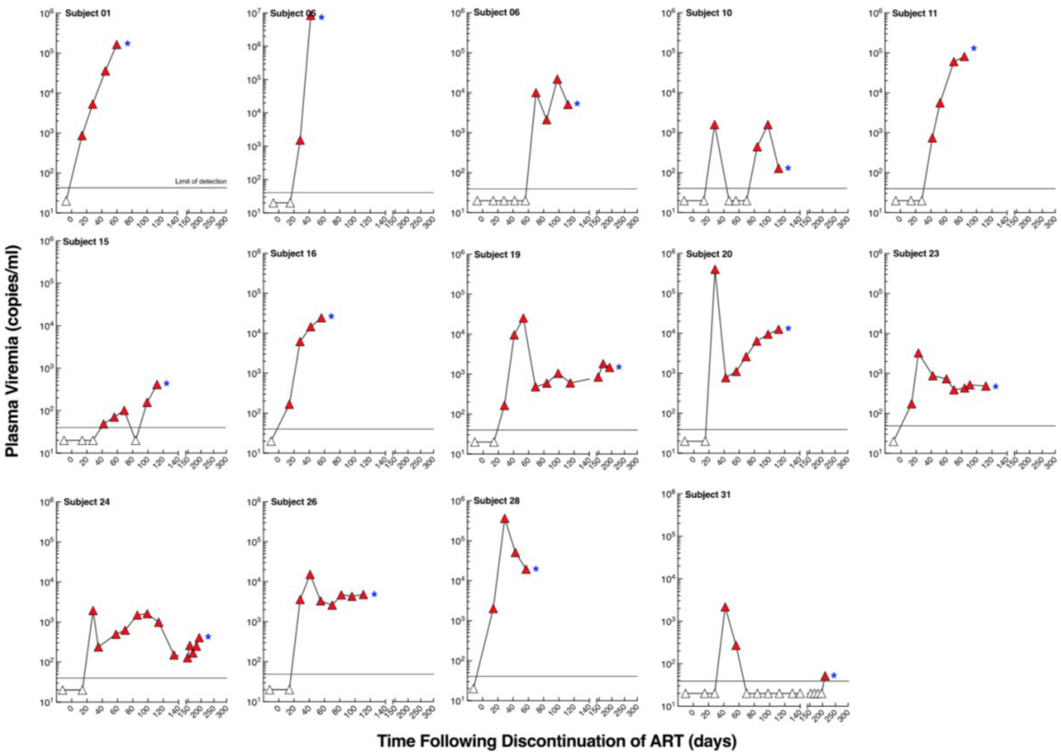

Based on this rationale, we propose that in addition to the standard CD4+ T cell count criteria (confirmed decline to below 350 cells/mm3) and clinical criteria (acute retroviral syndrome) that treatment be initiated for any one of the following outcomes: (1) viremia > 50,000 copies RNA/mL for four weeks, (2) viremia > 10,000 copies RNA/mL for 6 weeks, (3) viremia > 2000 copies RNA/ml for 12 weeks or (4) viremia > 400 copies/RNA for 24 weeks. Operationally, this will allow for an acute viremic state that will need to resolve within four to 12 weeks, with high-level viremia only being allowed for a brief period. Post-treatment control (defined as viremia < 400 copies RNA/mL) should be achieved prior to Week 24 post-virus rebound, or ART will be initiated.

In addition to these viral load criteria, we will resume therapy for a confirmed decline in CD4+ T cell count to less than 350 cells/mm^3 or clinical symptoms, including the acute retroviral syndrome, which is discussed below.

We are aware that interrupting ART has potential for significant risks. We will use a conservative study design to minimize risk. All individuals will be on a regimen that includes antiretroviral drugs with rapid clearance. This will minimize the chance that drugs will persist long enough to select for drug resistance in the rebounding virus. We will only include individuals with high CD4+ T cell counts to minimize the risk of developing clinically relevant immunodeficiency once the virus rebounds. We will monitor

individuals during the study, and resume therapy for a persistently high viral load, low CD4+ T cell count or the development of symptomatic disease.

Subjects will be counseled during the informed consent process regarding the benefits and risks of interrupting antiretroviral therapy. Specifically, subjects will be informed about the risk that an ATI has on transmission. This risk will be re-enforced throughout the study and discussed at each visit during the treatment interruption phase.

2 STUDY RATIONALE

2.1 Study Rationale and Hypothesis

Combination approaches will almost certainly be required to generate durable control of HIV in the absence of antiretroviral therapy (a "remission").

Based on studies in "elite" controllers and other chronic virus infections, durable control of HIV will likely require potent and sustained HIV-specific CD8+ T cells that target conserved epitopes. An IL-12 adjuvanted HIV DNA plasmid vaccine designed to generate subdominant responses to conserved regions within gag (the "p24 Conserved Element", or p24CE vaccine) has demonstrated impressive immunogenicity in macaques, particularly when boosted with a viral vector (MVA).

In a recent study of SIV-infected macaques on effective antiretroviral therapy, a therapeutic vaccine strategy alone was insufficient in preventing SIV rebound after antiretroviral therapy (ART) was discontinued. In contrast, this same vaccine strategy followed by a TLR7 agonist resulted in durable control of SIV in a subset of animals. In this study, Ad26 was administered as the prime. This was followed 12 weeks later by an MVA boost. During peak immunity (two weeks after first MVA administration) the TLR7 agonist (GS-9860) was initiated and given every two weeks for 10 doses (Weeks 50 to 70). A second MVA dose was given at Week 60.

In another recent study of SIV-infected macaques on effective antiretroviral therapy, a bNAb alone or a TLR7 agonist alone had a modest effect in achieving a remission. In contrast, the combination of both lead to an apparent cure in some animals and a remission in the remaining animals. A "shock and kill" effect on the reservoir likely occurred during ART, resulting in clearance or reduction of the reservoir. A sustained CD8+ T cell and/or NK cell response likely contributed to control of the virus after ART was interrupted.

Finally, recent data from the SHIV non-human primate model suggests that bNAbs can alter the immune response in treated animals undergoing a treatment interruption. Strong CD8+ T cell responses are generated in this setting, presumably as the bNAbs and HIV antigens generate immune complexes that stimulate antigen-presenting cells.

Based on these studies, we predict that sequential exposure to (a) a highly immunogenic heterologous prime/boost vaccine (DNA/MVA) vaccine, (b) a combination of bNAbs and a TLR9 agonist and (c) bNAb administration during a treatment interruption will reduce the reservoir size during ART and generate robust CD8+ T cell and NK cell responses, resulting in sustained and potent control after therapy is interrupted.

We believe that this approach is more likely to be efficacious than a single intervention. For years, scientists have been calling to abandon single intervention cure trials. Just as we learned that single agent ART, and later 2-agent ART (with older agents), was insufficient to control plasma HIV RNA levels, we are at the phase of HIV cure interventions at which a combination approach will likely be necessary to control the virus. We believe that the proposed combination has the potential to prolong the time participants will be off ART compared with each intervention used alone.

2.2 Risk / Benefit Assessment

There may be no direct benefit for the study participant. The primary benefit is the gain in knowledge regarding a potentially effective intervention that may in future studies allow for a durable control of HIV in absence of therapy (a remission). It is possible that the regimen may allow the participant to remain on ART for weeks to months, if not longer.

Each of the planned interventions has risk. The planned treatment interruption poses risk to both the participant and his or her sexual partners. All risks will be discussed during the informed consent process and all possible interventions aimed at mitigating this risk will be used.

2.3 Program Overview

The central premise of our program is that durable control of HIV in the absence of antiretroviral therapy ("remission") will require both the generation of *de novo* potent and sustained immune responses that target evolutionarily conserved epitopes and a low reservoir size. In an open-label, single arm study we test the impact of a combination intervention aimed at achieving a sustained immune response, a low reservoir size and ultimately a remission. We will study those who initiated ART during early infection (n=15) or chronic infection (n=5). All subjects will receive the same treatment strategy and all will undergo a "set-point" treatment interruption.

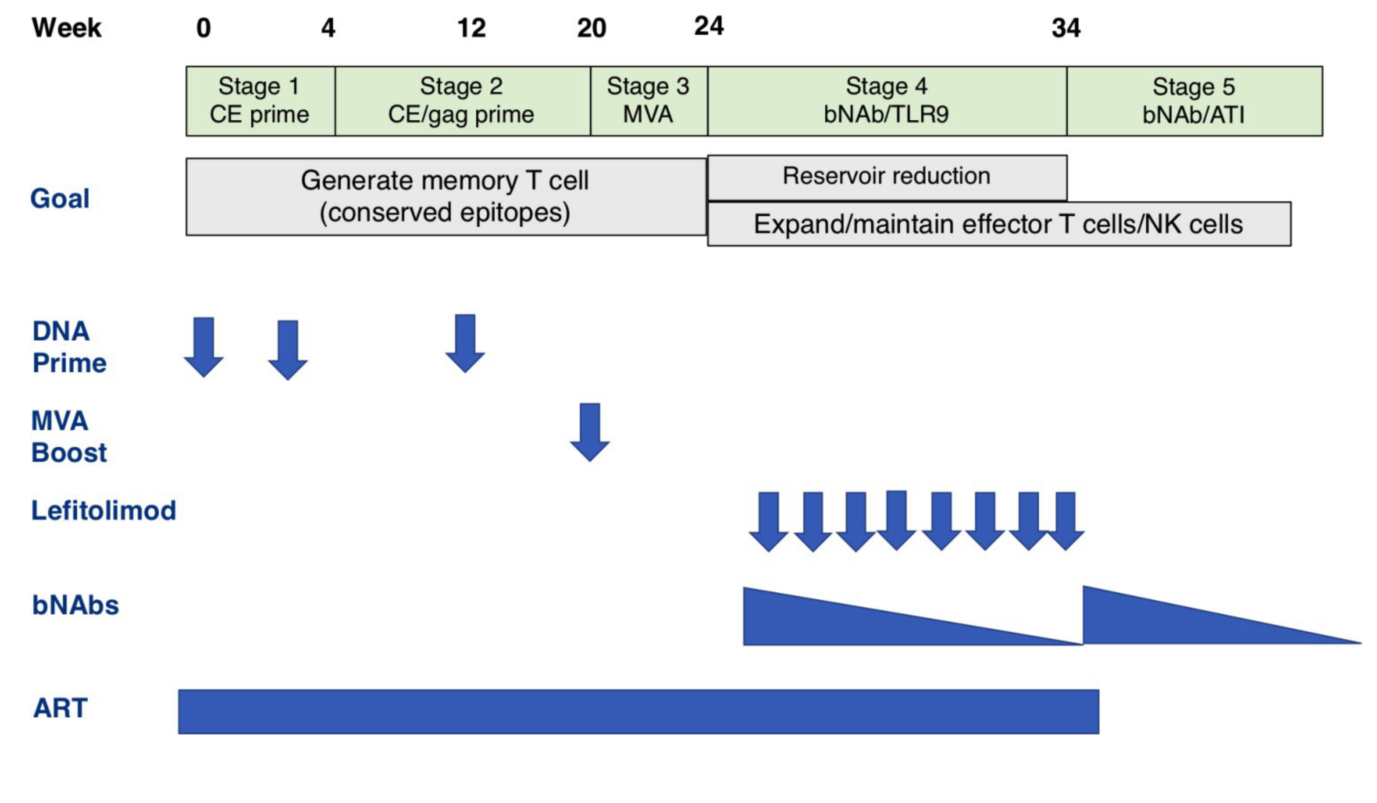

In this proof-of-concept pilot study, eligible HIV infected adults who started therapy during acute/recent infection (Early Cohort) or chronic infection (Chronic Cohort) will receive a combination immunotherapy aimed at achieving a long-term remission. Stage 1: Subjects will first be vaccinated with HIV DNA vaccines expressing 7 unique conserved elements from $p24^{Gag}$ (p24CE) at Weeks 0 and 4. IL-12 DNA plasmids will be co-administered as a vaccine adjuvant. The DNA plasmids will be administered by electroporation. Stage 2: Subject will be vaccinated with p24CE DNA and full-length $p55^{gag}$ DNA at week 12. IL-12 DNA plasmids will be co-administered as a vaccine adjuvant. The DNA plasmids will be administered by electroporation. The goal is to expand the responses to subdominant conserved regions. Stage 3: Subjects will receive an MVA vaccine expressing Gag, Pol, and Env at Week 20. It is expected that the CE-specific responses will be preferentially boosted. Stage 4: A combination of two bNAbs (10-1074 and VRC07-532LS) will be administered at week 24. Subjects will then receive a series of lefitolimod subcutaneous injections weekly between Weeks 25 and 33. This stage has three goals: to enhance DNA/MVA vaccine responses, to generate potent NK cell responses and to reverse latency (which will allow vaccine-induced T cells and bNAbs to reduce the reservoir size). Stage 5: ART will be interrupted at Week 34. Two days prior to the interruption an additional dose of the bNAbs will be administered. Lefitolimod dosing will be discontinued one week prior to the ATI. The goal of this stage is to enhance CTL memory and effector responses (during the post-viral rebound state, when antibodies will capture and present antigen to APCs). Should this combination regimen work, viral load would be expected to rebound transiently after the bNAb levels wane. This would be followed by a new lower viral load and perhaps a long-term remission. Note: As of Protocol Version 2.2, no further lefitolimod will be administered.

3 STUDY OBJECTIVES

3.1 Primary Objectives

- To evaluate the safety and tolerability of combination regimen in which subjects will receive EP-administered IL-12 adjuvanted DNA vaccine (prime, Stages 1 and 2), heterologous MVA (boost, Stage 3), combination bNAbs/TLR9 adjuvant (Stage 4) and bNAbs during an ATI (ATI, Stage 5).
- To evaluate the impact of this 5-stage regimen on viral load set-point during a treatment interruption

3.2 Secondary Objective

- To evaluate the immunogenicity of this this 5-stage regimen
- To evaluate the anti-reservoir activity of this this 5-stage regimen

4 STUDY DESIGN

4.1 Study Overview

We will perform an open-label, single-arm proof-of-concept study of a four-stage curative intervention in 20 HIV-infected adults for whom ART was initiated during early (n=15) or chronic (n=5) infection. All subjects will receive the same regimen and interrupt ART.

- Stage I (DNA prime): Subjects will first be vaccinated with HIV DNA vaccines expressing 7 unique conserved elements (CE) from $p24^{Gag}$ at Weeks 0 and 4. An IL-12 DNA plasmid will be co-administered with the HIV DNA plasmids. DNA will be administered with electroporation.
- Stage 2 (DNA boost): These responses will then be boosted by a combination of DNA vaccines containing p24CE and full-length gag (p55) at Week 12. An IL-12 DNA plasmid will be co-administered with two HIV DNA plasmids. DNA will be administered by electroporation.
- Stage 3 (MVA boost): Subjects will receive an MVA boost at Week 20.
- Stage 4: A combination of two bNAbs (10-1074 and VRC07-523LS) will be administered at Week 24, with therapeutic levels expected to persist through Weeks 32 to 34. Starting at Week 25 and continuing through Week 33 (10 weeks), subjects will receive a weekly dose of lefitolimod. Note: As of Protocol Version 2.2, no further lefitolimod will be administered.
- Stage 5: An additional dose of the bNAbs will be administered at two days prior to Week 34. ART will be interrupted at Week 34.

5 CRITERIA FOR EVALUATION

5.1 Primary Endpoints

- Safety and tolerability
- Plasma HIV-1 RNA levels during an ATI

5.2 Secondary Endpoint

- Number, magnitude, polyfunctionality, and cytotoxic phenotype of CE-targeted T cell responses elicited by the vaccination and maintained in the peripheral blood throughout the study as measured by intracellular cytokine staining (ICS) after stimulation with CE-derived peptides. Responses to Gag, Pol, Env, and Nef overlapping peptide pools will be evaluated in parallel
- Frequency of circulating CD4+ T cells harboring replication-competent HIV as measured using multiplex digital droplet PCR assay to quantify the total number of intact proviruses CD4+ T cell counts

5.3 Other Endpoints

- Phenotypic characteristics (e.g., expression of PD-1, TIGIT, CD160, 2B4, Tbet, TCF-1) of *de novo* vaccine-induced (CE-specific) HIV-specific CD8+ T cell populations (as detected using MHC class I-specific tetramers) compared to longitudinally-studied pre-existing HIV-specific CD8+ T cell populations
- Phenotypic characteristics (e.g., expression of CD69, Ki-67) of innate immune cells using mass cytometry (CyTOF)
- Transcriptomic analysis of FACS-sorted tetramer-detectable HIV-specific CD8+ T cell populations using RNA-Seq
- Transcriptomic analysis of longitudinally-collected whole blood samples
- Functional features of HIV-specific antibodies (ADCC, phagocytosis, complement-mediated destruction, neutrophil activation, and dendritic cell uptake)
- Plasma HIV RNA (single copy assay)
- Cell-associated RNA
- Cell-associated DNA

6 SUBJECT SELECTION

6.1 Study Population

Subjects with HIV infection who meet the inclusion and exclusion criteria will be eligible for participation in this study.

Our primary target population will be adults on long-term ART who started therapy during early HIV infection. Acute and early HIV infection has historically been defined using the Fiebig system, which requires laboratory data that is no longer routinely acquired. The system is outlined the table and figure below.

		Duration, mean (range), days	
Stage	Defining finding and/or marker	Individual phase	Cumulative duration
Eclipse	…	10 (7–21)	10 (7–21)
I	vRNA positive	7 (5–10)	17 (13–28)
II	p24 antigen positive	5 (4–8)	22 (18–34)
III	ELISA positive	3 (2–5)	25 (22–37)
IV	Western blot positive or negative	6 (4–8)	31 (27–43)
V	Western blot positive, p31 antigen negative	70 (40–122)	101 (71–154)
VI	Western blot positive, p31 antigen positive	Open-ended	…

NOTE. ELISA, enzyme-linked immunoassay; vRNA, viral RNA.

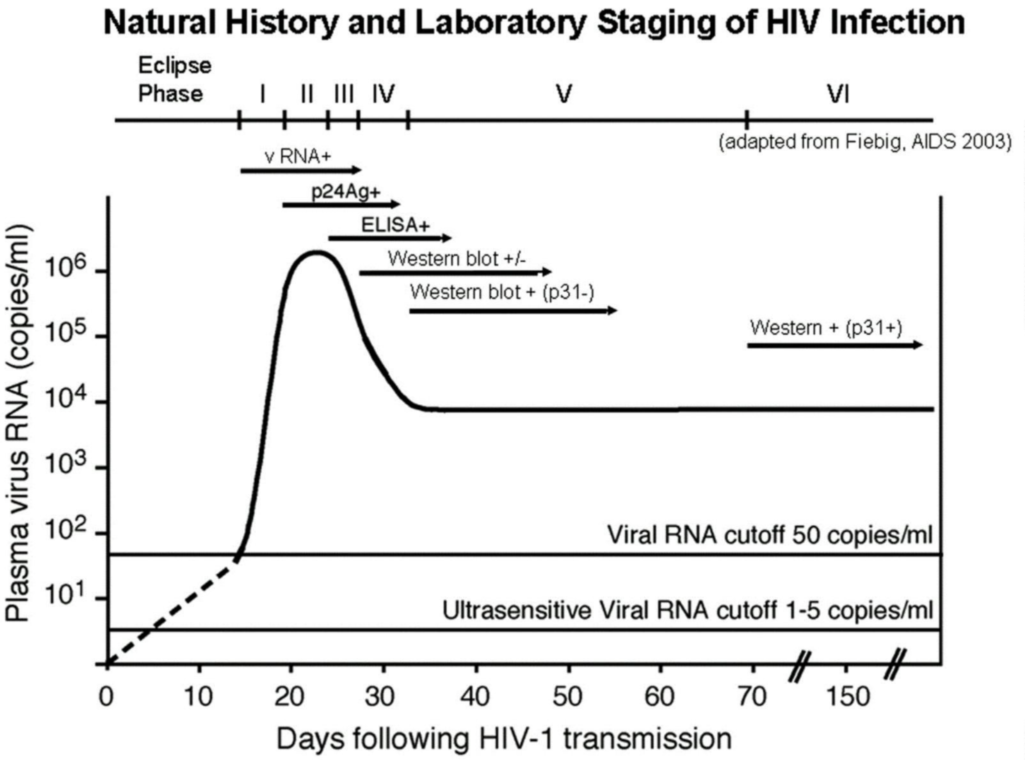

The CDC has recently developed a system that allows for the calculation of plausible date of detectable HIV infection (EDDI). This algorithm is based on evaluating seroconversion panels from 50 individuals who were frequent plasma donors (about 2-3 times per week) for approximately a 3-month seroconversion record. Using serial testing on every currently marketed HIV test, the CDC group, has defined that the average delay from first viremia to the appearance of first positivity using any one of these tests. The individuals' sexual exposure history and prior HV testing are incorporated into the calculation. Other factors that contribute to the algorithm include testing history (an HIV negative test in previous three months would be diagnostic of early infection), level of viremia, and the antibody test signal-to-cutoff ratios. By inputting data into a web-based system, the EDDI is calculated. The system also provides a likely interval during which the EDDI likely occurred, and reports both an early probable interval limit for date of detected infection (EP-DDI) and late probable interval limit for date of detected infection (LP-DDI). More information on the system is available at https://tools.incidence-estimation.org/idt/.

We have been using this EDDI in our cohort for years. We will use this approach to identify our population.

Our group has also calculated the estimated infection date (EID) using clinical history and an analysis of the antibody profiles, as characterized using the Western Blot. These calculations are listed in order of preference as outlined below:

1) First Positive Test at Options = Negative EIA and/or Negative Western Blot with detectable viral load. Positive Western Blot confirmed by subsequent repeat testing. EID = 24 days before date of first positive test.
2) First Positive Test = Indeterminate Western Blot. Positive Western Blot confirmed by subsequent repeat testing. EID = 24 days before date of first positive test.
3) First Positive Test = Positive Western Blot. Copy available showing last Negative HIV test within 180 days. EID = 24 days before the midpoint between last negative test and first positive test.

We will recruit HIV-infected adults who started ART in early infection from our local cohort (Options) and from other centers in the city. As the method used to diagnose early infection has varied over the past two decades and across centers, we will use either the Fiebig, EDDI or EDI system outlined above to define acute/early infection. We will seek to enroll individuals who appeared to start ART during early infection (first six months) using any of these classification systems.

6.2 Inclusion Criteria

1. Willing and able to provide written informed consent
2. Male, female, or transgender
3. Age ≤65 years at the time of enrollment.
4. HIV-1 infection, documented by any of the following:
    a. licensed rapid HIV test or HIV enzyme or chemiluminescence immunoassay (E/CIA) test kit at any time prior to study entry and confirmed by a licensed Western blot or a second antibody test by a method other than the initial rapid HIV and/or E/CIA
    b. 4th Generation HIV-1 Ag/Ab test
    c. HIV-1 antigen or plasma HIV-1 RNA viral load as confirmed by the Principal Investigator
    d. Research assay result compatible with a diagnosis of HIV infection (e.g. detectable intracellular HIV DNA or viral outgrowth assay) as confirmed by the Principal Investigator
5. On continuous antiretroviral therapy for at least 12 months without any interruptions of greater than 14 consecutive days within the last 1 year, and on a stable regimen that does not include an non-nucleoside reverse transcriptase inhibitor (NNRTI) for at least 4 weeks at the time of first study product administration, without plans to modify ART during the study period
6. ART initiated within 6 months of estimated infection date (early ART, n=15) or after six months of their estimated infection date (late ART, n=5). A participant must meet the definition for Early infection using an estimated infection date by at least one method: EID, EDDI, or Fiebig staging (I-V) at diagnosis.
7. Screening plasma HIV RNA levels below the level of quantification on all available determinations in past 12 months (isolated single values above the level of quantification but < 200 copies/mL will be allowed if they were preceded and followed by unquantifiable viral load determinations)

8. Screening CD4+ T-cell count ≥ 500 cells/mm^3
9. Creatinine Clearance (CrCl) > 60 mL/min via Cockroft-Gault method at screening
10. The following laboratory criteria must be met at screening:
    - Absolute neutrophil count (ANC) ≥ 1000 neutrophils/mm^3
    - Hemoglobin ≥ 10.0 g/dL
    - Platelet count ≥ 100,000/uL
    - Aspartate aminotransferase (AST) ≤ 2x upper limit of normal (ULN)
    - Alanine aminotransferase (ALT) ≤ 2x ULN
    - Total, direct and indirect bilirubin ≤ ULN

6.3 Exclusion Criteria

1. Subjects receiving a non-nucleoside reverse transcriptase inhibitor, unable to switch to a different regimen.
2. Pregnant, breastfeeding, or unwilling to practice birth control (see below) during participation in the study
3. Acceptable birth control is defined as the following:
    a. For female participants of childbearing potential[1], two of the following forms of contraception are required, one of which must be a barrier method: (1) condoms (male

---

[1] The assessment of reproductive potential will be at the discretion of the PI. As guidance, a patient will be considered to be of reproductive potential unless they meet the following criteria:

a) Appropriate medical documentation of prior hysterectomy and/or complete bilateral oophorectomy (i.e., surgical removal of the ovaries, resulting in "surgical menopause" and occurring at the age at which the procedure was performed),

<u>OR</u>

b) Permanent cessation of previously occurring menses as a result of ovarian failure with documentation of hormonal deficiency by a certified healthcare provider (i.e., "spontaneous menopause"). Hormonal deficiency should be properly documented in the case of suspected spontaneous menopause as follows:

1. If age >54 years and with the absence of normal menses for at least 24 consecutive months: serum follicle stimulating hormone (FSH) level elevated to within the post-menopausal range based on the laboratory reference range where the hormonal assay is performed;
2. If age ≤ 54 years and with the absence of normal menses for at least 24 consecutive months: Negative serum or urine (â-HCG) performed within 48 hours prior to study entry with concurrently elevated serum FSH level in the post-menopausal range, depressed estradiol (E2) level in the post-menopausal range, and absent serum progesterone level, based on the laboratory reference ranges where the hormonal assays are performed.

OR

c) Male-to-female transgender individual lacking reproductive organs requisite for reproduction.

or female) with or without a spermicidal agent, (2) diaphragm or cervical cap with spermicide, (3) intrauterine device (IUD) with published data showing that expected failure rate is < 1% per year, (4) tubal ligation, (5) hormone-based contraceptive such as oral birth control pills

   b. Male participants participating in sexual activity that could lead to pregnancy must agree to at least one reliable method of contraception of the above listed

4. High-level resistance to both 10-1074 and VRC-07 as defined using the PhenoSense Neutralizing Antibody Assay (Monogram Biosciences). The clinical thresholds for defining activity with this or other assays are unknown. We will exclude individuals whose virus susceptibility (IC50) is in the top 10% for at least one of the antibodies or in the top 25% for both antibodies. Individuals who have previously measured phenotypic susceptibility to 10-1074 and VRC-07 do not need to have this test repeated as long as they have been on suppressive ART as outlined below (point b), although it may be repeated at the discretion of the PI.
   a. Samples assayed may demonstrate amplification failure due to a low reservoir state or other issues causing assay failure. These are individuals for whom it will be impossible to identify resistance and most experts agree that these individuals are likely to have susceptible virus. For individuals for whom the Monogram PhenoSense assay is unable to make a determination, we will consider this a lack of evidence for resistance. Such individuals will be eligible to participate in the study.
   b. In situations in which a participant has prior data from the Monogram PhenoSense assay available, we will use the prior result regardless of timing as long as the individual has not interrupted therapy between the timepoint of the test and enrollment in the trial. We will define this as continual ART without evidence of plasma HIV RNA elevated above the assay limit of detection, allowing for isolated levels above the limit of detection if they are less than 200 copies/mL (“blips”).
5. Any history of an HIV-associated malignancy, including Kaposi’s sarcoma and any type of lymphoma, or virus-associated cancers.
6. Active or recent non-HIV-associated malignancy requiring systemic chemotherapy or surgery in the preceding 36 months or for whom such therapies are expected in the subsequent 12 months; minor surgical removal of localized skin cancers (squamous cell carcinoma, basal cell carcinoma) are not exclusionary
7. CD4+ T cell nadir <350 cells/mm^3 during the chronic phase of infection (beginning 6 months following the estimated infection date and confirmed on repeat testing). Individuals with CD4+ T cell values <350 cells/mm^3 within 6 months of the estimated infection date which subsequently normalize will be eligible for inclusion.
8. Evidence of HIV “elite” control prior to ART as defined by having HIV RNA levels below the limit of detection without exposure to ART, using commercially available assays at that time
9. History of current active hepatitis B (HBV) infection defined as positive HBV surface antigen test.

10. Active hepatitis C (HCV) infection. Subjects must be HCV antibody negative or have evidence of cleared HCV infection. If the subject HCV antibody positive, an unquantifiable HCV RNA result (<LLOQ, either target detected or target not detected) within 42 days prior to study entry is required. Those who are currently receiving HCV antiviral therapy or those who have received HCV treatment in the last 6 months prior to study entry will be excluded.
11. Chronic liver disease including known compensated cirrhosis or uncompensated cirrhosis, as defined by the presence of ascites, encephalopathy, esophageal or gastric varices, or persistent unexplained jaundice.
12. Active and poorly controlled atherosclerotic cardiovascular disease (ASCVD), as defined by 2013 ACC/AHA guidelines, including a previous diagnosis of any of the following: (a) acute myocardial infarction, (b) acute coronary syndromes, (c) stable or unstable angina, (d) coronary or other arterial revascularization, (e) stroke, (f) transient ischemic attack (TIA), or (g) peripheral arterial disease presumed to be of atherosclerotic origin.
13. Presence of significant abnormalities on electrocardiogram
14. History of potential immune-mediated medical conditions (see Appendix A). Individuals with isolated Raynaud's phenomenon or localized disease requiring topical therapy alone will not be excluded.
15. Serious illness requiring systemic treatment and/or hospitalization in the 3 months prior to study enrollment
16. Concurrent treatment with immunomodulatory drugs, and/or exposure to any immunomodulatory drug in the 4 weeks prior to study enrollment (e.g. corticosteroid therapy equal to or exceeding a dose of 15 mg/day of prednisone for more than 10 days, IL-2, interferon-alpha, methotrexate, cancer chemotherapy). use of inhaled or nasal steroid is not exclusionary.
17. Exposure to any vaccination within 7 days of study enrollment or exposure to any experimental vaccination within 90 days of study enrollment. Study participants will be encouraged to get the influenza vaccine and any other routine vaccinations (including those for hepatitis A, hepatitis B and varicella zoster) prior to screening for the study. Routine influenza or other clinically-required vaccinations during the study period will be allowed.
18. Exposure to any experimental therapies within 90 days of study entry. Study subjects will not be allowed to receive other experimental therapies during the course of the study.
19. Serious medical or psychiatric illness that, in the opinion of the investigator, would interfere with the ability to adhere to study requirements or to give informed consent.
20. Active drug or alcohol use or dependence that, in the opinion of the site investigator, would interfere with adherence to study requirements or to give informed consent.
21. Serious medical or psychiatric illness that, in the opinion of the investigator, would interfere with participation in the trial may interfere with interpretation of study results or place the subject at risk for harm or injury.

22. Acute or chronic bleeding or clotting disorder that would contraindicate IM injections, or use of blood thinners (e.g. anticoagulants or antiplatelet drugs) within 2 weeks of Day 0
23. Less than two acceptable sites available for IM injection considering the deltoid and anterolateral quadriceps muscles
24. Individuals in whom the ability to observe possible local reactions at the eligible injection sites is, in the opinion of the investigator, unacceptably obscured due to a physical condition or permanent body art, or who have keloids or hypertrophic scars located within 2 cm of intended treatment site.
25. Implanted electronic medical devices (e.g., cochlear implant, pacemaker, implantable cardioverter defibrillator); ii) sinus bradycardia (defined as < 50 beats per minute on exam); or iii) history of cardiac arrhythmia (e.g., supraventricular tachycardia, atrial fibrillation, or frequent ectopy). Please note, sinus arrhythmia is not excluded.
26. Metal implants or implantable medical device within the intended treatment site (i.e. electroporation area)
27. ECG with clinically significant findings, or features that would interfere with the assessment of myo/pericarditis, including any of the following:(1) conduction disturbance (complete left or complete right bundle branch block or nonspecific intraventricular conduction disturbance with QRS ≥ 120 ms, PR interval ≥ 220ms, any 2nd or 3rd degree AV block, or QTc prolongation (> 450ms)); (2) repolarization (ST segment or T wave) abnormality that will interfere with the assessment of myo/pericarditis; (3) significant atrial or ventricular arrhythmia; (4) frequent atrial or ventricular ectopy (eg, frequent premature atrial contractions, 2 premature ventricular contractions in a row); (5) ST elevation consistent with ischemia; (6) evidence of past or evolving myocardial infarction. Serious medical or psychiatric illness that, in the opinion of the site investigator, would interfere with the ability to provide informed consent, adhere to study requirements, affect interpretation of the study results or place subject at risk for harm, e.g., a history of myo/pericarditis."
28. Known allergy/sensitivity or any hypersensitivity to components of study drug or their formulation, e.g., egg allergy (inability to consume eggs or baked goods containing eggs), allergy to any amide-type local anesthetic (such as bupivacaine (Marcaine®), lidocaine (Xylocaine®), mepivacaine (Polocaine® Carbocaine®), etidocaine (Duranest®), or prilocaine (Citanest®), EMLA® cream.
29. Individuals in whom a skin-fold measurement of the cutaneous and subcutaneous tissue for eligible injection sites exceeds 40 mm.
30. History of severe allergic reactions (e.g., angioedema, anaphylaxis) or severe reactions to prior vaccination (e.g., severe injection site reaction, anaphylaxis).
31. Chronic or active neurologic disorder which, in the opinion of the investigator, would place the participant at risk of harm or injury. Note that individuals with stable headaches, stable peripheral neuropathy, and stable post-herpetic neuralgia may be included.
32. Confirmed syncopal episode determined or suspected to be of cardiac origin within 12 months of screening.

33. Significant or progressive ophthalmologic disease, including significant signs or symptoms of active disease.

7 CONCURRENT MEDICATIONS

All subjects should be maintained on the same medications throughout the entire study period, as medically feasible.

Subjects must provide their own antiretroviral drugs. No restrictions will be placed on the antiretroviral drug combinations. If medically feasible, study participants will be encouraged to remain on their entry antiretroviral regimen through week 34 of the study.

Participation in the study will not interfere in any manner with the subject's standard of care.

Given the potential immune-modifying activities of the vaccine and IL-12 adjuvants, other immunomodulatory drugs (e.g., cytokines, systemic corticosteroids, most biologics) will be discouraged if medically feasible.

Routine or standard of care vaccinations (such as influenza, pneumococcal, and meningococcal vaccinations) are allowed and documented in the case report forms.

8 STUDY TREATMENTS

8.1 Product management

The Investigational Drug Service (IDS) of the UCSF Medical Center Department of Pharmaceutical Services will be responsible for storage of all investigational products for this study. The IDS facilities include USP797-compliant IV preparation facilities, biohazardous preparation facilities and secure drug storage areas for drugs requiring monitored controlled room temperature, refrigeration (2-8 degrees C.), moderate freezing (-15 to –25 degrees C.) and deep cold storage (below –70 degrees C.). All facilities and departmental procedures are subject to inspection and oversight by the Board of Pharmacy, California State Department of Health, CMS and the Joint Commission.

The IDS has a long track record of supporting NIH-sponsored studies and has worked extensively with Susan Buchbinder's HVTN unit. They have hence been through the rigorous DAIDS regulatory process and have in the recent past successfully supported local studies of VRC01 and other biologic products.

IAVI will manage the distribution of all investigation products. Working with the UCSF-based research pharmacy directed by Dr. Scott Fields, IAVI will collect and distribute all investigational products to the clinical site, and relabel any vials or cartons as needed. The cold-chain system will be reviewed and audited to ensure that all products remain viable. A 'critical document checklist' will be employed before shipping. Once all protocols and documents are established and checked, IAVI will and oversee shipment of each product. The group will proper cold chain for IP storage at the clinical site and generate a Cold Chain Assessment report.

8.2 HIV DNA Expression Plasmids

[redacted]

[redacted]

[redacted]

[redacted]

[redacted]

[redacted]

[redacted]

[redacted]

[redacted]

[redacted]

[redacted]

[redacted]

[redacted]

[redacted]

[redacted]

[redacted]

8.3 GENEVAX® IL-12 pDNA Vaccine Adjuvant

[redacted]

[redacted]

[redacted]

[redacted]

8.4 TDS-IM v1.0 Electroporation Device

[redacted]

[redacted]

[redacted]

[redacted]

[redacted]

[redacted]

[redacted]

[redacted]

[redacted]

[redacted]

8.5 MVA62B

[redacted]

[redacted]

[redacted]

[redacted]

8.6 10-1074

[redacted]

8.7 VRC07-523LS

[redacted]

- [redacted]
- [redacted]

[redacted]

8.8 Lefitolimod

[redacted]

- [redacted]

[redacted]

[redacted]

9 STUDY PROCEDURES

Prior to conducting any study-related activities, written informed consent and the Health Insurance Portability and Accountability Act (HIPAA) authorization must be signed and dated by the subject.

Blood collection will occur at visits as outlined in the Schedule of Events and will be timed to stay within Red Cross Guidelines (less than approximately 480 mL every 8 weeks).

Given the expected age of our study population (most will likely be at least 50 years old) we expect many participants to have a number of chronic medical conditions, which might change over time. The interval between the screening visit and the first study vaccination will be limited to 60 days.

All subjects will be observed for adverse reactions for at least 30 minutes after administration of any of the study products and at least 4 hours following administration of bNAbs. .

Study specific case report forms will be administered for data collection and clinical assessments as described in Sections 9.1-9.3.

Laboratory personnel and those involved in raw data analysis of the endpoint assays (Sections 9.12-9.15) will be blinded with regards to participant identity and study time point.

9.1 Concomitant Medications

All concomitant medications taken within 30 days prior to screening and entry and a complete history of ART, HIV-1 related vaccines, and immune-based therapies will be documented at the Screening and Entry visits.

At each subsequent visit, all additions or discontinuations of prescription medications should be recorded. Actual or estimated start and stop dates should be recorded.

9.2 Demographics and Medical History

Demographic information (date of birth, sex, race and ethnicity) will be recorded at Screening.

At Screening, the first Baseline visit (B1) and Entry (Day 0), the medical history must include all diagnoses within the past 30 days and, regardless of when the diagnosis was made, a complete history of chronic conditions, malignancies, and AIDS-defining conditions. Self-reported or documented nadir CD4+ T cell count should be recorded.

Any allergies to any medications or their formulations should also be recorded.

9.3 Clinical Assessment

Signs and Symptoms

At pre-entry, all grades of signs and symptoms that occurred 30 days prior to the visit must be recorded.

At entry and all post-entry visits, all grades of signs and symptoms that occurred since the previous visit must be recorded. Duration (start and stop dates and times), severity/grade, outcome, treatment and relation to study drug will be recorded on the study-specific case report form (CRF). Criteria for participant management, dose interruptions, modifications, and discontinuation of treatment will be mandated only for toxicities attributable to the study medication(s).

Diagnoses

All clinical events and new diagnoses or changes in diagnoses should be recorded.

9.4 Physical Examination

Complete Physical Exam

A complete physical examination should be conducted at screening. It is to include at minimum an examination of the skin, head, mouth, and neck, auscultation of the chest, cardiac and abdominal exam, examination of the lower extremities for edema, and Karnofsky performance score. The complete physical exam will also include resting vital signs (temperature, pulse, respiratory rate, and blood pressure), height and weight.

Targeted Physical Exam

A targeted physical examination should be conducted at each subsequent visit following screening. It is to include resting vital signs (temperature, pulse, respiratory rate, blood pressure) and weight, and is to be driven by any new signs or symptoms that the participant has experienced since the last visit.

Eye Exams

Due to rare ocular toxicity reported with bNAb administration, participants will undergo ophthalmologic exams at screening and in the period between Week 12-16 of the ATI (study Weeks 46-50). If a participant discontinues treatment prior to receiving bNAbs a follow-up ophthalmologic exam will not be required. Should premature discontinue occur after bNAb administration but prior to Weeks 12-16 of the ATI, an ophthalmologic exam will be conducted within 16 weeks the last bNAb dose. An ophthalmologist affiliated with the study will be available if patients experience ocular symptoms during the course of the study. Ophthalmologic services will be provided by the Department of Ophthalmology at Zuckerberg San Francisco General Hospital. The content of these exams will be at the discretion of the ophthalmologist, but may include dilation of the pupils.

9.5 Clinical Laboratory Measurements

Hematology

Complete blood count (hemoglobin, hematocrit, red blood cell count, white blood cell count, white blood cell differential, and platelet count) will be obtained at screening, the first baseline visit (B1), entry, and all post-entry visits through week 24. CBCs will be obtained every two weeks during W24-34, and then every four weeks through the end of the study.

Chemistry

Serum sodium, potassium, chloride, bicarbonate, random glucose, blood urea nitrogen (BUN), creatinine, aspartate aminotransferase (AST/SGOT), alanine aminotransferase (ALT/SGPT), alkaline phosphatase, total bilirubin, and albumin will be obtained at screening, entry, and most post-entry visits, as detailed in Section 10. Note that total and direct bilirubin will be measured at screen and at all chemistry timepoints from Week 24 onwards.

Pregnancy Testing

A serum or urine β-human chorionic gonadotropin HCG test (urine test must have a sensitivity of 25 mIU/mL) will be obtained from female subjects with reproductive potential at screening and frequently

throughout the study (see Schedule of Events). Specifically, a serum or urine pregnancy test will be performed and reviewed on day of and prior to each study injection during stages 1-3 (DNA vaccines and MVA), prior to administering bNAbs (beginning of Stage 4), and every two weeks during lefitolimod administration (Stage 4), prior to second administration of bNAbs and the ATI (beginning of Stage 5) and every four weeks during the ATI (Stage 5). As of Protocol Version 2.2, no further lefitolimod will be administered.

9.6 HIV Clinical Laboratory Measures

Blood will be obtained and sent to a CLIA-certified or equivalent laboratory for real-time determination of absolute CD4+ and CD8+ T cell counts, percentages, and CD4/CD8 ratio and plasma HIV-1 RNA quantification (viral load) at most visits, as detained in section 10. All efforts will be made to have collection of blood for T cell counts occur between 8:00 AM and 11:00 AM, in order to account for diurnal variation in CD4+ T cell count.

9.7 Cryopreservation of Plasma

Plasma will be collected and stored at the indicated visits.

9.8 Cryopreservation of PBMCs

PBMCs will be collected and stored at the indicated visits.

9.9 Collection of PAXgene specimen

Whole blood will be collected in PAXgene tubes at the indicated visits.

9.10 Pharmacokinetic Measurements

Broadly neutralizing antibody (10-1074, VRC07-523LS) levels will be measured at indicated visits during Stages 4 and 5. Plasma samples will be collected every 2 weeks for 10 weeks during Stage 4 (bNAbs/lefotimod) and every two weeks for 24 weeks during the Stage 5 (bNAbs/ATI).

VRC07 levels will be measured at the Vaccine Immunology Testing Laboratory (VITL) located at the VRC using well-validated assays. Concentrations of VRC07-523LS for the PK analyses will each be measured by qualified ELISAs using a Beckman Biomek based automation platform. Briefly, the VRC07-23LS anti-idiotypic MAb is coated onto Immulon-4HXB microtiter plates overnight at 4° C. Plates are washed and blocked (10% FBS in PBS) for 2 hours at room temperature. Duplicate serial 3-fold dilutions covering the specific range (100 – 218,700) of the test sample are incubated for 2 hours at 37°C followed by horseradish peroxidase - labeled goat anti-human antibody (1 hour, 37° C), and TMB substrate (15 minutes, room temperature). Color development is stopped by addition of sulfuric acid and plates are read within 30 minutes at 450 nm via the Molecular Devices Paradigm plate reader. Linear regression of a standard curve of VRC07-523LS covering the linear range is utilized to quantitate the sample concentrations of each antibody based upon the average of sample dilutions within the range of the assay.

Similarly, 10-1074 levels will be measured at using a validated ELISA; it is expected that these assays will be performed by Georgia Tomaras and her colleagues using a BMGF-funded core laboratory.

9.11 PhenoSense Neutralizing Antibody Assay

Neutralizing antibody susceptibility will be determined using the PhenoSense neutralizing antibody assay platform pioneered by Monogram Biosciences, Laboratory Corporation of America Holdings. This assay

generates HIV pseudovirions that express envelope proteins that are representative of the quasispecies of either the cell-associated HIV DNA or circulating plasma HIV RNA. This will enable the interrogation of neutralizing antibody susceptibility of PBMC-derived virus from aviremic individuals. Procedures are derived from a well-established assay that has been utilized extensively to evaluate autologous and vaccine elicited neutralizing antibody sensitivity of virions pseudotyped with a population of envelope proteins that are representative of the HIV RNA quasispecies in circulating plasma virus from viremic individuals[147].

[redacted]

[redacted]

[redacted]

The assay will be performed in a CLIA/CAP compliant laboratory.

Samples assayed may demonstrate amplification failure due to a low reservoir state or other issues causing assay failure. These are individuals for whom it will be impossible to identify resistance and most experts agree that these individuals are likely to have susceptible virus.

We will implement this exclusion as follows:

For individuals for whom the Monogram PhenoSense assay is unable to make a determination, we will consider this a lack of evidence for resistance. Such individuals will be eligible to participate in the study.

In situations in which a participant has prior data from the Monogram PhenoSense assay available, we will use the prior result regardless of timing as long as the individual has not interrupted therapy for more than 2 weeks between the timepoint of the test and enrollment in the trial. We will define this as continual ART without evidence of plasma HIV RNA elevated above the assay limit of detection, allowing for isolated levels above the limit of detection if they are less than 200 copies/mL ("blips").

9.12 Cellular Immunologic Measurements

T cell immunogenicity. High-resolution multi-parameter flow cytometric intracellular cytokine staining (ICS) assays will be used to longitudinally monitor the induction, differentiation, and durability of HIV-specific T cell responses in the peripheral blood. PBMCs will be stimulated *in vitro* with peptides from each of the seven CE regions, or peptide pools of overlapping 15mers derived from Gag, Pol, Env, or Nef (using potential T cell epitope peptides, PTE; NIH AIDS Reagent Program). ICS will be performed on two baseline PBMC samples, and these will be compared to newly-generated T cell responses detectable in PBMCs after the electroporations (Week 20), at the peak of the vaccine response (Week 22 of the study, 2 weeks after the MVA boost), pre-ATI (Week 34 of the study, after a dose of bNAbs and 9 weekly lefitolimod injections), at Week 38 and 42 (approximately when bNAb levels are anticipated to become undetectable), and at Week 54.

The focus of our immunogenicity studies will be to measure: (1) the number of new CE-targeted CD4+ and CD8+ T cell responses elicited by therapeutic vaccination, as well as (2) the magnitude, (3) polyfunctionality (number of effector cytokines including IFN-γ, TNF-α, and IL-2 are by responding cells), and (4) cytotoxic phenotype (expression of Perforin, Granzyme B and degranulation as measured by CD107a upregulation) of the CE-targeted responses. Because the vaccine regimen may also stimulate non-CE Gag responses (with the vaccine dose that includes p55 Gag), as well as Pol and/or Env responses (with the MVA62B vaccine), we will assess these broader responses in parallel (with Nef as a negative control).

Tetramer analysis of CD8+ T cell exhaustion phenotype. One of the major goals of HIV therapeutic vaccination is to determine whether it is possible to induce functional HIV-specific CD8+ T cell responses that are not exhausted. At the baseline and peak time points (Week 22 for post-vaccine evaluation and Week 34 for post-lefitolimod/bNAb evaluation), we will characterize in more depth the phenotype and transcriptional state of existing and vaccine-elicited HIV-specific CD8+ T cell responses using MHC Class I tetramers. We will identify and follow longitudinally pre-existing and *de novo* vaccine-induced HIV-specific CD8+ T cell populations before and after vaccination and characterize CD8+ T cell exhaustion in these populations by evaluating the expression of multiple well-established phenotypic markers of exhaustion, as well as their transcriptomic signature (see below).

Innate Immune Cell Phenotypes. Previous studies suggest that treatment with lefitolimod will stimulate innate immune responses (e.g., dendritic cell and monocyte activation) and that bNAbs may stimulate NK cell responses. To evaluate the impact of our proposed regimen on these parameters, we will perform high-dimensional single-cell analysis of longitudinally sampled PBMC specimens using mass cytometry (CyTOF) panels designed to measure the simultaneous expression level of up to 40 different cell surface and intracellular markers on individual cells.

These measurements will be performed at baseline, Week 1, Week 22, after the last vaccination but prior to administration of bNAb/lefitolimod (Week 24), Week 38, Week 42, and Week 54

As of protocol Version 2.2, no further lefitolimod will be administered. Time points for immunologic measurements may be varied for participants not receiving lefitolimod as outlined in prior protocol versions.

9.13 Transcriptomic Measurements

The Sekaly Laboratory will perform transcriptomic analysis of (1) whole blood (collected in PAXgene tubes) and (2) FACS-sorted tetramer-detectable populations (pre-existing and vaccine-induced) using RNA-Seq methods optimized and validated for whole blood and small cell populations[148] and analyzed using validated bioinformatic pipelines for gene expression analysis. Transcriptomic profiling of longitudinal samples will enable characterization of vaccine and cytokine adjuvants on host gene expression. In addition, consideration of the gene expression data within the context of the aforementioned immunologic measurements will allow us to identify specific host transcriptomic correlates of vaccine responsiveness.

These measurements will be performed at baseline, when peak post-vaccine memory responses are assessed (for tetramer+ cells: Week 22), prior to the administration of lefitolimod/bNAb (for whole blood RNA-Seq: Week 24), prior to ATI (Week 34), and at weeks 38, 42, and 54.

9.14 Antibody Measurements

The Alter Laboratory will perform a suite of antibody functional assays covering a range of effector mechanisms (ADCC, phagocytosis, complement-mediated destruction, neutrophil activation, and dendritic cell uptake) carried out by a diverse set of innate effector cells (monocytes, NK, dendritic cells, and neutrophils) coupled to Systems Biology computational analyses[149]. This approach will be utilized to assess antibody functionality induced following therapeutic vaccination. This "Systems Serology" platform co-interrogates the biophysical features (antibody isotype/subclass and glycosylation), with all functional features to define the specific biophysical characteristics of both bulk and HIV-specific antibodies, creating a function:biophysical features map that may point to the specific antibody subpopulations that may 1) serve as biomarkers of enhanced reservoir control or 2) highlight mechanisms by which antibodies can contribute to control of the viral reservoir.

These measurements will be performed at baseline, prior to bNAb administration (at Week 24), and at the end of the trial (End Study visit).

9.15 Virologic Measures

Since the mQVOA only detects intact proviruses that are induced by a single round of stimulation *in vitro* and replicate in MOLT cells[150], the Siliciano Laboratory will use a novel and scalable multiplex digital droplet PCR assay to measure the *total* number of intact proviruses (the Intact Proviral DNA Assay, IPDA). The IPDA was developed as a rapid and scalable PCR test to precisely quantify latent HIV-1 in infected individuals using a small biological sample[151-153]. This approach requires a cell input of 2-5 million CD4+ T cells. This novel assay will allow us to determine the number of infected cells with intact viral genomes in multiple samples from a large number of study participants as well as generally assess the composition of the persistent proviral landscape in each sample.

These virologic measurements will be performed at baseline, Week 24, and Week 54. These measurements will also be performed 24 weeks after re-initiation of ART.

Ultrasensitive qPCR diagnostics will be used to quantify HIV RNA to single-copy levels.

Cell-associated (CA) total RNA[154] and integrated DNA[155] will be quantified using qPCR assays where nucleic acid input is normalized to cell number.

9.16 Sample processing and specimen management

Within the UCSF SCOPE infrastructure, initial sample processing is performed on peripheral blood and leukapheresis units. All samples at UCSF and UCLA are tracked through Laboratory Information Systems, such as LDMS, or in-house systems with sample storage inventories, multiple report formats, and Institutionally-managed data storage and back-up systems.

The SCOPE Program will use the AIDS Specimen Bank (ASB) for preserving and distributing study specimens. ASB provides an established infrastructure with expertise in specimen processing and storage. It has received >188,000 deposits and has distributed >192,000 specimen aliquots worldwide. Specimens are stored in ultra-low temperature freezers with back-up power systems, in which temperature is monitored by a programmable scanning alarm system wired into the university's telephone system.

10 EVALUATIONS BY VISIT

10.1 Overview

Participants will be consented by the Principal Investigator or the research team before any procedures take place.

Once a participant is identified as potentially eligible by review of screening criteria and is to be scheduled for a screening visit, a unique identifier (4-digit SCOPE ID) will be assigned. Once a SCOPE ID is assigned, it cannot be reassigned to another subject.

Subjects who satisfy all inclusion/exclusion criteria will be enrolled into the study, which is summarized in the Schedule of Events.

A brief medical history will be obtained at each visit. We will collect, analyze and report all available data related to any potentially immune-mediated medical conditions (PIMMCs, Appendix A) through week 86.

Blood collection will occur at all visits as per the Schedule of Events and will be timed to stay within Red Cross Guidelines (less than 480 mL every 8 weeks).

Given the expected age of our study population (most will likely be at least 50 years old) we expect many participants to have a number of chronic medical conditions, which might change over time. The interval between the screening visit and the first study vaccination will be limited to 60 days.

All subjects will be observed for adverse reactions for at least 30 minutes after administration of any of the final study product and at least 4 hours after administration of bNAbs.

Pregnancy and HIV transmission precautions will be discussed extensively throughout the study. For women of childbearing potential, pregnancy testing will be performed at a minimum as per the Schedule of Events and at other visits as needed per the discretion of PI/Co-I, and the results discussed at each visit. The need to prevent pregnancy will be discussed as needed throughout the study. The risk for transmitting HIV to others will be discussed in detail and at each visit during the treatment interruption phase (Stage 5). All study participants will be informed that virus rebound could happen at any time during the interruption, and that high-level viremia may occur even in the absence of symptoms. The risk that any virus rebound poses to sexual partners will be discussed. Methods to prevent HIV transmission to others will also be discussed. See Appendix B for risk mitigation strategies.

Study specific case report forms will be administered for data collection and clinical assessments as described in Sections 9.1-9.3.

10.2 Procedures: Staggered enrollment

Given limited clinical experience with some of the components of the combination regimen, subjects will be enrolled in a staggered fashion. Specifically, a pilot group of 3 individuals will start vaccinations four weeks before opening up the study to the others.

10.3 Screening and baseline evaluations

Screen: Prior to conducting any study-related activities, written informed consent and the Health Insurance Portability and Accountability Act (HIPAA) authorization must be signed and dated by the subject.

All participants are assigned a unique four-digit number. For those already enrolled in SCOPE, we will continue to use their existing number. New participants will be assigned a unique SCOPE number during screening. Once a SCOPE ID is assigned, it cannot be reassigned to another subject.

A medical history and physical examination will be performed. Participants will undergo an ophthalmologic examination between screen and prior to study entry.

Hematology, metabolic/chemistry and coagulation (PT/PTT) studies will be performed. Hepatitis B and C studies will be performed. Individuals with prior documentation Hepatitis B immunity will not require Hepatitis B testing. Individuals with prior documentation of negative Hepatitis C antibody and who lack new risk factors will not require retesting of Hepatitis C antibody although it may be repeated at the discretion of the PI.

A pregnancy test will be performed in women with child-bearing abilities.

Plasma HIV RNA levels and CD4+/CD8+ T cell counts will be measured.

A screening test to assess for phenotypic susceptibility to 10-1074 and VRC-07 (PhenoSense Neutralizing Antibody Assay) will be performed by Monogram Biosciences. Individuals who have previously measured phenotypic susceptibility to 10-1074 and VRC-07 do not need to have this test repeated if they have remained on suppressive ART (as outlined above), although it may be repeated at the discretion of the PI.

An electrocardiogram will be performed.

Screening evaluations should be completed within 60 days prior to study entry.

Baseline visit 1 (B1, week -4 to day -3): Plasma HIV RNA levels and CD4+/CD8+ T cell counts will be measured. Routine safety laboratory tests (hematology and chemistry laboratories) will be performed. Plasma and PBMCs will be collected and cryopreserved. Serum may be collected (see B2D0 below).

10.4 p24CE and IL-12 DNA vaccinations (Stage 1)

Entry /baseline visit 2 (B2 D0): The second baseline visit will occur.

Routine safety studies (hematology and chemistry laboratories) will be performed. Plasma HIV RNA levels and CD4+/CD8+ T cell counts will be measured.

Plasma and PBMCs will be collected and cryopreserved. Serum may be collected (must be collected on B2D0 or within 4 weeks prior).

A PAXgene specimen will be collected.

A urine pregnancy test will be performed and evaluated prior to vaccination (in females of childbearing potential). If the urine pregnancy test is positive, the positive result will be confirmed with a serum pregnancy test. If the serum pregnancy test is positive, all study interventions will be discontinued.

Subjects will initiate treatment. Study product (p24CE vaccine and IL12 DNA plasmids) will be administered by electroporation. Subjects will be observed for 30 minutes after vaccine administration to assess for immediate injection site reactions and adverse events.

Day 1: Subjects will be contacted by telephone to check on symptoms and overall response to the injections.

Week 1: This visit will take place 7 +/- 2 days from the date of the B2D0 visit. Routine safety studies will be performed. Plasma HIV RNA levels and CD4+/CD8+ T cell counts will be measured. Plasma and PBMCs will be collected and cryopreserved. A PAXgene specimen will be collected.

Week 4: Routine safety studies will be performed. Plasma HIV RNA levels and CD4+/CD8+ T cell counts will be measured.

A urine pregnancy test will be performed and evaluated prior to vaccination (in females of childbearing potential). If the urine pregnancy test is positive, the positive result will be confirmed with a serum pregnancy test. If the serum pregnancy test is positive, all study interventions will be discontinued.

The second p24CE and IL-12 DNA vaccination by electroporation will occur. Subjects will be observed for 30 minutes after vaccine administration to assess for immediate injection site reactions and adverse events.

Week 4 + 1 Day: Subjects will be contacted by telephone to check on symptoms and overall response to the injections.

10.5 p24CE, p55^{gag} and IL-12 DNA vaccinations (Stage 2)

Week 12: Routine safety studies will be performed. Plasma HIV RNA levels and CD4+/CD8+ T cell counts will be measured.

Plasma and PBMCs will be collected and cryopreserved.

A urine pregnancy test will be performed and evaluated prior to vaccination (in females of childbearing potential). If the urine pregnancy test is positive, the positive result will be confirmed with a serum pregnancy test. If the serum pregnancy test is positive, all study interventions will be discontinued.

The third DNA vaccination (p24CE, p55^{gag} and IL-12 DNA plasmids) by electroporation will occur. Subjects will be observed for 30 minutes after vaccine administration to assess for immediate injection site reactions and adverse events.

10.6 MVA vaccination (Stage 3)

Week 20: Routine safety studies will be performed. Plasma HIV RNA levels and CD4+/CD8+ T cell counts will be measured.

Plasma and PBMCs will be collected and cryopreserved.

A urine pregnancy test will be performed and evaluated prior to MVA administration (in females of childbearing potential). If the urine pregnancy test is positive, the positive result will be confirmed with a serum pregnancy test. If the serum pregnancy test is positive, all study interventions will be discontinued.

The MVA boost will be given by intramuscular injection. Subjects will be observed for 30 minutes after vaccine administration to assess for immediate injection site reactions and adverse events.

Week 20+1: Subjects will be contacted by telephone to check on symptoms and overall response to the injection.

Week 22: Routine safety studies will be performed. Plasma HIV RNA levels and CD4+/CD8+ T cell counts will be measured.

Plasma and PBMCs will be collected and cryopreserved.

A PAXgene specimen will be collected.

10.7 Combination bNAbs and lefitolimod administrations (Stage 4)

Week 24: Routine safety studies will be performed. Plasma HIV RNA levels and CD4+/CD8+ T cell counts will be measured. Samples will be stored to assess the impact of DNA/MVA prime-boost regimen on peak effector HIV-specific T cells, the primary immunologic outcome measurement.

Plasma and PBMCs will be collected and cryopreserved. Serum will be collected. A PAXgene specimen will be collected.

A urine pregnancy test will be performed and evaluated prior to bNAb infusion (in females of childbearing potential). If the urine pregnancy test is positive, the positive result will be confirmed with a serum pregnancy test. If the serum pregnancy test is positive, all study interventions will be discontinued.

The first dose of combination bNAbs will be administered. Subjects will be observed for 4 hours after bNAb administration to assess for immediate adverse events.

Week 24+1: Subjects will be contacted by telephone to check on symptoms and overall response to the combination bNAbs infusion.

Week 25-33: Lefitolimod (TLR9 agonist) will be administered subcutaneously weekly beginning one week (+/-3 days) after the bNAbs are administered and ending one week before the ATI. Following the initial dose at Week 25, the weekly lefitolimod doses may be administered on any day of the week provided the doses are at least 5 days apart. A total of 9 doses will be administered during this period. **Note: As of Protocol version 2.2 (Version Date 9/21/21) no further lefitolimod will be administered. See Appendix E. Participants who already received lefitolimod will continue with monitoring as below.**

Routine safety monitoring studies will be performed at weeks 24, 26, 28, 30, and 32.

Plasma HIV RNA levels and CD4+/CD8+ T cell counts will also be measured at weeks 24, 28, 30, and 32.

Plasma and PBMCs will be collected and cryopreserved as outlined in Section 10.12.

Among women of childbearing potential, urine pregnancy test will be performed and evaluated prior to the first dose of lefitolimod (Week 24) then every two weeks (Weeks 26, 28, 30 and 32). If the urine pregnancy test is positive, the positive result will be confirmed with a serum pregnancy test. If the serum pregnancy test is positive, all study interventions will be discontinued.

Participants will be asked about the development of new ophthalmologic symptoms at every visit.

10.8 Combination bNAbs and treatment interruption phase (Stage 5)

Week 34:

Staggered Entry into Week 34 (Protocol Version 2.2)

Participants due to enter Stage 5 will have liver function tests checked within 2 weeks prior to their Week 34 visit. If ALT, AST, and total bilirubin meet criteria for grade 1 or higher (AST or ALT 1.25 x upper limit of normal (ULN), total bilirubin 1.1 x ULN), LFTs will be repeated approximately every 1-2 weeks. The team will only proceed with the second bNAb dose and ATI if/once AST, ALT, and total bilirubin are below the grade 1 threshold.

A study physician will perform a targeted hepatobiliary review of symptoms and exam in all participants on the day of and prior to administration of bNAbs. The study team will review all concomitant medications and counsel participants to discontinue any potentially hepatotoxic agents for the duration of the study.

Participants will be staggered for Stage 5 (bNAbs/ATI) entry. One participant will enter Stage 5 initially, with LFTs checked at weeks 1 and 2 following bNAbs/ATI. If these LFTs remain below the grade 1 threshold, two more participants will enter Stage 5 and have LFTs checked 1 and 2 weeks after bNAbs/ATI. If these LFTs remain below the grade 1 threshold, the remaining 2 participants will enter Stage 5.

Study Procedures

Provided that most recent HIV RNA level was below the level of quantification, subjects will take the last dose of antiretroviral drugs on the day before arriving to the clinic for the Week 34 visit. LFTs will also be checked on the day of the bNAb visit, prior to bNAb administration, to obtain a pre-bNAb/ATI baseline measure.

A second infusion of combination bNAbs will be administered at the time ART is discontinued. Subjects will be observed for 4 hours after bNAb administration to assess for immediate adverse events.

A urine or pregnancy test will be performed and evaluated in females of childbearing potential. If the urine pregnancy test is positive, the positive result will be confirmed with a serum pregnancy test. If the serum pregnancy test is positive, the subject will continue ART.

Hematology, plasma HIV RNA levels and CD4+/CD8+ T cell counts will be measured.

Plasma and PBMCs will be collected and cryopreserved. Serum will be collected.

A PAXgene specimen will be collected.

<u>Week 34+1:</u> Subjects will be contacted by telephone to check on symptoms and overall response to the combination bNAbs infusion.

<u>Weeks 34 to 86 (ATI period):</u> Subjects will be evaluated in the clinic or via a home/work RN visit weekly during the interruption period. Plasma HIV RNA levels will be measured at each visit; it is expected that the results will be available within about 4 business days.

Routine safety studies will be performed every four weeks. CD4+/CD8+ T cell counts will be measured every two weeks. In all participants, the LFT monitoring frequency will be increased to every week for four weeks, then every 2 weeks from Week 38 to Week 42, followed by monthly monitoring.
If any grade 1 or higher LFT elevation is detected, the LFTs will be repeated to confirm resolution (return to below grade 1 threshold) before additional participants enter Stage 5. If any grade 2 or higher LFT elevation is detected, further entry into Stage 5 will be halted and a review conducted by the SMC.

Plasma and PBMCs will be collected and cryopreserved as outlined in Section 10.12. Serum will be collected at the week 58 visit. Note that in cases in which clinical laboratory testing does not occur at the study site (for example, plasma HIV RNA testing arranged locally for someone living outside San Francisco), storage of specimens may be deferred.

A urine pregnancy test will be performed and evaluated every four weeks during the ATI (in females of childbearing potential). If the urine pregnancy test is positive, the positive result will be confirmed with a serum pregnancy test. If the serum pregnancy test is positive, ART will be resumed immediately.

A PAX Gene specimen will be collected at W38, W42, and W54.

Between week 46 and week 50, a routine eye exam will be repeated by an ophthalmologist.

Participants will be asked about the development of new ophthalmologic symptoms at every visit and referred to an ophthalmologist to evaluate new symptoms of any grade.

10.9 ART resumption phase (Stage 6)

Viral load criteria. Based on this rationale, we propose that treatment be initiated for any one of the following outcomes: (1) viremia > 50,000 copies RNA/mL for four weeks, (2) viremia > 10,000 copies RNA/mL for 6 weeks, (3) viremia > 2000 copies RNA/ml for 12 weeks or (4) viremia > 400 copies/RNA for 24 weeks. Operationally, this will allow for an acute viremic state that will need to resolve within 12 weeks, with high-level viremia only being allowed for a brief period. Post-treatment control (defined as viremia <400 copies RNA/mL) should be achieved prior to Week 24 post-virus rebound.

For any initial viral load greater than 400 copies/mL, we will bring individuals back immediately (within 3 working days or as soon as they are available) for a repeat test.

CD4+ T cell criteria. ART will also be reinitiated if the CD4+ T cell count declines to below 350 cells/μL on two consecutive visits. If an individual demonstrates a CD4+ T cell count <350 cells/uL, we will bring them back immediately (within 3 working days or as soon as they are available) for a repeat test.

Clinical criteria. ART will also be re-initiated for any symptoms suggestive of a severe acute retroviral syndrome, particularly signs of altered mental status or viral meningitis (severe headache, neck stiffness), but including unexplained rash, lymphadenopathy, and fevers. ART will also be re-initiated at the request of the study participant and/or her primary health care provider.

Post-ART monitoring. Once ART is reinitiated, subjects will be evaluated at Weeks 4 and 8 on ART. If viral load is not below the level of quantification by Week 8, participants will be evaluated every four weeks until the viral load is confirmed to less that the level of quantification. A follow up visit will occur 24 weeks after resumption of ART.

Routine safety studies, CD4+/CD8+ T cell counts and viral load will be performed at Weeks 4, 8, 24 and at any visits required between Week 8 and Week 24 to evaluate decrease of viral load to below level of quantification. Plasma and PBMCs will be collected and cryopreserved at the time of ART resumption and at Weeks 4, 8 and 24 on ART.

10.10 End of study

Week 86: Subjects who receive study products through week 34 will be followed through 86. Should subjects initiate ART late in the follow-up phase, the last visit might be extended. Routine safety studies will be performed every four weeks during ART. CD4+/CD8+ T cell counts will be measured every four weeks. Plasma and PBMCs will be collected and cryopreserved at the end of the study.

Given the potential for delay immune-mediated adverse events, we will collect, analyze and report all data related to any medically attended adverse events (MAAEs), including potentially immune-mediated medical conditions (PIMMCs, Appendix A). All participants will be followed for 52 weeks after their last exposure to an experimental immunotherapy (Week 86, which is 53 weeks after the last dose lefitolimod and 52 weeks after the last dose of the bNAbs). Following the updates to Protocol Version 2.2 and the withholding of lefitolimod for some participants, Week 86 will no longer be 53 weeks following the last dose of lefitolimod for all enrolled participants.

10.11 Premature discontinuation

Routine safety studies will be performed. Plasma HIV RNA levels and CD4+/CD8+ T cell counts will be measured. Plasma and PBMCs will be collected and cryopreserved and any other specimen collection deemed pertinent to safety or data integrity as assessed by PI/Co-I.

Participants who discontinue prematurely will be have an end of study visit scheduled 52 weeks after the last dose of study product administration. This will include routine safety studies, CD4+/CD8+ T cell counts, plasma and PBMCs cryopreservation, and any other specimen collection deemed pertinent to safety or data integrity as assessed by PI/Co-I.

10.12 Schedule of events

Schedule of Events: DNA/MVA Vaccine (Screen to Week 22, Stages 1-3)

Note: Visit windows are +/- 1 week for vaccines during Stage 1-3

DNA/MVA Vaccine (Screen Week to 22, Stages 1-3)	S	B1	B2 D0	Wk 1	W4	W12	W20	W22
p24CE/IL-12			1		1			
p24CE&p55/IL-12						1		
MVA							1	
Lefitolimod								
bNAbs								
ATI								
Home Nurse Visit (Optional)								
Concomitant Medications	1	1	1		1	1	1	1
PE (symptom)	1	1	1		1	1	1	1
Clinical Assessment	1	1	1		1	1	1	1
Hematology/Chemistry	1	1	1	1	1	1	1	1
T cell counts	1	1	1	1	1	1	1	1
Plasma HIV RNA	1	1	1	1	1	1	1	1
Pregnancy testing	1		1		1	1	1	
bNAb levels								
Cryopreservation plasma		1	1	1		1	1	1
Cryopreservation PBMCs		1	1	1		1	1	1
LVBD		1	1				1	1
Leukapheresis (optional)		1*						
LN FNA (optional)		1*						1
GALT (optional)		1*						1
Eye Exam	1#							
ECG	1							
PAXGene			1	1				1
PhenoSense Neutralizing Antibody Assay	1							
Serum storage		1^						

*Optional baseline studies may be performed at any visit after screen through B2D0. #Eye exam may be done at any visit from S prior to B2D0. ^Serum can be stored at any visit within 4 weeks of or on B2D0

Schedule of Events: bNAb/TLR9 (Week 24-33, Stage 4)

Note: Visit windows are +/- 1 week for bNAbs. Week 25 lefitolimod window is 7 days and +/- 3 days after the bNAbs. Following the initial dose of lefitolimod, weekly doses may be administered on any day of the week provided the doses are at least 5 days apart.

	W24	W25	W26	W27	W28	W29	W30	W31	W32	W33	
**p24CE/IL-12**											
**p24CE&p55/IL-12**											
**MVA**											
**Lefitolimod**		1	1	1	1	1	1	1	1	1	Note: No more lefitolimod will be administered as of V2.2.
**bNAbs**	1										
**ATI**											
**Home Nurse Visit (Optional)**											
**Concomitant Medications**	1	1	1	1	1	1	1	1	1	1	
**PE (symptom)**	1	1	1	1	1	1	1	1	1	1	
**Clinical Assessment**	1	1	1	1	1	1	1	1	1	1	
**Hematology/Chemistry**	1		1		1		1		1		
**T cell counts**	1				1		1		1		
**Plasma HIV RNA**	1				1		1		1		
**Pregnancy testing**	1		1		1		1		1		
**bNAb levels**			1		1		1		1		
**Cryopreservation plasma**	1		1		1		1		1		
**Cryopreservation PBMCs**	1				1				1		
**LVBD**	1										
**Leukapheresis (optional)**											
**LN FNA (optional)**											
**GALT (optional)**											
**Eye Exam**											
**ECG**											
**PAXGene**	1										
**PhenoSense Neutralizing Antibody Assay**											
**Serum storage**	1										

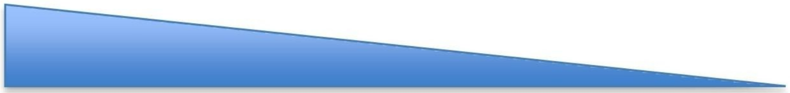

bNAb Levels

Schedule of Events: bNAb/ATI (Week 34-47, Stage 5)

Note: Visit window is +/- 1 week for bNAbs during stage 5

	W34	W35	W36	W37	W38	W39	W40	W41	W42	W43	W44	W45	W46	W47
p24CE/IL-12														
p24CE&p55/IL-12														
MVA														
Lefitolimod														
bNAbs	1													
ATI	1	1	1	1	1	1	1	1	1	1	1	1	1	1
Home Nurse Visit (Optional)		1	1	1	1	1	1	1	1	1	1	1	1	1
Concomitant Medications	1	1	1	1	1	1	1	1	1	1	1	1	1	1
PE (symptom)	1	1	1	1	1	1	1	1	1	1	1	1	1	1
Clinical Assessment	1	1	1	1	1	1	1	1	1	1	1	1	1	1
Hematology/Chemistry	1	1	1	1	1		1		1				1	
T cell counts	1		1		1		1		1		1		1	
Plasma HIV RNA	1	1	1	1	1	1	1	1	1	1	1	1	1	1
Pregnancy testing	1				1				1				1	
bNAb levels	1		1		1		1		1		1		1	
Cryopreservation plasma	1		1		1		1		1		1		1	
Cryopreservation PBMCs	1				1				1				1	
LVBD	1								1					
Leukapheresis (optional)	1													
LN FNA (optional)	1													
GALT (optional)	1													
Eye Exam													1^	1^
ECG														
PAXGene	1								1					
PhenoSense Neutralizing Antibody Assay														
Serum storage	1													

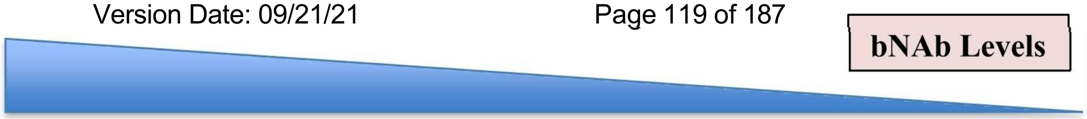

Schedule of Events: Extended ATI (Week 48-86, Stage 5-Extended)

	W48	49	50	51	52	53	54	55	56	57	58	60	62	64	66	68	70	72	74	76	78	80	82	84	86 or End Study$^{\Delta}$	ART re-initiation or Early Treatment Discontinuation
**p24CE/IL-12**																										
**p24CE&p55/IL-12**																										
**MVA**																										
**Lefitolimod**																										
**bNAbs**																										
**ATI**	1	1	1	1	1	1	1	1	1	1	1	1	1	1	1	1	1	1	1	1	1	1	1	1	1	1
**Home Nurse Visit (Optional)**	1	1		1	1	1	1	1	1	1	1	1	1	1	1	1	1	1	1	1	1	1	1	1	1	1
**Concomitant Medications**	1	1	1	1	1	1	1	1	1	1	1	1	1	1	1	1	1	1	1	1	1	1	1	1	1	1
**PE (symptom)**	1	1	1	1	1	1	1	1	1	1	1	1	1	1	1	1	1	1	1	1	1	1	1	1	1	1
**Clinical Assessment**	1	1	1	1	1	1	1	1	1	1	1	1	1	1	1	1	1	1	1	1	1	1	1	1	1	1
**Hematology/Chemistry**			1				1				1		1		1		1		1		1		1		1	OPT
**T cell counts**	1		1		1		1		1		1	1	1	1	1	1	1	1	1	1	1	1	1	1	1	1
**Plasma HIV RNA**	1	1	1	1	1	1	1	1	1	1	1	1	1	1	1	1	1	1	1	1	1	1	1	1	1	1
**Pregnancy testing**			1				1				1		1		1		1		1		1		1		1	1
**bNAb levels**	1		1		1		1		1		1															OPT
**Cryopreservation plasma**	1		1		1		1		1		1		1				1				1				1	1
**Cryopreservation PBMCs**			1				1				1		1				1				1				1	1
**LVBD**							1																		1	OPT
**Leukapheresis (optional)**																										OPT
**LN FNA (optional)**																										OPT
**GALT (optional)**																										OPT
**Eye Exam**	1^	1^	1^																							1*
**ECG**																										
**PAXGene**							1																			OPT
**PhenoSense Neutralizing Antibody Assay**																										OPT
**Serum storage**											1														1	OPT

- Schedule of Events: Post ART Follow up (Weeks 4, 8 and 24 after ART initiation, Stage 6)
- Note: Visit windows are +/- 1 week for Stage 6

Post ART-initiation: weeks 4, 8, and 24 on ART	W4-ART	W8-ART	W24-ART
p24CE/IL-12			
p24CE&p55/IL-12			
MVA			
Lefitolimod			
bNAbs			
ATI			
Home Nurse Visit (Optional)			
Concomitant Medications	1	1	1
PE (symptom)	1	1	1
Clinical Assessment	1	1	1
Hematology/Chemistry	1	1	1
T cell counts	1	1	1
Plasma HIV RNA	1	1	1
Pregnancy testing			
bNAb levels			
Cryopreservation plasma	1	1	1
Cryopreservation PBMCs	1	1	1
LVBD			1
Leukapheresis (optional)			
LN FNA (optional)			
GALT (optional)			
Eye Exam			
ECG			
PAXGene			
PhenoSense Neutralizing Antibody Assay			
Serum storage			1

^ indicates that eye exam may occur at any time point between week 46-50.

*Eye exam at early discontinuation visit is required only if the initial dose of bNAbs has been received and may be completed within 16 weeks of the bNAbs dose. This replaces the eye exam scheduled between Weeks 46-50. Only one follow-up eye exam is required assuming no other triggers for eye exam are warranted after Week 24.

Δ indicates the End Study visit will be 52 weeks after the last product administration. For participants who receive the final investigational product at Week 34, the End Study visit is Week 86. If participants discontinue treatment early, the End Study visit will be 52 weeks after the last IP is received. The window for the End Study visit will be +/- 2 weeks. The End Study visit does not equate to the final visit of the study.

The schedule during the ATI period will be defined by clinical course and the characteristics of any virus rebound. ART will be initiated for clinical events, sustained drop in CD4+T cell counts or sustained increased in viremia. Prior to these events, subjects will be followed weekly for 24 weeks and then every two weeks. Once ART is initiated, subjects will be followed at the time of ART restart, and at 4, 8, and 24 weeks after restart.

Note: In cases in which clinical laboratory testing is performed remotely (for example, if trying to minimize in-person visits during the ATI for individuals living outside of San Francisco during the SARS-CoV-2 pandemic), storage of specimens may be deferred when samples are not collected on site.

11 EXPERIENCE REPORTING AND DOCUMENTATION

11.1 Adverse Events

An adverse event (AE) is any untoward medical occurrence in a clinical investigation of a patient administered a pharmaceutical product and that does not necessarily have a causal relationship with the treatment. An AE is therefore any unfavorable and unintended sign (including an abnormal laboratory finding), symptom or disease temporally associated with the administration of an investigational product, whether or not related to that investigational product. An unexpected AE is one of a type not identified in nature, severity, or frequency in the current Investigator's Brochure or of greater severity or frequency than expected based on the information in the Investigator's Brochure.

The Investigator will probe, via discussion with the subject, for the occurrence of AEs during each subject visit and record the information in the site's source documents. Adverse events will be recorded in the patient CRF. Adverse events will be described by duration (start and stop dates and times), severity, outcome, treatment and relation to study drug, or if unrelated, the cause.

Participants will also be asked to complete a patient diary to collect local injection site reactions and systemic reactogenicity events for seven days following each study product administration. These include but are not limited to local injection site reactions and systemic events including fever, chills, headache, fatigue or malaise, myalgias, arthralgias, nausea, and vomiting.

11.2 AE Severity

The Division of AIDS (DAIDS) Table for Grading the Severity of Adult and Pediatric Adverse Events, Corrected Version 2.1 (July 2017) (http://rsc.tech-res.com/clinical-research-sites/safety-reporting/daids-grading-tables) should be used to assess and grade AE severity, including laboratory abnormalities judged to be clinically significant. If the experience is not specifically identified in the DAIDS grading table, the guidelines shown in table 1 below should be used to grade severity. All deaths related to an AE are to be classified as grade 5. It should be pointed out that the term "severe" is a measure of intensity and that a severe AE is not necessarily serious.

Table 1. AE Severity Grading

Severity (Toxicity Grade)	Description
Mild (1)	Mild symptoms causing no or minimal interference with usual social and functional activities with intervention not indicated. The subject may be aware of the sign or symptom but tolerates it reasonably well.
Moderate (2)	Moderate symptoms causing greater than minimal interference with usual social and functional activities with intervention indicated.
Severe (3)	Severe symptoms causing inability to perform usual social and functional activities with intervention or hospitalization indicated.
Potentially Life-threatening (4)	Potentially life-threatening symptoms causing inability to perform basic self-care functions with intervention indicated to prevent permanent impairment, persistent disability, or death.

11.3 AE Relationship to Study Drug

The relationship of an AE to the study drug should be assessed using the following the guidelines in Table 2.

Table 2. AE Relationship to Study Drug

Relationship to Drug	Comment
Related	There is a reasonable possibility that the AE may be related to the study agent(s).
Not related	There is not a reasonable possibility that the AE is related to the study agent(s).

11.4 Serious Adverse Events (SAE)

An SAE is defined as any AE occurring at any dose that results in any of the following outcomes:

- Death during the period of surveillance defined by the protocol
- A life-threatening adverse experience
- Inpatient hospitalization or prolongation of existing hospitalization
- A persistent or significant disability/incapacity
- A congenital anomaly/birth defect

Other important medical events may also be considered an SAE when, based on appropriate medical judgment, they jeopardize the subject or require intervention to prevent one of the outcomes listed.

11.5 Serious Adverse Event Reporting

All SAEs that are related to study drug or device will be reported. In accordance with the standard operating procedures and policies of the local Institutional Review Board (IRB) and the U.S. Food and Drug Administration (FDA) per the reporting requirements specified by 21 CFR 312.32. Details of all related SAEs will be sent to the SMC no later than 3 reporting days after the investigators become aware of the event.

The PI and his team have extensive clinical trials experience and have in place a robust program for collecting, analyzing and reporting all adverse events.

Medical monitoring of safety data, including review of all adverse events, will be performed by the UCSF Principal Investigator. This will include regular review of safety data according to a medical monitoring plan, which will also include a process for informing the FDA, IRB and collaborating institutions. All collaborators who are responsible for safety reporting on products used in this trial will be informed within 72 hours of any SAE becoming known to the UCSF PI and sent a copy of an SAE report form within 15 days.

A Protocol Safety Review Team (PSRT) will be formed for weekly review of safety data, including ad hoc review of potential study pause criteria. The PSRT will include the protocol PI, co-I, SMC chair, potentially medical representatives from other product developers. Weekly review will ensure that safety risks other than SAEs are reviewed and communicated.

UCSF will be responsible for reporting required SAEs and safety risks to appropriate institutions. Study sites will document all SAEs that occur (whether or not related to study drug or device). The collection period for all SAEs will begin after informed consent is obtained and end after procedures for the final study visit have been completed.

11.6 Unanticipated (Serious) Adverse Device Effect (UADE)

A UADE is any serious adverse effect on health or safety or any life-threatening problem or death caused by, or associated with, a device, if that effect, problem, or death was not previously identified in nature, severity, or degree of incidence in the investigational plan or application (including a supplementary plan or application), or any other unanticipated serious problem associated with a device that relates to the rights, safety, or welfare of subjects. Per the definition above, a UADE is a type of SAE that requires expedited reporting on the part of the Sponsor.

The study team will assess each device related SAE to determine if anticipated based on prior identification within the investigational plan.

11.7 Medically attended adverse events

We will be using immunotherapy to induce a strong and sustained anti-HIV immune response. This approach theoretically might be associated with the induction of autoimmune or auto-inflammatory diseases. We will collect, analyze and report all data related to any medically attended adverse events (MAAEs), including potentially immune-mediated medical conditions (PIMMCs, Appendix A) through week 86 (52 weeks after the last bNAb administration). The potential relatedness of any MAAE to the

investigational product will be assessed. Any potentially immune-mediated medical condition will be categorized as unexpected.

11.8 Local reactogenicity

Based on extensive experience with the DNA and MVA vaccines, the only expected adverse events in Stages 1 to 3 will be local discomfort and pain, which is always transient. Study subjects will be directly observed by study personnel for 30 minutes after each injection for immediate reactions. The occurrence and severity of any AE during this period or the lack of same will be recorded on the appropriate CRF using the DAIDS Table for Grading the Severity of Adult and Pediatric Adverse Events, Corrected Version 2.1, July 2017, Section on Site Reactions to Injections and Infusions (see below).

Local reactions of mild (Grade 1) or moderate (Grade 2) severity will usually resolve spontaneously. If needed, they may be managed with local application of cold packs, oral acetaminophen, oral nonsteroidal anti-inflammatory agents, or a combination of these measures as appropriate.

The protocol team will be notified of all Grade 3 or 4 local toxicities within 48 hours of occurrence for consideration of modification or discontinuation of immunizations. For example, if a subject develops an intolerable local swelling (Grade 3 on the pain/tenderness scale) during the 72 hours after immunization, the protocol team should be contacted.

Systemic symptoms will be characterized using the DAIDS Table for Grading the Severity of Adult and Pediatric Adverse Events, Corrected Version 2.1 (July 2017) (http://rsc.tech-res.com/clinical-research-sites/safety-reporting/daids-grading-tables).

All adverse events will be summarized, tabulated and reported as described in Section 15.2.

Table. DAIDS Grading Table for Site Reactions to Injections

PARAMETER	GRADE 1 MILD	GRADE 2 MODERATE	GRADE 3 SEVERE	GRADE 4 POTENTIALLY LIFE-THREATENING
**Injection Site Pain or Tenderness** *Report only one*	Pain or tenderness causing no or minimal limitation of use of limb	Pain or tenderness causing greater than minimal limitation of use of limb	Pain or tenderness causing inability to perform usual social & functional activities	Pain or tenderness causing inability to perform basic self-care function OR Hospitalization indicated
**Injection Site Erythema or Redness**[12] *Report only one* *> 15 years of age*	2.5 to < 5 cm in diameter OR 6.25 to < 25 cm^2 surface area AND Symptoms causing no or minimal interference with usual social & functional activities	≥ 5 to < 10 cm in diameter OR ≥ 25 to < 100 cm^2 surface area OR Symptoms causing greater than minimal interference with usual social & functional activities	≥ 10 cm in diameter OR ≥ 100 cm^2 surface area OR Ulceration OR Secondary infection OR Phlebitis OR Sterile abscess OR Drainage OR Symptoms causing inability to perform usual social & functional activities	Potentially life-threatening consequences (e.g., abscess, exfoliative dermatitis, necrosis involving dermis or deeper tissue)
≤ 15 years of age	≤ 2.5 cm in diameter	> 2.5 cm in diameter with < 50% surface area of the extremity segment involved (e.g., upper arm or thigh)	≥ 50% surface area of the extremity segment involved (e.g., upper arm or thigh) OR Ulceration OR Secondary infection OR Phlebitis OR Sterile abscess OR Drainage	Potentially life-threatening consequences (e.g., abscess, exfoliative dermatitis, necrosis involving dermis or deeper tissue)
**Injection Site Induration or Swelling** *Report only one* *> 15 years of age*	Same as for **Injection Site Erythema or Redness,** *> 15 years of age*	Same as for **Injection Site Erythema or Redness,** *> 15 years of age*	Same as for **Injection Site Erythema or Redness,** *> 15 years of age*	Same as for **Injection Site Erythema or Redness,** *> 15 years of age*
≤ 15 years of age	Same as for **Injection Site Erythema or Redness,** *≤ 15 years of age*	Same as for **Injection Site Erythema or Redness,** *≤ 15 years of age*	Same as for **Injection Site Erythema or Redness,** *≤ 15 years of age*	Same as for **Injection Site Erythema or Redness,** *≤ 15 years of age*
**Injection Site Pruritus**	Itching localized to the injection site that is relieved spontaneously or in < 48 hours of treatment	Itching beyond the injection site that is not generalized OR Itching localized to the injection site requiring ≥ 48 hours treatment	Generalized itching causing inability to perform usual social & functional activities	NA

11.9 Acute Retroviral Syndrome

Individuals who interrupt ART are at risk of developing an acute retroviral syndrome, which can lead to hospitalization and even death. We will follow subjects carefully and resume therapy for any clinical presentation consistent with the case definition, as described below.

Case definition must include all of the following:

- Occurs within 30 days after documented rise in plasma HIV-1 RNA of ≥ 1 log10 or to ≥ 1,000 copies/mL
- Documented fever (temperature >38.5°C)
- At least two major criteria
- At least two minor criteria
- No other cause for signs and symptoms identified

Major criteria (must be present for >7 days):

- Pharyngitis
- Fatigue
- Morbilliform rash
- Myalgia/arthralgia
- Lymphadenopathy
- Subjective fever as reported by subject (may be intermittent)

Minor criteria (must be present for >7 days):

- Headache
- Nausea/vomiting
- Diarrhea
- Mucocutaneous ulceration (oral, genital, and/or anorectal)
- Meningismus/aseptic meningitis
- Night sweats
- Thrombocytopenia (platelets <150,000)
- Leukopenia (WBC <4,000)

- Malaise
- Abdominal pain
- Weight loss (loss of >5% of body weight)

11.10 Procedures for Documenting Pregnancy During the Trial

Subjects who are pregnant or expect to become pregnant during the course of the trial will be excluded from participation in the trial. Should a subject become pregnant after enrolling in the trial, she will not be given any further treatments with study drug or device. A Pregnancy Form will be completed by the Investigator and submitted to the study team within 24 hours after learning of the pregnancy. The Investigator will also report this event to the IRB within 24 hours of becoming aware of the pregnancy. Sites must request the subject's permission to query pregnancy outcome and follow each subject to determine the outcome of the pregnancy. When permission is received, subjects will continue to be followed for safety assessments to trial discharge per protocol. Results will be summarized in the clinical study report (CSR).

Subjects who become pregnant at any point during the trial will continue to be followed for safety assessments without receiving further study drug or device. Procedures that are contraindicated during pregnancy, including additional treatments, must not be performed. Investigators should use clinical judgment regarding subsequent trial-related blood collection based on the presence or absence of anemia in each subject.

All pregnancies that occur from the time of first screening procedure through the follow-up visits must be reported. The Investigator will monitor the subject and follow the outcome of the pregnancy. If the end of the pregnancy occurs after the trial has been completed, the outcome will be reported directly to the trial team.

Male subjects will be instructed through the Informed Consent Form to immediately inform the Investigator if their partner becomes pregnant until the end of follow-up period. A Pregnancy Form will be completed by the Investigator and submitted to the study team within 24 hours after learning of the pregnancy. Attempts will be made to collect and report details of the course and outcome of any pregnancy in the partner of a male subject exposed to study drug or device. The pregnant partner will need to sign an Authorization for Use and Disclosure of Pregnancy Health Information to allow for follow up on her pregnancy. Once the authorization has been signed, the Investigator will update the Pregnancy Form with additional information on the course and outcome of the pregnancy. An Investigator who is contacted by the male subject or his pregnant partner may provide information on the risks of the pregnancy and the possible effects on the fetus, to support an informed decision in cooperation with the treating physician and/or obstetrician.

11.11 Reporting of Device Related Complaints or Deficiencies

A product complaint/device deficiency is defined as any written, electronic, or oral communication that alleges deficiencies or inadequacies of the device or components related to the identity, quality, durability,

reliability, safety, effectiveness, or performance of the electroporation device or components after it is released for distribution within the clinical investigation.

Device deficiencies include malfunctions, use errors and inadequate labeling. A malfunction is defined as the failure of a device to meet its performance specifications or otherwise perform as intended. The intended performance of a device refers to the intended use for which the device is labeled or marketed.

Any problems experienced during the treatment procedure including potential malfunctions of the device, error messages displayed on the device screen following treatment or errors that occur during the treatment procedure must be reported to the Sponsor or designee immediately for evaluation.

12 CLINICAL MANAGEMENT ISSUES

Subjects may be offered topical anesthetic (e.g. EMLA or equivalent), to prevent significant discomfort from the electroporation procedures (Weeks 0, 4 and 12). If a topical anesthetic is used, an approximately 1.5 cm diameter amount will be applied with occlusion to the site of injection ~30 minutes prior to treatment. Subjects may be offered a non-narcotic analgesic (e.g. ibuprofen, ketorolac) after injection/EP.

Vaccination may be deferred for clinical or safety concerns such as febrile intercurrent illness at the discretion of the study investigator. Management of an individual participant should be determined by the study investigator with consultation with the study team.

13 DISCONTINUATION AND REPLACEMENT OF SUBJECTS

13.1 Early Discontinuation

A subject may be discontinued from study treatment at any time if the subject or the study team feels that it is not in the subject's best interest to continue. The following is a list of possible reasons for study treatment discontinuation:

- Subject withdrawal of consent
- Subject is not compliant with study procedures
- Adverse event that in the opinion of the investigator would be in the best interest of the subject to discontinue study treatment
- Protocol violation requiring discontinuation of study treatment
- Lost to follow-up
- Sponsor request for early termination of study
- Positive pregnancy test (females)
- Request for withdrawal by the subject's regular doctor

If a subject is withdrawn from treatment due to an adverse event, the subject will be followed and treated by the Investigator until the abnormal parameter or symptom has resolved or stabilized.

All subjects who discontinue study treatment should come in for an early discontinuation visit as soon as possible and then should be encouraged to complete all remaining scheduled visits and procedures as needed.

All subjects are free to withdraw from participation at any time, for any reason, specified or unspecified, and without prejudice.

Reasonable attempts will be made by the investigator to provide a reason for subject withdrawals. The reason for the subject's withdrawal from the study will be specified in the subject's source documents.

13.2 Withdrawal of Subjects from the Study

A subject may be withdrawn from the study at any time if the subject, the investigator, or the Sponsor feels that it is not in the subject's best interest to continue.

All subjects are free to withdraw from participation at any time, for any reason, specified or unspecified, and without prejudice.

Reasonable attempts will be made by the investigator to provide a reason for subject withdrawals. The reason for the subject's withdrawal from the study will be specified in the subject's source documents. As noted above, subjects who discontinue study treatment early should have an early discontinuation visit.

13.3 Replacement of Subjects

Subjects who withdraw from the study or discontinue the study following study entry (day of first study IP administration, D0) will not be replaced.

14 PROTOCOL VIOLATIONS

A protocol violation occurs when the subject or investigator, fails to adhere to significant protocol requirements affecting the inclusion, exclusion, subject safety and primary endpoint criteria. Protocol violations for this study include, but are not limited to, the following:

- Failure to meet inclusion/exclusion criteria
- Use of a prohibited concomitant medication
- Failure to comply with Good Clinical Practice (GCP) guidelines will also result in a protocol violation.

When a protocol violation occurs, it will be discussed with the study team and a Protocol Violation Form detailing the violation will be generated. A copy of the form will be filed in the site's regulatory binder.

15 STATISTICAL METHODS AND CONSIDERATIONS

We will perform a phase I/II open-label, single-arm, proof-of-concept study to evaluate the safety, immunologic, virologic and clinical effects of a novel combination approach aimed at achieving durable control of HIV in the absence of therapy. We will study 20 HIV-infected participants receiving effective ART who have HIV-1 RNA below the level of quantificationand CD4+ T-cell counts ≥500 cells/mm3. Each participant will receive the same regimen in an open-label manner followed by a carefully monitored ATI. Accrual of the targeted 20 participants is anticipated to take 8-12 months.

We will follow standard good statistical practices, including examination of summary statistics, assessment of model assumptions, examination and presentation of graphical depictions of the data, assessing the impact of influential data points, and interpretation that reflects the quantitative information provided by estimated effects and their confidence intervals, rather than an exclusive focus on whether or not $p<0.05$.

15.1 Data Sets Analyzed

All eligible patients who are enrolled into the study and who initiate therapy will be included in the safety and efficacy analysis. Analyses will be conducted by both intention-to-treat (primary) and per protocol approaches. Low rates of non-adherence to study treatment and loss-to-follow-up are anticipated.

15.2 Safety Analysis

All subjects will be followed for possible AEs throughout their involvement in the study. Routine blood work will be performed on a regular basis. AEs will be graded according to the DAIDS Table for Grading the Severity of Adults and Pediatric Adverse Events, Corrected Version 2.1, July 2017 (http://rsc.tech-res.com/clinical-research-sites/safety-reporting/daids-grading-tables). The Principal Investigator will review these data daily, assess their degree of severity, and make a relationship assessment to study agent/intervention. A study data coordinator will produce administrative reports after completion of each cohort describing study progress including the following: (1) accrual, (2) demographics, (3) study subject status, and (4) number and type of serious AEs. The Safety Monitoring Committee (SMC) will review study progress, efficacy data, all interim and total AEs, and unanticipated problems involving risk to participants. Reviews will be communicated to the UCSF Institutional Review Boards, study sponsor, and/or federal agencies, as appropriate. The study will be discontinued if the SMC determines that it is in the best interest of the subjects.

Safety will be assessed by tabulating the primary safety endpoint, specific SAE's, SAE's judged to be treatment-related, and the occurrence of any one or more SAE, along with exact 95% confidence intervals for the proportions of participants experiencing each event. Trends in the occurrence of safety events will be estimated by mixed effects logistic regression models that have study week as a linear predictor.

15.3 Safety Endpoints

Primary safety endpoints: For the safety analysis, all subjects who have been exposed to study treatment will be included. Given the single arm nature of the trial, baseline and post-therapy time points will be used to assess contribution of study drug to potential AE or other toxicities.

The primary safety endpoint will be the occurrence of a new Grade ≥3 AE, including signs/symptoms, lab toxicity or clinical event, that is definitely, probably or possibly related to study treatment. With regard to relatedness to a specific intervention (CE/gag DNA vaccine, IL-12 DNA adjuvant, MVA boost, VRC07-523LS, 10-1074, lefitolimod or the ATI), attempts will be made to assign relatedness, with a window of up to 28 days since exposure considered as making any event at least possibly related.

Other safety endpoints. Other safety endpoints will be (1) the occurrence of any unsolicited AEs for 28 days after administration of each study agent, (2) the occurrence of any SAE, MAAE, and PIMMC from the time of administration of the first study injection through 12 months after administration of the final study injection, (3) the occurrence of two consecutive measurements HIV RNA >200 copies/mL using conventional assays, (4) resumptions of ART after ATI and the events that trigger them, and 5) additional stage-specific endpoints as discussed in the following paragraph.

Given the multi-stage nature of this intervention, the types of expected adverse events will vary over time. With regard to the Stages 1 to 3 (prime-boost vaccination series), local reactogenicity and pain related to electroporation are expected to be common. Any Grade 2 or higher reactogenicity event for 7 days following administration of each study agent will be considered an endpoint. With regard to Stage 4 (bNAbs and lefitolimod), we will include analysis of ophthalmologic adverse events and elevations in direct bilirubin, as well as episodes compatible with cytokine release following bNAb administration. With regard to Stage 5 (ATI), we will consider as an endpoint (1) the frequency of confirmed declines (two consecutive measurements) in CD4+ T cell counts (> 50%), (2) the frequency of confirmed declines to below 350 cells/mm^3, and (3) any clinically defined episode of acute retroviral syndrome.

Counts and proportions of adverse events will be presented in frequency tables with 95% Clopper-Pearson Confidence Intervals. In addition, spaghetti plots of CD4 and $\log_{10}$(HIV RNA) by study week will be examined and provided to the study monitoring committee.

Note: As of Protocol Version 2.2, no further lefitolimod will be administered. Safety endpoints remain the same even without the administration of lefitolimod.

15.4 Primary Efficacy Endpoint: Post-treatment Control

All subjects will undergo a treatment interruption designed to evaluate the primary outcome of "post-treatment control" (PTC), using the definition we recently used as part of the CHAMP Cohort collaboration[132]. In this study, PTCs were defined as individuals who remained off ART for ≥24 weeks and maintained viral loads ≤400 copies/mL for at least 2/3 of the time points. Viral loads >400 HIV-1 RNA copies/mL were acceptable if the participant was subsequently able to suppress the viral load back to ≤400 HIV-1 RNA copies/mL and maintained virologic control for 24 weeks.

In our protocol, a combination of two bNAbs will be administered at the time ART is interrupted, and will likely have a persistent antiviral effect until levels wane to subtherapeutic levels, which we will assume to occur at about Week 12 of the ATI period. Also, we expect that in the post-ART and post-bNAb phase of the ATI that a period of acute viremia will occur, and that this could last for up several weeks. As described in Section 1.11, ART will be initiated if plasma HIV RNA levels remain consistently > 50,000 copies RNA/mL for four weeks, > 10,000 copies RNA/mL for six weeks, > 2000 copies RNA/mL for >

12 weeks, and > 400 copies RNA/mL > 24 weeks. In this scenario, post-treatment control will only be achieved once viremia falls below 400 copies RNA/mL and remains there for 24 weeks.

Success will hence be defined in two ways. First, subjects who fail to show any consistent rebound above 400 copies RNA/mL between Week 12 of the ATI (when bNAb levels wane) and Week 36 of the ATI will be considered as having achieved post-treatment control. Second, subjects who exhibit a rebound and eventually achieve 24 weeks of virus control will be considered as having achieved post-treatment control.

Failure will be defined based on the need to resume ART due to high-level viremia, dropping CD4+ T cell counts, or clinical events (see Section 1.11).

The proportion of individuals who achieve our study-defined PTC will be the primary outcome.

15.5 Secondary Other Efficacy Endpoints

Secondary efficacy endpoints: The magnitude of response at the follow-up will be presented based on appropriate measures of central tendency (mean or median) and variability (standard deviation or interquartile range) in the T cell responses in each treatment arm.

Breadth of response will be characterized by the proportion of participants with at least one additional epitope response at week 14 (2 weeks after last vaccination) compared to their baseline response. The observed proportion of participants with response in breadth so defined will be presented with 95% Clopper-Pearson Confidence Intervals.

Other efficacy endpoints: Descriptive analyses and inferences outlined for the Primary endpoints will be applied to the Secondary and the Other endpoints. Some measures, such as integrated DNA and cell-associated RNA, will likely be better modeled by negative binomial regression, as they tend to have a skewed distribution. In addition, it may be desirable to account for the amount of input to the assays, which is readily done in negative binomial models by inclusion of an "exposure" variable. In these cases, and also when linear regression or t-tests of logarithmically transformed measurements are appropriate, we will report relative effects (percentage or fold). Because nonparametric methods such as the Wilcoxon signed-rank test produce only p-values with no quantitative effect estimates, we will use these only as confirmatory analyses or when no quantitative analysis appears viable.

Statistical analyses for all aims will follow standard good statistical practices, including assessment of model assumptions, checking for influential observations, and interpreting results in light of estimated effects and their confidence intervals, in addition to p-values.

Trajectories of continuous measures evaluated longitudinally will be examined using mixed effects models with random slopes and/or intercepts, as appropriate. Similarly, categorical measures such as the number of "new" positive responses, the number of responses that remain negative, will be investigated in generalized mixed effects models. for many reservoir measures, we anticipate using negative binomial regression, because it can properly account for skewed distributions, observed values of zero, and varying amounts of input to the assays, while estimating relative effects (fold or percentage), which will be the most meaningful scale for many of the measurements. Whenever possible, we will also calculate and analyze a single summary measure over the entire treatment period for each person, e.g., the area under

the curve and over baseline for immunogenicity measures over time. in the cases where the main interest focuses on the final measurement versus pre-treatment, we will also employ simpler methods such as paired Student's *t*-tests when feasible.

15.6 Sample Size

The objective of this study is to characterize the safety and efficacy of a combination remission strategy. Given experience with each component of the regimen in the past, we do not expect to observe many significant adverse events.

Selecting the target population. We carefully considered the ideal target population for this combination trial to maximize the impact of the study. Several lines of evidence from observational studies and randomized controlled trials suggest that individuals starting ART early (e.g., within the first 6 months of infection) have reduced chronic immune activation, improved adaptive immune function, and a lower HIV reservoir size than individuals starting ART late (e.g., after 2 years of infection). For all these reasons, individuals treated early would be expected to have a more robust immunologic response to our immunotherapies and have a more manageable reservoir size to contain than those starting ART late. Indeed, the remission/cure outcomes noted in the non-human primate studies that provide the rationale for this study were all preformed in animals treated during the acute phase of the infection. Thus, to establish proof of principle that this therapeutic strategy can contain and/or eliminate reservoirs in the absence of therapy, we will primarily enroll individuals who started ART early.

We also recognize that there are practical limitations to enrolling a full 20 participants who started ART this early and recognize the value of testing this intervention in at least some individuals who started ART in later stages of infection to begin to address the potential generalizability of our therapeutic strategy in the vast majority of HIV-infected individuals who started ART late. We thus chose to focus on early ART participants for our primary analysis (and powering the study to detect clinically meaningful levels of post-treatment control) and include a small subset of chronically infected individuals for an exploratory analysis to develop preliminary data that might inform the next set of studies in chronically infected individuals should our strategy be successful in those who start early. Finally, we propose enrolling these two groups concurrently as awaiting the outcome in one subgroup before proceeding with the next would extend the length of the study from 2 years to up to 4 years, unnecessarily delaying scientific progress well beyond 2020.

Primary efficacy outcome. All subjects will undergo a treatment interruption designed to evaluate the primary outcome of "post-treatment control" (PTC). The proportion of individuals who achieve PTC will be tabulated and a 2-sided 95% confidence interval obtained by exact calculations for the binomial distribution. For those who initiated ART during acute infection, a literature review indicates that no more than 15% PTC would be expected without intervention. For those who initiated during chronic infection, no more than 5% PTC would be expected. These maximum expected rates will serve as the null hypothesis for our P-value calculations. Evaluation of the acute group will be the primary analysis, while evaluation of the chronic group will be exploratory and provide initial preliminary information on generalizability.

Using standard and innovative measures of immunogenicity, we will determine the capacity of our vaccine strategies to stimulate broad, functional T cell responses against novel HIV epitopes, and comprehensively

characterize the impact of the vaccine on several innate and adaptive immune parameters. The size of the active and latent reservoirs before, during and after vaccination will be measured.

Power calculations. For the acute group, observing 6 or more PTC among N=15 (40%) will result in $P<0.05$ versus the null hypothesis of 15%, and the study will have ≥80% power if the true chance of PTC with intervention is ≥47%.

For the chronic group, observing even one PTC (20%) would provide some indication of promise, and observing 2 PTC (40%) would produce $P<0.05$ versus the null hypothesis of 5%.

16 DATA COLLECTION, RETENTION AND MONITORING

16.1 Clinical trial infrastructure

This program is built on two well-established clinical research cohorts at UCSF. The **SCOPE** cohort follows chronically infected HIV-positive participants. Over 2000 HIV-infected and uninfected adults have been enrolled. The **Options** cohort follows participants from within 6 months of HIV infection. Nearly 1000 people have been enrolled since the cohort was established in the 1990s. These two cohorts were formally merged in 2017 and are now generally being followed in the SCOPE infrastructure. Our group has previously combined SCOPE and Options for a variety of studies, including a number of investigator-initiated clinical trials.

Over the past 15 years we used the SCOPE infrastructure to translate findings from the laboratory into the clinic. We have performed over 20 investigator-initiated studies and have led a number of small, intensive industry-sponsored studies. All studies were fully enrolled and retention has been outstanding. Several studies have been successfully monitored and no problems have emerged.

We have completed without incident studies that evaluated structured treatment interruptions (NCT00187551)[156-159], growth hormone (NCT00071240)[160], atorvastatin[161], valganciclovir (NCT00264290)[162], disulfiram (NCT01286259 and NCT01944371)[163,164], mesalamine (NCT01090102)[165] and lisinopril (a potential anti-fibrotic drug, NCT01535235)[166]. We designed and lead the first randomized study of CAR-T cell therapy[167]. We investigated the impact of maraviroc (NCT00735072) or raltegravir (NCT00631449) intensification of existing antiretroviral therapy[168-171] and performed the first prospective treatment study of HIV controllers (NCT01025427)[172]. We recently completed a study of everolimus in HIV-infected transplant recipients (NCT02429869) and an exploratory study of fecal microbial transplantation (NCT02256592, IND 19926). In collaboration with Dr. Priscilla Hsue, we performed a pilot study of canakinumab and are now doing a larger randomized study (NCT02272946). We are also supporting Dr. Hsue's work with PCSK9 inhibitors (NCT02524106 and NCT03207945). We designed a unique study of a botanical (Kansui), which has promise as a latency reversing agent (NCT02531295); we obtained an IND and are now completing a pilot study in SIV-infected macaques. We designed and are about to implement at randomized study of a therapeutic vaccine (PENNVAX, Inovio). This NIAID-sponsored study has been intensively evaluated by multiple committees and two on-site evaluations. Finally, we are leading a number of studies assessing radiolabeled markers of treatment distribution (NCT03174977), immune function and the reservoir.

The PI has worked closely with industry partners as well. For example, we helped design and are leading a study to GS-9620, a TLR-7 agonist being developed by Gilead (NCT03060447).

The PI has also worked with NIH-sponsored networks to translate findings from SCOPE to phase II testing and to address questions that require multiple sites. Based on our work with maraviroc intensification, we helped design and implement a multi-site NIAID-sponsored randomized study of this drug in organ transplantation (NCT02741323). Preliminary data from our group has been used to help launch similar studies within the ACTG; we co-lead efforts to study methotrexate (A5314, NCT01949116), ruxolitinib (JAK/STAT inhibitor; A5336) and sirolimus (A5337; NCT02440789). Finally, we participated in a limited center investigator-initiated study of a therapeutic vaccine (NCT00976404)[173].

Many of our current generation of studies involve interrupting discrete immunologic pathways that could plausibly affect size and distribution of the reservoir. All of our studies have had a strong basic science component and most require intensive tissue sampling. We have a robust network of collaborators and all of the needed protocols to access, process and distribute biologic specimens to characterize how our interventions affect immune responses and the reservoir.

16.2 Data Collection Instruments

Participants complete interviewer-administered questionnaires that includes a series of items on sociodemographic characteristics, ART usage and adherence, recreational drug use, and major clinical co-morbidities. Questionnaire-derived data are entered into the web-based Research Electronic Data Capture (REDCap) electronic data capture system, which has emerged as an international standard for clinical research, and ultimately are housed in a professionally maintained Microsoft SQL server-based database. In addition to the standard range and consistency checks available in REDCap (described below), an SQL-based application performs further custom cross-form logic checks, which the original field interviewers are asked to resolve. The goal is to optimize data completeness and accuracy. Data collected from outside sources, such as participating laboratories, are fully integrated into the study database. The data management environment is HIPPA and FDA compliant. There is daily and off-site backup.

16.3 Research Electronic Data Capture (REDcap)

As described online (https://myresearch.ucsf.edu/redcap-faqs) REDCap was designed to provide medical researchers with a professionally managed, secure, web based, HIPAA compliant environment for building and managing web-based projects. REDCap is hosted at the UCSF Medical Center data center (ECDC) and is housed in a locked and guarded data center. Entrance to the data center requires triple factor authentication: use of a card key to unlock the data center door, biometric authentication and a second card key lock secures the cage that the servers reside within. The security of the data center by a security camera system. REDCap servers are guarded by multiple firewall and intrusion detection systems. All electronic connections to the REDCap environment are encrypted. The REDCap production system is comprised of a web server front-end and a MySQL database server back-end. The web server resides in a demilitarized zone to ensure that survey participants are able to access REDCap surveys from any device connected to the Internet. The MySQL server back-end resides in the protected subnet that is guarded by UCSF maintained firewalls. The data stored in the REDCap MySQL database server can be accessed by the REDCap end users by logging into https://redcap.ucsf.edu and opening the REDCap project(s) that they have been granted access to by the owners of the projects. Only ITS system administrators are authorized to access the back-end database server directly by logging into the virtual private network for the database server resides in.

A complete data dump is done twice a day. One completes at approximately 5:15 pm Pacific time each day and another completes at approximately 1:15 am Pacific time each day.

16.4 Biologic specimen collection, processing and storage

UCSF Core Immunology Laboratory (CIL). The CIL is a CFAR-supported, GCLP-compliant laboratory that participates in internal and external proficiency testing programs through the National Institutes of Health, Division of AIDS. The laboratory is a complete end-to-end laboratory in that it is involved in

project development, sample processing, assay development, data generation (i.e. flow cytometry, ELISAs, cell sorting), data analysis and reporting, as well as manuscript preparation. To successfully accomplish this spectrum of work, the laboratory is staffed with a PhD trained immunologist (Technical Director) as well as an Operational Manager with a Master's degree in immunology. CIL operates 2 BD LSR-II analyzers and a BSL2* BD ARIA II instrument for sorting of infectious samples. CIL operates through a UCSF recharge system and is monitored by the UCSF budget office for financial compliance.

CIL provides investigators with access to specimen processing including blood, leukapheresis, gut tissue biopsies, cerebral spinal fluid, lymph node dissections as well as lymph node fine needle aspirates. CIL was established in 1995 and has worked closely with the study coordinators of SCOPE and Options for over two decades. The CIL Director and Operations Manager are in daily contact with SCOPE staff and a monthly in person meeting occurs between SCOPE study coordinators and the CIL management team to discuss ongoing study coordination. Through frequent communication and the close proximity on campus, the clinical and CIL teams are able to coordinate the efficient transfer of study samples for specimen processing. All processes and transfers of biologic specimens are tracked with verifiable chain-of-custody using a web-based version of Frontier Science Foundation's Laboratory Data Management System (LDMS) software. Each sample receives an accession number for data entry and tracking purposes. The ASB accession number will be unique specimen accession number that links the specimen to the subject study number, visit number, diagnosis or study name, collection date, specimen type, location of the specimen within CIL's freezers (box number and sample coordinates within each box), amount, and final disposition of the specimen. This also permits samples to be sent to the UCSF AIDS Specimen Bank for long-term storage

Blood and tissue specimens will be collected at the clinic site and transported at appropriate temperatures to the appropriate site within four hours: to either the UCSF Core Immunology Laboratory (for short-term storage or immediate specimen analysis) or the UCSF AIDS Specimen Bank for long-term storage. CIL maintains a close working relationship with the UCSF AIDS specimen and frequently ships samples between the laboratories for assay and storage purposes.

UCSF AIDS Specimen Bank. We will utilize the AIDS Specimen Bank (ASB) to process and store long-term all blood specimens. The ASB was established in 1982 with the mission of providing an infrastructure, service, and expertise for the collection, processing, storage, and distribution of human patient and research subject clinical specimens to be collected by UCSF and affiliated HIV investigators. The ASB provides service to many clinical studies based at UCSF including studies of HIV transplantation, the NCI-funded AIDS Cancer & Specimen Resource, and the Women's Interagency HIV Study.

Upon receipt at the ASB, all specimens will be double checked against the accompanying ASB deposit form. After the ASB staff checks the paperwork and the specimens, each specimen will receive a Specimen Bank accession number for data entry and tracking purposes. The ASB accession number will be unique to that particular specimen. The accession number links the specimen to the subject study number, visit number, diagnosis or study name, collection date, specimen type, location of the specimen in the ASB's freezers (box number), amount, and final disposition of the specimen. After the data have been entered, labels are made for the specimens and an aliquot sheet (worksheet) will be printed out. Cryovials are

labeled with computer-generated labels. The ASB staff member will take the aliquot sheet and compare it with the accompanying UCSF deposit form for accuracy. Information about specimens is entered into a relational database that enables efficient tracking and retrieval of specimens when needed.

The specimens and the aliquot sheet are taken to a P2 room that contains a Class II laminar flow hood, where all processing takes place. PBMC will be separated using Ficoll separation according to the ASB's laboratory protocols. To ensure cell optimal viability and recovery of PBMC, the data manager will select specimens from this study in which there are a large number of cells, and one member of the ASB staff will check for cell viability on a monthly basis. To assess variability and reproducibility in processing, two technicians are chosen at random to perform cell separations, and a comparison is made on their counts and viability.

Specimens will be stored in ultra-low freezers in which temperature is monitored by a programmable scanning alarm system wired into the university's telephone system. The system is programmed to sound an audible alarm for a few minutes and then to telephone the ASB staff (at work and home) alerting them of a malfunctioning freezer or power outage on campus.

The ASB uses Microsoft Access as its database to track its entire inventory. The majority of specimens received in the AIDS Specimen Bank are coded, thus no names are given and no linkages are made between the bank's database and the patient's identity. Specimens are tracked by specimen type, investigator's name, study name, diagnosis, and other information that the clinician or investigator provides the bank. The specimens are assigned a unique identifying number, the ASB accession number; this number links each specimen to the patient study number, visit number, diagnosis or study name, collection date, specimen type, location of the specimen in the bank's freezers (box number), amount, and final disposition of the specimen.

16.5 Data Management Procedures

The Investigator is responsible for all information collected on subjects enrolled in this study. All data collected during the course of this study must be reviewed and verified for completeness and accuracy by the Investigator's study team.

To maintain confidentiality, all laboratory specimens, evaluation forms, reports and other records will be identified by a coded number. All participants who consent and screen for participation in the study will be assigned a unique 4-digit SCOPE ID that will serve as their participant ID (PID). UCSF participants who are already SCOPE participants will be assigned their existing SCOPE ID. New participants will be assigned new SCOPE IDs.

All study records will be kept in a locked file cabinet. Clinical information is not released without written permission of the participant, except as necessary for monitoring by the FDA. The Investigator must also comply with all applicable privacy regulations (e.g., Health Insurance Portability and Accountability Act of 1996, EU Data Protection Directive 2016/679).

Coordinated data management and analysis will enhance efficiency and facilitate cross-project collaboration with strong quality assurance. Data management, specimen storage, and data analysis is directed by Dr. Jeffrey Martin at the UCSF Data Coordinating Center in the Department of Epidemiology and Biostatistics. The Data Management group will be responsible for data processing, in accordance with

procedural documentation. Data instrument locks will occur once quality assurance procedures have been completed.

All procedures for the handling and analysis of data will be conducted using good computing practices meeting FDA guidelines for the handling and analysis of data for clinical trials. Our REDCap system is compliant and data will be managed as follows:

- Data is recorded/placed in a participant's chart by the attending investigator or study coordinator during/after each visit.
- The source documentation is delivered to the data manager or designee.
- The data manager or designee performs a quality control check and enters data into the REDCap database.
- Once the quality control check is accomplished and accuracy of data is confirmed, the entered data will be locked into REDCap by the data manager or designee.
- Only the data manager or PI or designee of the study has the privilege of unlocking the data in REDCap if needed.
- The study PI will review the REDCap database and sign off on its accuracy upon study completion.

16.6 Data Quality Control and Reporting

After data have been entered into the study database, a system of computerized data validation checks will be implemented and applied to the database on a regular basis.

All data will be managed by the UCSF Data Coordinating Center in the Department of Epidemiology and Biostatistics. Issues related to data management, specimen storage, and data analysis will be directed by Dr. Jeffrey Martin, who co-directs the SCOPE cohort with Dr. Deeks. Study participants will complete an interviewer-administered questionnaire modified to support the unique aspects of our proposed study (including collection of detailed information regarding sexual activities in both the study participant, and his or her partners). The standard system is compliant with all Federal Government confidentiality guidelines.

16.7 Archival of Data

The database is safeguarded against unauthorized access by established security procedures; appropriate backup copies of the database and related software files will be maintained. Databases are backed up by the database administrator in conjunction with any updates or changes to the database.

At critical junctures of the protocol (e.g., production of interim reports and final reports), data for analysis is locked and cleaned per established procedures.

16.8 Availability and Retention of Investigational Records

The Investigator must make study data accessible to the monitor, other authorized representatives of the Sponsor (or designee), IRB/IEC, and Regulatory Agency (e.g., FDA) inspectors upon request. A file for each subject must be maintained that includes the signed Informed Consent, HIPAA Authorization Form and copies of all source documentation related to that subject. The Investigator must ensure the reliability and availability of source documents from which the information on the CRF was derived.

All study documents (patient files, signed informed consent forms, copies of CRFs, Study File Notebook, etc.) must be kept secured for a period of two years following marketing of the investigational product or for two years after centers have been notified that the IND has been discontinued. There may be other circumstances for which the Sponsor is required to maintain study records and, therefore, the Sponsor should be contacted prior to removing study records for any reason.

16.9 Regulatory and Data Monitoring

Clinical site monitoring visits related to regulatory, data, and pharmacy procedures will be conducted according to the U.S. CFR Title 21 Parts 50, 56, and 312 and ICH Guidelines for GCP (E6). By signing this protocol, the Investigator grants permission to the Sponsor (UCSF) and appropriate regulatory authorities to conduct on-site monitoring and/or auditing of all appropriate study documentation.

The clinical trial will be monitored by representatives from the study team trained in reviewing the operations of the study. The team will adapt existing protocols that have implemented for other investigator-initiated studies. Monitoring will require on site visits scheduled based on number of cumulative participant visits.

Study monitoring will be performed according to a mutually agreed risk-based monitoring plan which will include verification of subject eligibility, consent forms and consenting process, review of all adverse events, and general compliance to the protocol and GCP.

The monitoring team will generate follow-up questions and queries and work with Ms. Hoh and the UCSF team to address any concerns. Monitoring reports and documents noting close out of all follow up items will be provided to sponsors and collaborators.

16.10 Safety Monitoring Committee (SMC)

A Study Monitoring Committee (SMC) will monitor study progress and participant safety, with one member of the SMC designated to serve as an Independent Safety Monitor (ISM). Approximately 3 months after enrollment of the first participant and then every 3 months thereafter, the SMC will meet and review accrual (including screening and enrollment), AE summaries, including all reported Grade ≥3 AEs, retention of participants including off-study rates, and longitudinal summaries of HIV-1 RNA by commercial assay and CD4+ T-cell count. In addition, the SMC will review sample collection and availability summaries for viable PBMCs and plasma.

In addition to the regularly scheduled reviews, a safety review conducted by the SMC for any of the following criteria:

- Any ophthalmologic adverse event of any severity grade, pending examination by an ophthalmologist and review by the SMC (bNAbs will be paused).
- Any abnormal elevation in direct bilirubin, confirmed on repeat testing, pending evaluation by a hepatologist.
- For participants entering Stage 5 under Protocol version 2.2, if any grade 2 or higher LFT elevation (ALT, AST, or total bilirubin) is detected.

- Two or more participants have experienced a grade 3 event that is deemed possibly, probably or definitely related to study treatment
- One or more participants have experienced a grade 4 event that is deemed possibly, probably or definitely related to study treatment
- One or more participants has experienced an SAE that is deemed possibly, probably or definitely related to study treatment
- Any death that occurs on study that is deemed possibly, probably or definitely related to study treatment should continue to be a criterion for expedited SMC review.

If the above criteria are met, the PI will request a review by the SMC (or the SMC chair if other SMC members cannot be convened), to be held within 3 business days of learning of the event.

Whenever any of the events above occurs (with one exception, noted below), enrollment into the study will be paused until the SMC review has taken place and a determination has been made that enrollment can resume. In addition, administration of all study products will be paused until a course of action is recommended by the SMC. The only exception is an ophthalmologic AE, which will result in a pause of the bNAb administrations; participants cannot enter Week 24 or Week 34 of the study in such cases, but other study IP administrations can continue as these administrations are not expected to affect the safety of the participants. The SMC will recommend, based on the results of the review, whether the study can proceed as planned, proceed with modifications, or should be discontinued.

The SMC may also be convened if a reason is identified by the study team or study statistician.

The SMC will review progress towards pre-specified benchmarks of enrollment and retention of subjects, completion of study procedures, and collection of viable samples. If progress towards any benchmark is not adequate, as determined by the SMC, the SMC will recommend protocol modification if necessary.

16.11 Subject Confidentiality

In order to maintain subject confidentiality, only a four-digit subject number will identify all study subjects on CRFs.

17 ADMINISTRATIVE, ETHICAL, REGULATORY CONSIDERATIONS

The study will be conducted according to the Declaration of Helsinki, Protection of Human Volunteers (21 CFR 50), Institutional Review Boards (21 CFR 56), and Obligations of Clinical Investigators (21 CFR 312).

To maintain confidentiality, all research laboratory specimens, case report forms, and other study related records will be identified by a coded number only. Clinical laboratory specimens and requisition forms require participant identifiers (name, date of birth, medical record number, gender). All study records will be kept in a locked file cabinet in a locked room. Clinical information will not be released without written permission of the subject, except as necessary for monitoring by the FDA. The Investigator must also comply with all applicable privacy regulations (e.g., Health Insurance Portability and Accountability Act of 1996, EU Data Protection Directive 95/46/EC).

17.1 Protocol Amendments

Any amendment to the protocol will be written by PI. Protocol amendments cannot be implemented without prior written IRB/IEC approval except as necessary to eliminate immediate safety hazards to patients. Any amendment to the protocol requires CSRC approval. A protocol amendment intended to eliminate an apparent immediate hazard to patients may be implemented immediately, provided the IRBs are notified within five working days.

17.2 Institutional Review Boards and Independent Ethics Committees

The protocol and consent form will be reviewed and approved by the IRB/IEC of each participating center prior to study initiation. Serious adverse experiences regardless of causality will be reported to the IRB/IEC in accordance with the standard operating procedures and policies of the IRB/IEC, and the Investigator will keep the IRB/IEC informed as to the progress of the study. The Investigator will obtain assurance of IRB/IEC compliance with regulations.

Any documents that the IRB/IEC may need to fulfill its responsibilities (such as protocol, protocol amendments, Investigator's Brochure, consent forms, information concerning patient recruitment, payment or compensation procedures, or other pertinent information) will be submitted to the IRB/IEC. The IRB/IECs written unconditional approval of the study protocol and the informed consent form will be in the possession of the Investigator before the study is initiated. The IRB/IECs unconditional approval statement will be transmitted by the Investigator to any sponsor prior to the shipment of study supplies to the site. This approval must refer to the study by exact protocol title and number and should identify the documents reviewed and the date of review.

Protocol and/or informed consent modifications or changes may not be initiated without prior written IRB/IEC approval except when necessary to eliminate immediate hazards to the patients or when the change(s) involves only logistical or administrative aspects of the study. Such modifications will be submitted to the IRB/IEC and written verification that the modification was submitted and subsequently approved should be obtained.

The IRB/IEC must be informed of revisions to other documents originally submitted for review; serious and/or unexpected adverse experiences occurring during the study in accordance with the standard

operating procedures and policies of the IRB; new information that may affect adversely the safety of the patients of the conduct of the study; an annual update and/or request for re-approval; and when the study has been completed.

17.3 Informed Consent Form

Informed consent will be obtained in accordance with the Declaration of Helsinki, ICH GCP, US Code of Federal Regulations for Protection of Human Subjects (21 CFR 50.25, CFR 50.27, and CFR Part 56, Subpart A), the Health Insurance Portability and Accountability Act (HIPAA, if applicable), and local regulations.

The Investigator will prepare the informed consent form and HIPAA authorization and provide the documents to the Sponsor or designee for approval prior to submission to the IRB/IEC. The consent form generated by the Investigator must be acceptable to the Sponsor and be approved by the IRB/IEC. The written consent document will embody the elements of informed consent as described in the International Conference on Harmonisation and will also comply with local regulations. The Investigator will send an IRB/IEC-approved copy of the Informed Consent Form to the Sponsor (or designee) for the study file.

A properly executed, written, informed consent will be obtained from each subject prior to entering the subject into the trial. Information should be given in both oral and written form and subjects must be given ample opportunity to inquire about details of the study. If a subject is unable to sign the informed consent form (ICF) and the HIPAA authorization, a legal representative may sign for the subject. A copy of the signed consent form will be given to the subject and the original will be maintained with the subject's records. Participants who decline to receive a copy of the signed consent will have their preference documented.

17.4 Publications

The preparation and submittal for publication of manuscripts containing the study results shall be in accordance with a process determined by mutual written agreement among the study Sponsor and participating institutions. The publication or presentation of any study results shall comply with all applicable privacy laws, including, but not limited to, the Health Insurance Portability and Accountability Act of 1996.

17.5 Investigator Responsibilities

By signing the Agreement of Investigator form, the Investigator agrees to:

1. Conduct the study in accordance with the protocol and only make changes after notifying the Sponsor (or designee), except when to protect the safety, rights or welfare of subjects.

1. Personally conduct or supervise the study (or investigation).

2. Ensure that the requirements relating to obtaining informed consent and IRB review and approval meet federal guidelines, as stated in § 21 CFR, parts 50 and 56.

3. Report to the Sponsor or designee any AEs that occur in the course of the study, in accordance with §21 CFR 312.64.

4. Ensure that all associates, colleagues and employees assisting in the conduct of the study are informed about their obligations in meeting the above commitments.

5. Maintain adequate and accurate records in accordance with §21 CFR 312.62 and to make those records available for inspection with the Sponsor (or designee).

6. Ensure that an IRB that complies with the requirements of §21 CFR part 56 will be responsible for initial and continuing review and approval of the clinical study.

7. Promptly report to the IRB and the Sponsor (or designee) all changes in the research activity and all unanticipated problems involving risks to subjects or others (to include amendments and IND safety reports).

8. Seek IRB approval before any changes are made in the research study, except when necessary to eliminate hazards to the patients/subjects.

9. Comply with all other requirements regarding the obligations of clinical investigators and all other pertinent requirements listed in § 21 CFR part 312.

18 REFERENCES

1. Kalams, S.A., *et al.* Safety and comparative immunogenicity of an HIV-1 DNA vaccine in combination with plasmid interleukin 12 and impact of intramuscular electroporation for delivery. *J Infect Dis* **208**, 818-829 (2013).
2. Kalams, S.A., *et al.* Safety and immunogenicity of an HIV-1 gag DNA vaccine with or without IL-12 and/or IL-15 plasmid cytokine adjuvant in healthy, HIV-1 uninfected adults. *PLoS One* **7**, e29231 (2012).
3. Picker, L.J., Hansen, S.G. & Lifson, J.D. New paradigms for HIV/AIDS vaccine development. *Annual review of medicine* **63**, 95-111 (2012).
4. Haase, A.T. Perils at mucosal front lines for HIV and SIV and their hosts. *Nat Rev Immunol* **5**, 783-792 (2005).
5. Sneller, M.C., *et al.* A randomized controlled safety/efficacy trial of therapeutic vaccination in HIV-infected individuals who initiated antiretroviral therapy early in infection. *Sci Transl Med* **9**(2017).
6. Kaufmann, D.E., *et al.* Limited Durability of Viral Control following Treated Acute HIV Infection. *PLoS Med* **1**, e36 (2004).
7. Kulkarni, V., *et al.* Altered response hierarchy and increased T-cell breadth upon HIV-1 conserved element DNA vaccination in macaques. *PLoS One* **9**, e86254 (2014).
8. Hu, X., *et al.* DNA Prime-Boost Vaccine Regimen To Increase Breadth, Magnitude, and Cytotoxicity of the Cellular Immune Responses to Subdominant Gag Epitopes of Simian Immunodeficiency Virus and HIV. *J Immunol* **197**, 3999-4013 (2016).
9. Hu, X., *et al.* DNA Vaccine-Induced Long-Lasting Cytotoxic T Cells Targeting Conserved Elements of Human Immunodeficiency Virus Gag Are Boosted Upon DNA or Recombinant Modified Vaccinia Ankara Vaccination. *Hum Gene Ther* (2018).
10. Borducchi, E.N., *et al.* Ad26/MVA therapeutic vaccination with TLR7 stimulation in SIV-infected rhesus monkeys. *Nature* **540**, 284-287 (2016).
11. Lim, S.Y., *et al.* TLR7 agonists induce transient viremia and reduce the viral reservoir in SIV-infected rhesus macaques on antiretroviral therapy. *Sci Transl Med* **10**(2018).
12. Gautam, R., *et al.* A single injection of anti-HIV-1 antibodies protects against repeated SHIV challenges. *Nature* **533**, 105-109 (2016).
13. Rolland, M., Nickle, D.C. & Mullins, J.I. HIV-1 group M conserved elements vaccine. *PLoS Pathog* **3**, e157 (2007).
14. Niu, L., *et al.* Preclinical evaluation of HIV-1 therapeutic ex vivo dendritic cell vaccines expressing consensus Gag antigens and conserved Gag epitopes. *Vaccine* **29**, 2110-2119 (2011).
15. Mothe, B., *et al.* CTL responses of high functional avidity and broad variant cross-reactivity are associated with HIV control. *PLoS One* **7**, e29717 (2012).
16. Altfeld, M. & Allen, T.M. Hitting HIV where it hurts: an alternative approach to HIV vaccine design. *Trends Immunol* **27**, 504-510 (2006).

17. Letourneau, S., *et al.* Design and pre-clinical evaluation of a universal HIV-1 vaccine. *PLoS One* **2**, e984 (2007).
18. Rosario, M., *et al.* Long peptides induce polyfunctional T cells against conserved regions of HIV-1 with superior breadth to single-gene vaccines in macaques. *Eur J Immunol* **40**, 1973-1984 (2010).
19. Almeida, R.R., *et al.* Broad and cross-clade CD4+ T-cell responses elicited by a DNA vaccine encoding highly conserved and promiscuous HIV-1 M-group consensus peptides. *PloS One* **7**, e45267 (2012).
20. Stephenson, K.E., *et al.* Full-length HIV-1 immunogens induce greater magnitude and comparable breadth of T lymphocyte responses to conserved HIV-1 regions compared with conserved-region-only HIV-1 immunogens in rhesus monkeys. *J Virol* **86**, 11434-11440 (2012).
21. Kulkarni, V., *et al.* HIV-1 p24gag derived conserved element DNA vaccine increases the breadth of immune response in mice. *PLos One* **8**, e60245 (2013).
22. Kulkarni, V., *et al.* Altered Response Hierarchy and Increased T-cell Breadth upon HIV-1 Conserved Element DNA Vaccination in Macaques. *PLoS One* **9**, e86254. (2014).
23. Rosario, M., *et al.* Prime-boost regimens with adjuvanted synthetic long peptides elicit T cells and antibodies to conserved regions of HIV-1 in macaques. *Aids* **26**, 275-284 (2012).
24. Ribeiro, S.P., *et al.* A vaccine encoding conserved promiscuous HIV CD4 epitopes induces broad T cell responses in mice transgenic to multiple common HLA class II molecules. *PLoS One* **5**, e11072 (2010).
25. Mothe, B., *et al.* Definition of the viral targets of protective HIV-1-specific T cell responses. *J Transl Med* **9**, 208 (2011).
26. Martinez-Picado, J., *et al.* Fitness cost of escape mutations in p24 Gag in association with control of human immunodeficiency virus type 1. *J Virol* **80**, 3617-3623 (2006).
27. Peyerl, F.W., *et al.* Fitness costs limit viral escape from cytotoxic T lymphocytes at a structurally constrained epitope. *J Virol* **78**, 13901-13910 (2004).
28. Liu, Y., *et al.* Evolution of human immunodeficiency virus type 1 cytotoxic T-lymphocyte epitopes: fitness-balanced escape. *J Virol* **81**, 12179-12188 (2007).
29. Rolland, M., *et al.* HIV-1 conserved-element vaccines: relationship between sequence conservation and replicative capacity. *J Virol* **87**, 5461-5467 (2013).
30. Manocheewa, S., Swain, J.V., Lanxon-Cookson, E., Rolland, M. & Mullins, J.I. Fitness costs of mutations at the HIV-1 capsid hexamerization interface. *PLoS One* **8**, e66065 (2013).
31. Troyer, R.M., *et al.* Changes in human immunodeficiency virus type 1 fitness and genetic diversity during disease progression. *J Virol* **79**, 9006-9018 (2005).
32. Kulkarni, V., *et al.* HIV-1 Conserved Elements p24CE DNA Vaccine Induces Humoral Immune Responses with Broad Epitope Recognition in Macaques. *PLoS One* **9** e111085 (2014).
33. Mothe, B., *et al.* A Human Immune Data-Informed Vaccine Concept Elicits Strong and Broad T-cell Specificities Associated with HIV-1 Control in Mice and Macaques. *J Transl Med* **13**, 60 (2015).

34. Rolland, M., *et al.* Broad and Gag-biased HIV-1 epitope repertoires are associated with lower viral loads. *PLoS One* **3**, e1424 (2008).
35. Zuniga, R., *et al.* Relative dominance of Gag p24-specific cytotoxic T lymphocytes is associated with human immunodeficiency virus control. *J Virol* **80**, 3122-3125 (2006).
36. Masemola, A., *et al.* Hierarchical targeting of subtype C human immunodeficiency virus type 1 proteins by CD8+ T cells: correlation with viral load. *J Virol* **78**, 3233-3243 (2004).
37. Kiepiela, P., *et al.* CD8+ T-cell responses to different HIV proteins have discordant associations with viral load. *Nat Med* **13**, 46-53 (2007).
38. Honeyborne, I., *et al.* Control of human immunodeficiency virus type 1 is associated with HLA-B*13 and targeting of multiple gag-specific CD8+ T-cell epitopes. *J Virol* **81**, 3667-3672 (2007).
39. Schneidewind, A., *et al.* Structural and functional constraints limit options for cytotoxic T-lymphocyte escape in the immunodominant HLA-B27-restricted epitope in human immunodeficiency virus type 1 capsid. *J Virol* **82**, 5594-5605 (2008).
40. Ndhlovu, Z.M., *et al.* The breadth of expandable memory CD8+ T cells inversely correlates with residual viral loads in HIV elite controllers. *J Virol* **43**, 591-604 (2015).
41. Patel, V., *et al.* DNA and virus particle vaccination protects against acquisition and confers control of viremia upon heterologous SIV challenge. *Proc Natl Acad Sci U S A* **110**, 2975-2980 (2013).
42. Martins, M.A., *et al.* Vaccine-induced immune responses against both Gag and Env improve control of simian immunodeficiency virus replication in rectally challenged rhesus macaques. *PLoS Pathog* **13**, e1006529 (2017).
43. Schell, J.B., *et al.* Antigenic requirement for Gag in a vaccine that protects against high-dose mucosal challenge with simian immunodeficiency virus. *Virology* **476**, 405-412 (2015).
44. Schell, J.B., *et al.* Significant protection against high-dose simian immunodeficiency virus challenge conferred by a new prime-boost vaccine regimen. *J Virol* **85**, 5764-5772 (2011).
45. Hansen, S.G., *et al.* Immune clearance of highly pathogenic SIV infection. *Nature* **502**, 100-104 (2013).
46. Hansen, S.G., *et al.* Broadly targeted CD8(+) T cell responses restricted by major histocompatibility complex E. *Science* **351**, 714-720 (2016).
47. Herbeck, J.T., *et al.* Human immunodeficiency virus type 1 env evolves toward ancestral states upon transmission to a new host. *J Virol* **80**, 1637-1644 (2006).
48. Duda, A., *et al.* HLA-associated clinical progression correlates with epitope reversion rates in early human immunodeficiency virus infection. *J Virol* **83**, 1228-1239 (2009).
49. Li, B., *et al.* Rapid reversion of sequence polymorphisms dominates early human immunodeficiency virus type 1 evolution. *J Virol* **81**, 193-201 (2007).
50. Nickle, D.C., *et al.* Consensus and ancestral state HIV vaccines. *Science* **299**, 1515-1518; author reply 1515-1518 (2003).

51. Buseyne, F., *et al.* Frequencies of ex vivo-activated human immunodeficiency virus type 1-specific gamma-interferon-producing CD8+ T cells in infected children correlate positively with plasma viral load. *J Virol* **76**, 12414-12422 (2002).
52. Ramduth, D., *et al.* Differential immunogenicity of HIV-1 clade C proteins in eliciting CD8+ and CD4+ cell responses. *J Infect Dis* **192**, 1588-1596 (2005).
53. Riviere, Y., *et al.* Gag-specific cytotoxic responses to HIV type 1 are associated with a decreased risk of progression to AIDS-related complex or AIDS. *AIDS research and human retroviruses* **11**, 903-907 (1995).
54. Edwards, B.H., *et al.* Magnitude of functional CD8+ T-cell responses to the gag protein of human immunodeficiency virus type 1 correlates inversely with viral load in plasma. *J Virol* **76**, 2298-2305 (2002).
55. Novitsky, V., *et al.* Association between virus-specific T-cell responses and plasma viral load in human immunodeficiency virus type 1 subtype C infection. *J Virol* **77**, 882-890 (2003).
56. Ogg, G.S., *et al.* Quantitation of HIV-1-specific cytotoxic T lymphocytes and plasma load of viral RNA. *Science* **279**, 2103-2106. (1998).
57. Sacha, J.B., *et al.* Gag-specific CD8+ T lymphocytes recognize infected cells before AIDS-virus integration and viral protein expression. *J Immunol* **178**, 2746-2754 (2007).
58. Geldmacher, C., *et al.* CD8 T-cell recognition of multiple epitopes within specific Gag regions is associated with maintenance of a low steady-state viremia in human immunodeficiency virus type 1-seropositive patients. *J Virol* **81**, 2440-2448 (2007).
59. Carlson, J.M., *et al.* Correlates of protective cellular immunity revealed by analysis of population-level immune escape pathways in HIV-1. *J Virol* **86**, 13202-13216 (2012).
60. Carlson, J.M., Le, A.Q., Shahid, A. & Brumme, Z.L. HIV-1 adaptation to HLA: a window into virus-host immune interactions. *Trends Microbiol* **23**, 212-224 (2015).
61. Keane, N.M., *et al.* High-avidity, high-IFNgamma-producing CD8 T-cell responses following immune selection during HIV-1 infection. *Immunol Cell Biol* **90**, 224-234 (2012).
62. Troyer, R.M., *et al.* Variable fitness impact of HIV-1 escape mutations to cytotoxic T lymphocyte (CTL) response. *PLoS Pathog* **5**, e1000365 (2009).
63. Liu, Y., *et al.* Impact of mutations in highly conserved amino acids of the HIV-1 Gag-p24 and Env-gp120 proteins on viral replication in different genetic backgrounds. *PLoS One* **9**, e94240 (2014).
64. Rihn, S.J., *et al.* Extreme genetic fragility of the HIV-1 capsid. *PLoS Pathog* **9**, e1003461 (2013).
65. Almeida, C.A., *et al.* Translation of HLA-HIV associations to the cellular level: HIV adapts to inflate CD8 T cell responses against Nef and HLA-adapted variant epitopes. *J Immunol* **187**, 2502-2513 (2011).
66. Dinges, W.L., *et al.* Virus-specific CD8+ T-cell responses better define HIV disease progression than HLA genotype. *J Virol* **84**, 4461-4468 (2010).

67. Boutwell, C.L., Rowley, C.F. & Essex, M. Reduced viral replication capacity of human immunodeficiency virus type 1 subtype C caused by cytotoxic-T-lymphocyte escape mutations in HLA-B57 epitopes of capsid protein. *J Virol* **83**, 2460-2468 (2009).

68. Altfeld, M*., et al.* Influence of HLA-B57 on clinical presentation and viral control during acute HIV-1 infection. *Aids* **17**, 2581-2591 (2003).

69. Frahm, N*., et al.* HLA-B63 presents HLA-B57/B58-restricted cytotoxic T-lymphocyte epitopes and is associated with low human immunodeficiency virus load. *J Virol* **79**, 10218-10225 (2005).

70. Streeck, H*., et al.* Recognition of a defined region within p24 gag by CD8+ T cells during primary human immunodeficiency virus type 1 infection in individuals expressing protective HLA class I alleles. *J Virol* **81**, 7725-7731 (2007).

71. Miura, T*., et al.* HLA-B57/B*5801 human immunodeficiency virus type 1 elite controllers select for rare gag variants associated with reduced viral replication capacity and strong cytotoxic T-lymphocyte [corrected] recognition. *J Virol* **83**, 2743-2755 (2009).

72. Dyer, W.B*., et al.* Mechanisms of HIV non-progression; robust and sustained CD4+ T-cell proliferative responses to p24 antigen correlate with control of viraemia and lack of disease progression after long-term transfusion-acquired HIV-1 infection. *Retrovirology* **5**, 112 (2008).

73. Schneidewind, A*., et al.* Escape from the dominant HLA-B27-restricted cytotoxic T-lymphocyte response in Gag is associated with a dramatic reduction in human immunodeficiency virus type 1 replication. *J Virol* **81**, 12382-12393 (2007).

74. den Uyl, D., van der Horst-Bruinsma, I. & van Agtmael, M. Progression of HIV to AIDS: a protective role for HLA-B27? *AIDS Rev* **6**, 89-96 (2004).

75. Miura, T*., et al.* HLA-associated viral mutations are common in human immunodeficiency virus type 1 elite controllers. *J Virol* **83**, 3407-3412 (2009).

76. Lassen, K.G*., et al.* Elite suppressor-derived HIV-1 envelope glycoproteins exhibit reduced entry efficiency and kinetics. *PLoS Pathog* **5**, e1000377 (2009).

77. Liu, Y*., et al.* Conserved HIV-1 epitopes continuously elicit subdominant cytotoxic T-lymphocyte responses. *J Infect Dis* **200**, 1825-1833 (2009).

78. Friedrich, T.C*., et al.* Subdominant CD8+ T-cell responses are involved in durable control of AIDS virus replication. *J Virol* **81**, 3465-3476 (2007).

79. Frahm, N*., et al.* Control of human immunodeficiency virus replication by cytotoxic T lymphocytes targeting subdominant epitopes. *Nat Immunol* **7**, 173-178 (2006).

80. Sardesai, N.Y. & Weiner, D.B. Electroporation delivery of DNA vaccines: prospects for success. *Curr Opin Immunol* **23**, 421-429 (2011).

81. Flingai, S*., et al.* Synthetic DNA vaccines: improved vaccine potency by electroporation and co-delivered genetic adjuvants. *Front Immunol* **4**, 354 (2013).

82. Hutnick, N.A., Myles, D.J., Bian, C.B., Muthumani, K. & Weiner, D.B. Selected approaches for increasing HIV DNA vaccine immunogenicity in vivo. *Curr Opin Virol* **1**, 233-240 (2012).

83. Felber, B.K., Valentin, A., Rosati, M., Bergamaschi, C. & Pavlakis, G.N. HIV DNA Vaccine: Stepwise improvements make a difference. *Vaccines* **2**, 354-379 (2014).

84. Bodles-Brakhop, A.M., Heller, R. & Draghia-Akli, R. Electroporation for the Delivery of DNA-based Vaccines and Immunotherapeutics: Current Clinical Developments. *Mol Ther* **17**, 585-592 (2009).
85. Hu, X., *et al.* DNA Prime-boost Vaccine Regimen to Increase Breadth, Magnitude, and Cytotoxicity of the Cellular Immune Responses to Subdominant Gag Epitopes of SIV and HIV *J Immunol* **197**, 3999-4013 (2016).
86. Wyatt, L.S., *et al.* Multiprotein HIV type 1 clade B DNA and MVA vaccines: construction, expression, and immunogenicity in rodents of the MVA component. *AIDS Res Hum Retroviruses* **20**, 645-653 (2004).
87. Valentin, A., *et al.* Dose-dependent Inhibition of Gag Cellular Immunity by Env in SIV/HIV DNA Vaccinated Macaques. *Hum Vaccin Immunother.* **11**, 2005-2011 (2015).
88. Valentin, A., *et al.* Repeated DNA therapeutic vaccination of chronically SIV-infected macaques provides additional virological benefit. *Vaccine* **28**, 1962-1974 (2010).
89. von Gegerfelt, A.S., *et al.* Long-lasting decrease in viremia in macaques chronically infected with simian immunodeficiency virus SIVmac251 after therapeutic DNA immunization. *J Virol* **81**, 1972-1979 (2007).
90. Valentin, A., *et al.* Comparative Analysis of SIV-specific Cellular Immune Responses Induced by Different Vaccine Platforms in Rhesus Macaques. *Clin Immunol* **55**, 91-107 (2014).
91. Patel, V., *et al.* Long-lasting humoral and cellular immune responses and mucosal dissemination after intramuscular DNA immunization. *Vaccine* **28**, 4827-4836 (2010).
92. Munson, P., *et al.* Therapeutic conserved elements (CE) DNA vaccine induces strong T-cell responses against highly conserved viral sequences during simian-human immunodeficiency virus infection. *Hum Vaccin Immunother*, 1-12 (2018).
93. Xu, R., *et al.* Comparative ability of various plasmid-based cytokines and chemokines to adjuvant the activity of HIV plasmid DNA vaccines. *Vaccine* **26**, 4819-4829 (2008).
94. Chong, S.Y., *et al.* Comparative ability of plasmid IL-12 and IL-15 to enhance cellular and humoral immune responses elicited by a SIVgag plasmid DNA vaccine and alter disease progression following SHIV(89.6P) challenge in rhesus macaques. *Vaccine* **25**, 4967-4982 (2007).
95. Egan, M.A., *et al.* Priming with Plasmid DNAs Expressing Interleukin-12 and Simian Immunodeficiency Virus Gag Enhances the Immunogenicity and Efficacy of an Experimental AIDS Vaccine Based on Recombinant Vesicular Stomatitis Virus. *AIDS Res Hum Retroviruses* **21**, 629-643 (2005).
96. Egan, M.A., *et al.* Rational design of a plasmid DNA vaccine capable of eliciting cell-mediated immune responses to multiple HIV antigens in mice. *Vaccine* **24**, 4510-4523 (2006).
97. Jalah, R., *et al.* IL-12 DNA as molecular vaccine adjuvant increases the cytotoxic T cell responses and breadth of humoral immune responses in SIV DNA vaccinated macaques. *Hum Vaccin Immunother* **8**, 1620-1629 (2012).

98. Hirao, L.A., *et al.* Combined effects of IL-12 and electroporation enhances the potency of DNA vaccination in macaques. *Vaccine* **26**, 3112-3120 (2008).

99. Jacobson, J.M., *et al.* The Safety and Immunogenicity of an Interleukin-12-Enhanced Multiantigen DNA Vaccine Delivered by Electroporation for the Treatment of HIV-1 Infection. *J Acquir Immune Defic Syndr* **71**, 163-171 (2016).

100. Mpendo, J., *et al.* A Phase I Double Blind, Placebo-Controlled, Randomized Study of the Safety and Immunogenicity of Electroporated HIV DNA with or without Interleukin 12 in Prime-Boost Combinations with an Ad35 HIV Vaccine in Healthy HIV-Seronegative African Adults. *PLoS One* **10**, e0134287 (2015).

101. Li, S.S., *et al.* DNA Priming Increases Frequency of T-Cell Responses to a Vesicular Stomatitis Virus HIV Vaccine with Specific Enhancement of CD8(+) T-Cell Responses by Interleukin-12 Plasmid DNA. *Clin Vaccine Immunol* **24**(2017).

102. Hay, C.H., *et al.* IL-12 increases CD8+ but not CD4+ T-cell responses in a DNA/VSV-HIV vaccine in phase I clinical trial

. *in preparation*.

103. Heller, R. & Heller, L.C. Gene electrotransfer clinical trials. *Adv Genet* **89**, 235-262 (2015).

104. Vasan, S. Electroporation-mediated administration of candidate DNA vaccines against HIV-1. *Methods in molecular biology* **1121**, 291-307 (2014).

105. Broderick, K.E., Khan, A.S. & Sardesai, N.Y. DNA vaccination in skin enhanced by electroporation. *Methods in molecular biology* **1143**, 123-130 (2014).

106. Khan, A.S., Broderick, K.E. & Sardesai, N.Y. Clinical development of intramuscular electroporation: providing a "boost" for DNA vaccines. *Methods in molecular biology* **1121**, 279-289 (2014).

107. Smith, J.M., *et al.* Multiprotein HIV-1 Clade B DNA/MVA Vaccine: Construction, Safety and Immunogenicity. *AIDS Research and Human Retroviruses* **20**, 654-665 (2004).

108. Wyatt, L.S., *et al.* Correlation of immunogenicities and in vitro expression levels of recombinant modified vaccinia virus Ankara HIV vaccines. *Vaccine* **26**, 486-493 (2008).

109. Smith, J.M., *et al.* DNA/MVA vaccine for HIV type 1: effects of codon-optimization and the expression of aggregates or virus-like particles on the immunogenicity of the DNA prime. *AIDS Res Hum Retroviruses* **20**, 1335-1347 (2004).

110. Kapp, K., Kleuss, C., Schroff, M. & Wittig, B. Genuine Immunomodulation With dSLIM. *Molecular therapy. Nucleic acids* **3**, e170 (2014).

111. Schmoll, H.J., *et al.* Maintenance treatment with the immunomodulator MGN1703, a Toll-like receptor 9 S(TLR9) agonist, in patients with metastatic colorectal carcinoma and disease control after chemotherapy: a randomised, double-blind, placebo-controlled trial. *Journal of cancer research and clinical oncology* **140**, 1615-1624 (2014).

112. Krarup, A.R., *et al.* The TLR9 agonist MGN1703 triggers a potent type I interferon response in the sigmoid colon. *Mucosal Immunol* (2017).

113. Mouquet, H., *et al.* Complex-type N-glycan recognition by potent broadly neutralizing HIV antibodies. *Proc Natl Acad Sci U S A* **109**, E3268-3277 (2012).

114. Mouquet, H., *et al.* Memory B cell antibodies to HIV-1 gp140 cloned from individuals infected with clade A and B viruses. *PloS One* **6**, e24078 (2011).

115. West, A.P., Jr., *et al.* Computational analysis of anti-HIV-1 antibody neutralization panel data to identify potential functional epitope residues. *Proc Natl Acad Sci U S A* **110**, 10598-10603 (2013).

116. Klein, F., *et al.* HIV therapy by a combination of broadly neutralizing antibodies in humanized mice. *Nature* **492**, 118-122 (2012).

117. Horwitz, J.A., *et al.* HIV-1 suppression and durable control by combining single broadly neutralizing antibodies and antiretroviral drugs in humanized mice. *Proc. Natl. Acad. Sci. USA* **110**, 16538-16543 (2013).

118. Shingai, M., *et al.* Antibody-mediated immunotherapy of macaques chronically infected with SHIV suppresses viraemia. *Nature* (2013).

119. Shingai, M., *et al.* Passive transfer of modest titers of potent and broadly neutralizing anti-HIV monoclonal anitbodies block SHIV infection in macaques. *Journal of Experimental Medicine* **In Press**(2014).

120. Gautam, R., *et al.* A single injection of anti-HIV-1 antibodies protects against repeated SHIV challenges. *Nature* **533**, 105-109 (2016).

121. Caskey, M., *et al.* Antibody 10-1074 suppresses viremia in HIV-1-infected individuals. *Nat Med* (2017).

122. Lu, C.L., *et al.* Enhanced clearance of HIV-1-infected cells by broadly neutralizing antibodies against HIV-1 in vivo. *Science* **352**, 1001-1004 (2016).

123. Li, Y., *et al.* Broad HIV-1 neutralization mediated by CD4-binding site antibodies. *Nat Med* **13**, 1032-1034 (2007).

124. Wu, X., *et al.* Rational design of envelope identifies broadly neutralizing human monoclonal antibodies to HIV-1. *Science* **329**, 856-861 (2010).

125. Zalevsky, J., *et al.* Enhanced antibody half-life improves in vivo activity. *Nat Biotechnol* **28**, 157-159 (2010).

126. Ko, S.Y., *et al.* Enhanced neonatal Fc receptor function improves protection against primate SHIV infection. *Nature* **514**, 642-645 (2014).

127. Rudicell, R.S., *et al.* Enhanced potency of a broadly neutralizing HIV-1 antibody in vitro improves protection against lentiviral infection in vivo. *J Virol* **88**, 12669-12682 (2014).

128. Bolton, D.L., *et al.* Human Immunodeficiency Virus Type 1 Monoclonal Antibodies Suppress Acute Simian-Human Immunodeficiency Virus Viremia and Limit Seeding of Cell-Associated Viral Reservoirs. *J Virol* **90**, 1321-1332 (2016).

129. Patel, S.V. & Khan, D.A. Adverse Reactions to Biologic Therapy. *Immunol Allergy Clin North Am* **37**, 397-412 (2017).

130. Henrich, T.J., *et al.* HIV-1 persistence following extremely early initiation of antiretroviral therapy (ART) during acute HIV-1 infection: An observational study. *PLoS Med* **14**, e1002417 (2017).

131. Nishimura, Y., *et al.* Early antibody therapy can induce long-lasting immunity to SHIV. *Nature* **543**, 559-563 (2017).

132. Namazi, G., *et al.* The Control of HIV after Antiretroviral Medication Pause (CHAMP) study: post-treatment controllers identified from 14 clinical studies. *J Infect Dis* (2018).

133. Hartikka, J., *et al.* An improved plasmid DNA expression vector for direct injection into skeletal muscle. *Hum Gene Ther* **7**, 1205-1217 (1996).

134. Graham, B.S., *et al.* Phase 1 safety and immunogenicity evaluation of a multiclade HIV-1 DNA candidate vaccine. *J Infect Dis* **194**, 1650-1660 (2006).

135. MacGregor, R.R., Boyer, J.D., Ciccarelli, R.B., Ginsberg, R.S. & Weiner, D.B. Safety and immune responses to a DNA-based human immunodeficiency virus (HIV) type I env/rev vaccine in HIV-infected recipients: follow-up data. *J Infect Dis* **181**, 406. (2000).

136. MacGregor, R.R., *et al.* First human trial of a DNA-based vaccine for treatment of human immunodeficiency virus type 1 infection: safety and host response. *J Infect Dis* **178**, 92-100. (1998).

137. MacGregor, R.R., *et al.* Plasmid vaccination of stable HIV-positive subjects on antiviral treatment results in enhanced CD8 T-cell immunity and increased control of viral "blips". *Vaccine* **23**, 2066-2073 (2005).

138. MacGregor, R.R., *et al.* T-cell responses induced in normal volunteers immunized with a DNA-based vaccine containing HIV-1 env and rev. *Aids* **16**, 2137-2143 (2002).

139. Hejdeman, B., *et al.* DNA immunization with HIV early genes in HIV type 1-infected patients on highly active antiretroviral therapy. *AIDS Res Hum Retroviruses* **20**, 860-870 (2004).

140. Vasan, S., *et al.* Phase 1 safety and immunogenicity evaluation of ADVAX, a multigenic, DNA-based clade C/B' HIV-1 candidate vaccine. *PLoS One* **5**, e8617 (2010).

141. Guimaraes-Walker, A., *et al.* Lessons from IAVI-006, a phase I clinical trial to evaluate the safety and immunogenicity of the pTHr.HIVA DNA and MVA.HIVA vaccines in a prime-boost strategy to induce HIV-1 specific T-cell responses in healthy volunteers. *Vaccine* **26**, 6671-6677 (2008).

142. Goonetilleke, N., *et al.* Induction of multifunctional human immunodeficiency virus type 1 (HIV-1)-specific T cells capable of proliferation in healthy subjects by using a prime-boost regimen of DNA- and modified vaccinia virus Ankara-vectored vaccines expressing HIV-1 Gag coupled to CD8+ T-cell epitopes. *J Virol* **80**, 4717-4728 (2006).

143. Calarota, S., *et al.* Cellular cytotoxic response induced by DNA vaccination in HIV-1-infected patients. *Lancet* **351**, 1320-1325. (1998).

144. Sheets, R.L., *et al.* Toxicological safety evaluation of DNA plasmid vaccines against HIV-1, Ebola, Severe Acute Respiratory Syndrome, or West Nile virus is similar despite differing

plasmid backbones or gene-inserts. *Toxicological sciences : an official journal of the Society of Toxicology* **91**, 620-630 (2006).

145. Sheets, R.L., *et al.* Biodistribution of DNA plasmid vaccines against HIV-1, Ebola, Severe Acute Respiratory Syndrome, or West Nile virus is similar, without integration, despite differing plasmid backbones or gene inserts. *Toxicological sciences : an official journal of the Society of Toxicology* **91**, 610-619 (2006).
146. Pachuk, C.J., *et al.* Characterization of a new class of DNA delivery complexes formed by the local anesthetic bupivacaine. *Biochim Biophys Acta* **1468**, 20-30 (2000).
147. Richman, D.D., Wrin, T., Little, S.J. & Petropoulos, C.J. Rapid evolution of the neutralizing antibody response to HIV type 1 infection. *Proc Natl Acad Sci U S A* **100**, 4144-4149 (2003).
148. Mohammadi, P., *et al.* Dynamics of HIV latency and reactivation in a primary CD4+ T cell model. *PLoS Pathog* **10**, e1004156 (2014).
149. Chung, A.W., *et al.* Dissecting Polyclonal Vaccine-Induced Humoral Immunity against HIV Using Systems Serology. *Cell* **163**, 988-998 (2015).
150. Ho, Y.C., *et al.* Replication-Competent Noninduced Proviruses in the Latent Reservoir Increase Barrier to HIV-1 Cure. *Cell* **155**, 540-551 (2013).
151. Siliciano, R.F. Understanding persistence of the latent reservoir. in *HIV Persistence During Therapy: Reservoirs & Eradication Strategies Workshop; 9th Edition* (Miami, FL, 2017).
152. Laird, G.M. Intact Proviral DNA Assay: a new method for quantifying the HIV-1 latent reservoir. in *AIDS Clinical Trials Group Annual Meeting: Cure Transformative Science Group* (Boston, MA, 2018).
153. Bruner, K.M.M., A. J.; Ho, Y.C.; Laird, G.M.; Wang, Z.; Kwon, K.J.; Beg, S.A.; Timmons, A.; Laskey, S.B.; Clements, J.E.; Siliciano, J.D.; Siliciano, R.F. Novel paradigm for measuring HIV-1 reservoir allows quantitation of intact proviruses. in *Conference on Retroviruses and Opportunistic Infections* (Boston, MA, 2018).
154. Kumar, A.M., Borodowsky, I., Fernandez, B., Gonzalez, L. & Kumar, M. Human immunodeficiency virus type 1 RNA Levels in different regions of human brain: quantification using real-time reverse transcriptase-polymerase chain reaction. *Journal of neurovirology* **13**, 210-224 (2007).
155. Vandergeeten, C., *et al.* Cross-clade ultrasensitive PCR-based assays to measure HIV persistence in large-cohort studies. *J Virol* **88**, 12385-12396 (2014).
156. Deeks, S.G., *et al.* Virologic and immunologic consequences of discontinuing combination antiretroviral-drug therapy in HIV-infected patients with detectable viremia. *N Engl J Med* **344**, 472-480. (2001).
157. Deeks, S.G., *et al.* Interruption of Enfuvirtide in HIV-1-Infected Adults with Incomplete Viral Suppression on an Enfuvirtide-Based Regimen. *J Infect Dis* **195**, 387-391 (2007).
158. Beatty, G., *et al.* A randomized pilot study comparing combination therapy plus enfuvirtide versus a treatment interruption followed by combination therapy plus enfuvirtide. *Antivir Ther* **11**, 315-319 (2006).

159. Deeks, S.G., *et al.* Interruption of Treatment with Individual Therapeutic Drug Classes in Adults with Multidrug-Resistant HIV-1 Infection. *J Infect Dis* **192**, 1537-1544 (2005).
160. Napolitano, L.A., *et al.* Growth hormone enhances thymic function in HIV-1-infected adults. *J Clin Invest* (2008).
161. Probasco, J.C., *et al.* Failure of atorvastatin to modulate CSF HIV-1 infection: results of a pilot study. *Neurology* **71**, 521-524 (2008).
162. Hunt, P.W., *et al.* Valganciclovir Reduces T Cell Activation in HIV-infected Individuals With Incomplete CD4+ T Cell Recovery on Antiretroviral Therapy. *J Infect Dis* **203**, 1474-1483 (2011).
163. Spivak, A.M., *et al.* A pilot study assessing the safety and latency-reversing activity of disulfiram in HIV-1-infected adults on antiretroviral therapy. *Clin Infect Dis* **58**, 883-890 (2014).
164. Elliott, J.H., *et al.* Short-term administration of disulfiram for reversal of latent HIV infection: a phase 2 dose-escalation study. *Lancet HIV* **2**, e520-529 (2015).
165. Somsouk, M., *et al.* The immunologic effects of mesalamine in treated HIV-infected individuals with incomplete CD4+ T cell recovery: a randomized crossover trial. *PLoS One* **9**, e116306 (2014).
166. Cockerham, L.R., *et al.* A Randomized Controlled Trial of Lisinopril to Decrease Lymphoid Fibrosis in Antiretroviral-Treated, HIV-infected Individuals. *Pathog Immun* **2**, 310-334 (2017).
167. Deeks, S.G., *et al.* A Phase II Randomized Study of HIV-Specific T-Cell Gene Therapy in Subjects with Undetectable Plasma Viremia on Combination Antiretroviral Therapy. *Mol Ther* **5**, 788-797 (2002).
168. Hatano, H., *et al.* A Randomized, Controlled Trial Assessing the Effects of Raltegravir Intensification on Endothelial Function in Treated HIV Infection. *J Acquir Immune Defic Syndr* (2012).
169. Hatano, H., *et al.* A randomized, controlled trial of raltegravir intensification in antiretroviral-treated, HIV-infected patients with a suboptimal CD4+ T cell response. *J Infect Dis* **203**, 960-968 (2011).
170. Hatano, H., *et al.* Increase in 2-Long Terminal Repeat Circles and Decrease in D-dimer After Raltegravir Intensification in Patients With Treated HIV Infection: A Randomized, Placebo-Controlled Trial. *J Infect Dis* **208**, 1436-1442 (2013).
171. Hunt, P.W., *et al.* The immunologic effects of maraviroc intensification in treated HIV-infected individuals with incomplete CD4+ T-cell recovery: a randomized trial. *Blood* **121**, 4635-4646 (2013).
172. Hatano, H., *et al.* Prospective Antiretroviral Treatment of Asymptomatic, HIV-1 Infected Controllers. *PLoS Pathog* **9**, e1003691 (2013).
173. Achenbach, C.J., *et al.* Effect of therapeutic intensification followed by HIV DNA prime and rAd5 boost vaccination on HIV-specific immunity and HIV reservoir (EraMune 02): a multicentre randomised clinical trial. *Lancet HIV* **2**, e82-91 (2015).

19 CONSENT TO PARTICIPATE IN A RESEARCH STUDY

See attached consent form version 2.2.

20 APPENDIX A: POTENTIAL IMMUNE-MEDIATED MEDICAL CONDITIONS

Gastrointestinal disorders

Celiac disease

Crohn’s disease

Ulcerative colitis

Ulcerative proctitis

Liver disorders

Autoimmune cholangitis

Autoimmune hepatitis

Primary biliary cirrhosis

Primary sclerosing cholangitis

Metabolic diseases

Addison’s disease

Autoimmune thyroiditis (including Hashimoto thyroiditis)

Diabetes mellitus type I

Grave's or Basedow’s disease

Musculoskeletal disorders

Antisynthetase syndrome

Dermatomyositis

Juvenile chronic arthritis (including Still’s disease)

Mixed connective tissue disorder

Polymyalgia rheumatic

Polymyositis

Psoriatic arthropathy

Relapsing polychondritis

Rheumatoid arthritis

Scleroderma, including diffuse systemic form and CREST syndrome

Spondyloarthritis, including ankylosing spondylitis, reactive arthritis (Reiter's Syndrome) and undifferentiated spondyloarthritis

Systemic lupus erythematosus

Systemic sclerosis

Neuroinflammatory disorders

Acute disseminated encephalomyelitis, including site specific variants (e.g., non-infectious encephalitis, encephalomyelitis, myelitis, radiculomyelitis)

Cranial nerve disorders, including paralyses/paresis (e.g., Bell's palsy)

Guillain-Barré syndrome, including Miller Fisher syndrome and other variants

Immune-mediated peripheral neuropathies and plexopathies, including chronic inflammatory demyelinating polyneuropathy, multifocal motor neuropathy and polyneuropathies associated with monoclonal gammopathy

Multiple sclerosis

Narcolepsy

Optic neuritis

Transverse myelitis

Myasthenia gravis, including Eaton-Lambert syndrome

Skin disorders

Alopecia areata

Autoimmune bullous skin diseases, including pemphigus, pemphigoid and dermatitis herpetiformis

Cutaneous lupus erythematosus

Erythema nodosum

Morphoea

Lichen planus

Severe psoriasis or psoriasis requiring systemic immunotherapy within the last year. Individuals with localized disease treated with topical therapy alone will not be excluded.

Rosacea

Sweet’s syndrome

Vitiligo

Vasculitides

Large vessels vasculitis including: giant cell arteritis such as Takayasu's arteritis and temporal arteritis

- Medium sized and/or small vessels vasculitis including: polyarteritis nodosaKawasaki's d iseasemicroscopic polyangiitisWegener's granulomatosisChurg–Strauss syndrome (allergic granulomatous angiitis)
- Buerger’s disease
- thromboangiitis obliteransnecrotizing vasculitis
- antineutrophil cytoplasmic antibody (ANCA) positive vasculitis (type unspecified)

Henoch- Schonlein purpuraBehcet's syndromeleukocytoclastic vasculitis

Others

Antiphospholipid syndrome

Autoimmune hemolytic anemia

Autoimmune glomerulonephritis (including IgA nephropathy, glomerulonephritis rapidly progressive, membranous glomerulonephritis, membranoproliferative glomerulonephritis, and mesangioproliferative glomerulonephritis)

Autoimmune myocarditis/cardiomyopathy

Autoimmune thrombocytopenia

Goodpasture syndrome

Idiopathic pulmonary fibrosis

Pernicious anemia

Raynaud’s phenomenon secondary to a serious immune-mediated disease or requiring immunotherapy within the last year

Sarcoidosis

Sjögren’s syndrome

Stevens-Johnson syndrome

Uveitis

21 APPENDIX B: RISK MITIGATION DURING ATI

ATIs are associated with substantial risk to both study participants and their sexual partner(s). Two documented HIV transmissions occurring in the context of ATIs have recently been reported. In collaboration with our Community Advisory Board, we have developed a practical approach to risk mitigation during this study, which has been peer-reviewed and published (see Journal of Virus Eradication 2020).

Treatment Interruption Risk Mitigation Procedures

The study PI, Co-I, or designee will document that they have performed the procedures outlined in the **Pre-ATI Risk Mitigation Counseling Checklist** prior to the B2D0 visit and prior to the Week 34 visit. Following Week 34, they will document that they have performed the procedures outlined in the **Ongoing Risk Mitigation Counseling Checklist** at least every 4-6 weeks during the ATI.

Participant Handouts

Prior to the B2D0 visit, Week 34 visit, and every 4-6 weeks during the ATI, participants will be offered the following educational materials:

- ATI Disclosure Sheet
- Pre-Exposure Prophylaxis for Partners handout
- Pre-Exposure Prophylaxis for Cisgender Women handout (only if participant is sexually active with cisgender women)

22 APPENDIX C: COVID-19

The SARS-CoV-2 pandemic has had a major impact on research operations worldwide. As shelter-in-place guidelines were implemented throughout early 2020, many centers suspended research efforts to protect study participants and research staff while the scientific and medical communities raced to understand this novel infection. Recognizing and mitigating the risk of SARS-CoV-2 infection is a critical step in ensuring that research studies can be conducted safely and ethically in the context of the ongoing pandemic.

In collaboration with our Community Advisory Board (CAB), we recently published a consensus document outlining the risks and risk mitigation strategy for this clinical trial (see Peluso et al CID 2020). This section will outline the approach we will take. The items outlined in this section supersede the content of the Protocol document and reflect the most up-to-date guidance on the issues discussed.

Risk-benefit Assessment

The study team sought the advice of the CAB whose assessment that the risk-benefit assessment as of August 2020 was in favor of re-opening the study. The study team will follow the advice of the Safety Monitoring Committee (SMC), in conjunction with the CAB, with regard to whether the risk-benefit assessment changes over time. In such a case, a change to the risk-benefit assessment may necessitate pausing or delaying the trial. Such a decision will be made in conjunction with the SMC and CAB.

Changes to the Main Study Protocol

The maximum age of at the time of enrollment will be decreased from 67 to 65 years of age.

Participants will not be asked to commit to the analytic treatment interruption (ATI) at the time of enrollment. Prior to the beginning of the ATI (approximately Weeks 30-Week 34), the Principal Investigator or Co-Principal Investigator will discuss with each participant the current state of the SARS-CoV-2 pandemic. They will come to a shared decision regarding participation in the ATI (this decision may also involve input from the participant's primary care or HIV provider), and the participant will choose to opt into or opt out of the ATI. The participant will be asked to sign the consent form again should they choose to participate in the ATI at this time.

ATI-specific Exclusion Criteria

While the overall study inclusion criteria remain the same (with the exception of age as above), we will implement additional exclusions related to the ATI. These criteria will be assessed within 6 weeks prior to the ATI. Participants will be ineligible to participate in the ATI in the case of any of the below:

1. Unable or unwilling to practice up-to-date CDC recommendations including physical distancing and masking in situations where physical distancing is not possible.

2. Active tobacco vaping or smoking defined as smoking >= 0.5 packs per day (>=10 cigarettes per day), if unwilling to quit.
    a. Active smokers will be referred to their primary care physician for assistance in quitting.
3. Poorly controlled asthma or chronic obstructive pulmonary disorder, defined as two or more hospitalizations for such conditions within the 1 year preceding the ATI.
4. Uncontrolled hypertension, defined as systolic blood pressure >160 or diastolic blood pressure >100 on repeat measurements at two consecutive visits within 4 weeks of the ATI.
5. In an individual with a known diagnosis of diabetes, poor glucose control despite medical therapy, defined as hemoglobin A1c greater than 9.0% on the most recent test within 6 months of the ATI. The hemoglobin A1c may be checked at any study visit in the 6 months preceding the ATI.
6. Severe obesity, defined as body mass index greater than 40 kg/m^2 measured within 4 weeks before the ATI.
7. Chronic kidney disease, defined as eGFR <60 $mL/min/1.73m^2$ on the two most proximal tests run by the study prior to the ATI.
    a. For individuals on HIV regimens that falsely lower the eGFR (for example, regimens containing dolutegravir), a minimum eGFR of 50 $mL/min/1.73m^2$ will be allowable.
8. Other medical comorbidities deemed by the Principal Investigator to confer unacceptably elevated risk at the time of the ATI.

Participant Consent, Counseling, and Education

The informed consent form has been updated to include counseling regarding the risks of COVID-19. This consent language was developed in conjunction with the CAB and has been published in a peer-reviewed journal (see Peluso et al Clinical Infectious Diseases 2020).

Prior to enrollment, participants will be counseled and provided study handouts on these topics:

- Potential risks related to COVID-19 in people with HIV
- How the study is working to mitigate these risks

Minimizing Risk of Exposure

The primary risk of exposure may be from travel to the study site. This risk will be mitigated as follows:

- Parking validation will be provided to promote self-driving for individuals with cars.
- Taxi vouchers and rideshare subsidies within San Francisco will be provided for participants who would otherwise take public transportation.
- Masks will be provided at the request of study participants.

All participants in the study will be required to observe up-to-date medical center policies regarding:

- Masking while in the research center.
- Physical distancing while in the research center.

- Pre-visit telephone health screening, which may include questions about symptoms of SARS-CoV-2 infection and documentation of the necessity of the research visit.
- In-person health screening, which may include but may not be limited to temperature and symptom checks, may be required to gain access to the research center.
- Any other medical center policies that are put in place during the study.

Where possible, we will arrange for study visits that do not require in-person interaction to take place via established telehealth procedures at San Francisco General Hospital. This may include:

- When possible, follow up visits not requiring IP administration are permitted to take place via phone or Zoom. Failure to collect vital signs or blood at such visits would not be considered a violation of the Protocol.
- When possible, arranging for laboratory monitoring during the ATI to take place closer to the participant's location. In such cases, failure to store specimens or collect vital signs at these time points would not be considered a violation of the Protocol.

SARS-CoV-2 Testing during the Study

Nucleic acid amplification tests for SARS-CoV-2 will be offered to all study participants at the following time points (test performed within 7 business days prior to the timepoint):

- Week 24, prior to lefitolimod initiation. As of Protocol Version 2.2, no further lefitolimod will be administered.
- Week 34, prior to or at the time of treatment interruption
- Every 4-6 weeks during the treatment interruption

Participants have the option to decline testing, or to obtain a test outside of the study center if this is more convenient. The suitability of the test will be at the PI's discretion. Asymptomatic participants can request more frequent testing during the ATI, but testing will not be performed on asymptomatic individuals more frequently than once every 2 weeks.

Antibody testing for SARS-CoV-2 may be performed on blood samples from various study time points, either at the time of collection or retrospectively.

Symptomatic Participants

Participants who report symptoms potentially attributable to COVID will receive assistance in navigation to a testing center through their primary care provider or the San Francisco Department of Public Health. The potential attribution of the symptoms will be at the discretion of the Principal Investigator or designee.

Study visits will be deferred at the discretion of the Principal Investigator until negative testing is confirmed or symptoms resolve. Up-to-date medical center policies regarding the timing of visits following development of symptoms will be followed.

Positive SARS-CoV-2 Nucleic Acid Amplification Test Results

In the event that a participant tests positive for SARS-CoV-2 by nucleic acid amplification testing, the response will depend upon the stage of the Protocol in which the positive test occurs:

- During the intervention portion of the study (Weeks 0-34), upcoming interventions will be delayed until deemed safe by the Principal Investigator. In general, this will be for a period of 10-14 days, according to up-to-date guidance at the time.
- During the treatment interruption, the Principal Investigator will consult with the participant regarding ongoing participation in the treatment interruption or re-initiation of antiretroviral therapy. Study continuation will be based upon up-to-date guidance at the time and will consider factors such as (1) presence or absence of symptoms in the participant, (2) current knowledge about SARS-CoV-2 infection in people with HIV, (3) current CDC and/or WHO guidance.

Re-initiation of Shelter-in-Place

In the event that shelter-in-place protocols are re-initiated in San Francisco, a meeting of the SMC will be convened within 5 business days to determine:

- Whether enrollment should be paused.
- For participants undergoing the therapeutic interventions, whether experimental therapies should be paused or delayed.
- For participants in the treatment interruption phase, whether ART should be resumed.
- For all participants, whether safety blood draws can be deferred.

COVID-19 Vaccination

The approach to COVID-19 vaccination will be determined by the phase of the trial that the participant is in when offered the vaccine. Ultimately, the final decision about the timing of the COVID-19 vaccine and the adjustments to the study schedule should be a shared decision between the participant and Principal Investigator. Guidance is below, with the understanding that not all scenarios can be anticipated and ultimately the decision on how to adjust the study schedule is at the discretion of the PI.

Study Period	Phase	Approach
Weeks 0 – 12	HIV DNA prime/boost	OK to receive COVID-19 vaccine. Study interventions should be spaced by at least one week from any individual COVID-19 vaccine.
Weeks 12 – 20	N/A	OK to receive COVID-19 vaccine. If vaccinated during this period, Week 20 visit may need to be postponed until 2 weeks after COVID-19 vaccine series completed.

Weeks 20 – 22	MVA boost and immunology endpoint	This period should take place either before a vaccine series starts or after a vaccine series is complete. Participants should **not** receive a COVID-19 vaccine during this period.
Weeks 22 – 24	N/A	OK to receive COVID-19 vaccine. If vaccinated during this period, Week 24 visit may need to be postponed until 2 weeks after COVID-19 vaccine series completed.
Weeks 24 – 33	bNAbs + TLR9 agonist	Participants who intend to be vaccinated should not enter this phase until 2 weeks elapsed since final vaccine in series.<br><br>Once participant receives the Week 24 bNAbs, the period in which to receive the lefitolimod is fixed and cannot be extended. If decision is made to be vaccinated during this phase, lefitolimod will be paused for at least 1 week before COVID-19 vaccine (if possible), and should remain paused until 2 weeks since the final vaccine in series has elapsed.<br><br>Note: This could result in the participant missing up to 9 doses of lefitolimod.<br><br>Note: As of Protocol Version 2.2, no further lefitolimod will be administered
Weeks 34-60	bNAbs + ATI	Participants who intend to be vaccinated should not enter this phase unless vaccinated.<br><br>If decision is made to be vaccinated during this phase before the participant is viremic, participant should resume ART as soon as HIV viral load is above the limit of quantification (no extended ATI).<br><br>If the participant's HIV viral load is already above the limit of quantification when decision is made to be vaccinated, participant should resume ART immediately.

23 APPENDIX D: CONSIDERATIONS RELATED TO REPRODUCTIVE HEALTH

As stated in the protocol, study volunteers cannot become pregnant or father a biological child through natural conception during the course of the study. This may place undue burden on some participants who are considering having a child and would not be able to do so for up to two years while they participate in the study. For this reason, the study team has had extensive discussions with the Institutional Review Board regarding support for participants who may wish to preserve options for alternative conception methods during the study period. For participants who meet the following criteria, the study will offer up to $500 in financial support to subsidize out-of-pocket fertility services, defined as semen or oocyte cryopreservation. To meet these criteria, participants must:

1) Meet all protocol eligibility criteria
2) Notify the study team of the intention to utilize semen or oocyte cryopreservation services prior to entry into the study (Day 0)
3) Complete semen or oocyte (egg) collection(s) prior to receiving the first dose of study medication.
4) Provide documentation of payment for use of such services within 1 year prior to entry into the study (Day 0) and/or semen or oocyte storage costs incurred during their participation in the study. Costs must be for the participant's fertility services. The ultimate decision of whether to offer this support is at the discretion of the PI.

24 APPENDIX E: UPDATES RELATED TO LIVER FUNCTION TEST MONITORING

The Safety Monitoring Committee met in August and September 2021 in response to a Grade 4 elevation in ALT that occurred in one study participant. In response to their recommendations, we have made the following changes to the Protocol.

- No additional lefitolimod will be administered to any participant.
- The 5 participants who are due to enter Stage 5 of the study (second dose of bNAbs and analytic treatment interruption (ATI) at Week 34) will have LFTs checked within 2 weeks prior to their Week 34 visit. If ALT, AST, and total bilirubin meet criteria for grade 1 or higher (AST or ALT. 1.25 x upper limit of normal (ULN), total bilirubin 1.1 x ULN), LFTs will be repeated approximately every 1-2 weeks. The team will only proceed with the second bNAb dose and ATI if/once AST, ALT, and total bilirubin are below the grade 1 threshold. LFTs will also be checked on the day of the bNAb visit, prior to bNAb administration, to obtain a pre-bNAb/ATI baseline measure.
- Participants will be staggered for Stage 5 (bNAbs/ATI) entry. One participant will enter Stage 5 initially, with LFTs checked at weeks 1 and 2 following bNAbs/ATI. If these LFTs remain below the grade 1 threshold, two more participants will enter Stage 5 and have LFTs checked 1 and 2 weeks after bNAbs/ATI. If these LFTs remain below the grade 1 threshold, the remaining 2 participants will enter Stage 5.
- In all participants, the LFT monitoring frequency will be increased to every week for four weeks, then every 2 weeks from Week 38 to Week 42, followed by monthly monitoring. If any grade 1 or higher LFT elevation is detected, the LFTs will be repeated to confirm resolution (return to below grade 1 threshold) before additional participants enter Stage 5.
- If any grade 2 or higher LFT elevation is detected, further entry into Stage 5 will be halted and a review conducted by the SMC.
- For the one participant who has not received lefitolimod, the participant may receive MVA, Week 24 bNAbs (without lefitolimod), and Week 34 bNAbs/ATI if they agree.
- A study physician will perform a targeted hepatobiliary review of symptoms and exam in all participants on the day of and prior to administration of bNAbs.
- The study team will review all concomitant medications and counsel participants to discontinue any potentially hepatotoxic agents for the duration of the study.
- The ICF will be amended to reflect the updated monitoring schedule and liver-related event.

25 APPENDIX F: PROTOCOL UPDATES

25.1 Version 1.1 – June 13, 2019

1. Changed screening period from 30 to 60 days
2. Updated Section 6 with information about the definition of early treatment initiation using EID, EDDI, and Fiebig stage
3. Updated inclusion criteria to include age <70 at time of enrollment for those initiating therapy during early infection
4. Updated exclusion criteria with formal definition of reproductive potential
5. Updated exclusion criteria with requirement for CD4 nadir <350 to be confirmed on repeat testing
6. Updated Section 10.3 to include baseline and week 47 ophthalmologic exam
7. Updated Section 11.5 to reflect 15 days for report form submission to collaborators
8. Updated Section 11.5 to reflect PSRT with PI, co-I, SMC chair, and IAVI representative
9. Updated Section 16.10 to reflect more conservative reporting to SMC
10. Added updated ICF

25.2 Version 1.4 – September 3, 2019

1. Fixed page numbering
2. Section 11.9 – updated to include closer monitoring of HIV parameters during ATI
3. Updated inclusion criteria to include age less than or equal to 67 at the time of enrollment for those beginning therapy during early infection (it will remain 65 for chronic infection)
4. Section 15.4 – revised "*week 24 post-treatment interruption" to "for 24 weeks"*
5. *Revised exclusion criterion #24 to include:* implanted electronic medical devices (e.g., cochlear implant, pacemaker, implantable cardioverter defibrillator); ii) sinus bradycardia (defined as < 50 beats per minute on exam); or iii) history of cardiac arrhythmia (e.g., supraventricular tachycardia, atrial fibrillation, or frequent ectopy). Please note, sinus arrhythmia is not excluded.
6. Section 15.5 – We have removed the reference to historical controls in the power calculation.
7. Updated the ICF with DAIDS recommendations.
8. Updated Section16.5 Data Management Procedures with updated EU directive
9. Updated SMC review with change from every 6 months to every 3 months.
10. Updated section 2.1 with reasons why we believe this regimen will be efficacious
11. Updated exclusion criteria for contraception to make wording clear that individuals of reproductive potential who will not use two forms of contraception will be excluded
12. Updated exclusion criteria items 26 (egg allergy) and 27 (cardiac adverse events). Added ECG abnormalities as listed in MVA IB.
13. Changed Section 7 to indicate individuals should remain on entry ART regimen through week 34 (not 48) of the study
14. Updated SOE to reflect checking of bNAb levels every other week following week 34
15. Edited section 10.9 to indicate that we will monitor individuals every 2 weeks during the extended ATI.

16. Updated 16.5 to include bullet point list of REDCap procedures.
17. Updated SOE to reflect viral load and CD4 checks every 2 weeks during the extended ATI (rather than every 4 weeks as previously listed)

25.3 Version 1.5 – October 25, 2019

1. Corrected TDS-IM Device to indicate version 1.0 rather than version 2.0 throughout the protocol.
2. Replaced text of section 1.6 and 8.4 with information from TDS-IM v1.0 Investigator's Brochure.
3. Added exclusion criterion #28: Individuals in whom a skin-fold measurement of the cutaneous and subcutaneous tissue for eligible injection sites exceeds 40 mm.

25.4 Version 1.6 – November 4, 2019

1. Section 1.3 - changed specific sites of injection to "injection site #1" and "injection site #2"
2. Section 1.3 - removed MVA specification of deltoid muscle
3. Section 6.3 – changed exclusion criterion #23 to read "individuals in whom the ability to observe possible local reactions at the eligible injection sites is, in the opinion of the investigator, unacceptably obscured due to a physical condition or permanent body art, or who have keloids or hypertrophic scars located within 2 cm of intended treatment site."
4. Section 6.4 – Added exclusion criteria 29-31.

25.5 Version 1.7 – November 6, 2019

1. Section 10.3 – Changed B1 visit from -2 weeks to -3 days
2. Table 8.2 – Updated to reflect "injection site #1" and "injection site #2" rather than deltoid and thigh muscles specifically

25.6 Version 1.8 – November 26, 2019

1. Protocol synopsis – updated rationale for target population with "2022" instead of "2020", clarified definition for chronic infection (6 months or more after estimated infection date), synchronized inclusion criteria with main protocol, synchronized exclusion criteria with main protocol
2. Section 6.2 – Further specified inclusion criterion 4 to make clear documentation of HIV infection
3. Section 6.3 – Synchronized exclusion criteria with protocol synopsis to indicate any prior HIV-associated malignancy, significant or progressive ophthalmologic disease

4. Section 9 – updated study procedures with precise Red Cross guidelines, updated monitoring for adverse reactions to specify monitoring for AT LEAST 4 hours after bNABs (previously just "4 hours")
5. Section 9.4 – updated with timing window for eye exams, and specifies that follow up eye exam is not necessary if a participant does not receive the bNAbs (the agent that requires monitoring for ocular toxicity)
6. Section 10.1 – updated with greater specificity regarding blood volumes and timing of monitoring
7. Section 10.3 – clarified that eye exam can occur anytime between screen and study entry (does not need to be at the same time as the B1 visit)
8. Section 10.8 – clarified that results of plasma HIV RNA testing are expected within 3 business days (previously "72 hours") to reflect reporting by our laboratory
9. Section 10.11 – clarified that additional labs can be sent at discontinuation based on the discretion of the PI/Co-I.
10. Section 13.3 – clarified that participants withdrawing following study entry will not be replaced. Participants who withdraw prior to study entry (at which point they receive the first IP administration) may be replaced.
11. Section 16.5 – Indicates that the study team will review and verify study data, additional specifications about handling study records
12. Section 17 – Updated to reflect confidentiality practices as outlined in DAIDS response letter
13. Section 17.3 – specified that participants may decline to receive a physical copy of the consent form and that their preference will be documented
14. SOE – Added an "Early D/C" column

25.7 Version 1.9 – January 15, 2020

1. Updated Protocol Synopsis to specify that participants will be followed for up to 112 weeks (rather than "two years")
2. Updated schedule of events as followed:
    a. PaxGene RNA will now be sent at B2D0 instead of screen.
    b. Added PaxGene RNA draws at W1 and W20
    c. Week 22 visit no longer optional
    d. Remove bNAb level measurement at W24
    e. Add PBMC collection at W46, W 50, W54, W68, W62, W70, W78
    f. Modify cryopreservation plasma to W48, W50, W52, W54, W56, W58, W62, W70, W78
    g. Modify serum storage to W58
    h. Modified frequency of T cell testing to every 2 weeks to reflect Section 10.8 and DAIDS review

i. Added additional pregnancy testing timepoints through end of study
j. Clarified that once ART is resumed, subjects will be followed at the time of ART restart, 4 weeks after restart, 8 weeks after restart, and 24 weeks after restart.
k. Clarified that eye exam at early discontinuation visit is required only if the initial dose of bNAbs has been received and may be completed within 16 weeks of the bNAbs dose. This replaces the eye exam scheduled between Weeks 46-50. Only one follow-up eye exam is required assuming no other triggers for eye exam are warranted after Week 24.Added additional section outlining plans for collections post-ART initiation.
l. Clarified that Δ indicates the End Study visit will be 52 weeks after the last product administration. For participants who receive the final investigational product at Week 34, the End Study visit is Week 86. If participants discontinue treatment early, the End Study visit will be 52 weeks after the last IP is received. The window for the End Study visit will be +/- 2 weeks. The End Study visit does not equate to the final visit of the study.

3. Exclusion criterion: Individuals who have previously measured phenotypic susceptibility to 10-1074 and VRC-07 within 18 months prior to screen do not need to have this test repeated, although it may be repeated at the discretion of the PI.
4. Section 9.4: Clarified that for individuals who discontinue prematurely, the ophthalmologic exam will be conducted within 16 weeks the last bNAb dose.
5. Section 9.5: Updated CBC text to reflect SOE. CBCs will be drawn at the first baseline visit (B1), entry, and all post-entry visits through week 24. CBCs will be obtained every two weeks during W24-34, and then every four weeks through the end of the study.
6. Section 9.5: Note that total and direct bilirubin will be measured at screen and at all chemistry timepoints from Week 24 onwards.
7. Section 9.12: Specified that "end of the trial" means the End Study visit.
8. Section 9.13: Specified that "end of the trial" means the End Study visit.
9. Section 10.3: Specified that individuals with prior documentation Hepatitis B immunity will not require Hepatitis B testing. Individuals with prior documentation of negative Hepatitis C antibody and who lack new risk factors will not require retesting of Hepatitis C antibody although it may be repeated at the discretion of the PI.
10. Section 10.10: Specified that "subjects who receive study products through week 34 will be followed through week 86."
11. Section 10.11: Specified that participants who discontinue prematurely will be have an end of study visit scheduled 52 weeks after the last dose of study product administration. This will include routine safety studies, CD4+/CD8+ T cell counts, plasma and PBMCs cryopreservation, and any other specimen collection deemed pertinent to safety or data integrity as assessed by PI/Co-I.
12. Section 10.3: Individuals who have previously measured phenotypic susceptibility to 10-1074 and VRC-07 within 6 months prior to screen do not need to have this test repeated, although it may be repeated at the discretion of the PI.
13. Section 10.4: Updated to reflect PaxGene collection as indicated in SOE

14. Section 10.6: Week 22 visit is no longer optional.
15. Section 10.7: Participants will be asked about the development of new ophthalmologic symptoms at every visit. Routine safety monitoring studies will be performed at weeks 24, 26, 28, 30, and 32. Indicated plasma HIV RNA also to be measured at W30.
16. Section 10.8: Updated to reflect serum collection at W58 instead of W60.
17. Section 10.8: Participants will be asked about the development of new ophthalmologic symptoms at every visit.
18. Section 10.8: Indicated that eye exam will occur between W46 and W50. Participants will be asked about the development of new ophthalmologic symptoms at every visit and referred to an ophthalmologist to evaluate new symptoms of any grade.
19. Section 10.8: Specified that the last plasma HIV RNA level should be below the limit of quantification on the assay used (rather than specifically <40 copies/mL) to allow for variability between assays. A PAX Gene specimen will be collected at W42 and at the end of the study.
20. Section 10.8: Updated VL result window from 3 to 4 business days based upon reported turnaround time from lab.
21. Corrected title of Section 10.9 to "Stage 6."
22. Section 10.9: Once ART is reinitiated, subjects will be evaluated at Weeks 4 and 8 on ART. If viral load is not below the level of quantification by Week 8, participants will be evaluated every four weeks until the viral load is confirmed to less that the level of quantification. A follow up visit will occur 24 weeks after resumption of ART.
23. Routine safety studies, CD4+/CD8+ T cell counts and viral load will be performed at Weeks 4, 8, 24 and at any visits required between Week 8 and Week 24 to evaluate decrease of viral load to below level of quantification. Plasma and PBMCs will be collected and cryopreserved at the time of ART resumption and at Weeks 4, 8 and 24 on ART.
24. Section 11: Participants will also be asked to complete a patient diary to collect local injection site reactions and systemic reactogenicity events for seven days following each study product administration. These include but are not limited to local injection site reactions and systemic events including fever, chills, headache, fatigue or malaise, myalgias, arthralgias, nausea, and vomiting.
25. Section 15.3: With regard to Stage 4 (bNAbs and lefitolimod), we will include analysis of ophthalmologic adverse events and elevations in direct bilirubin, as well as episodes compatible with cytokine release following bNAb administration.
26. Section 16.10: Added two pausing criteria per FDA request
    a. Any ophthalmologic adverse event of any severity grade, pending examination by an ophthalmologist and review by the SMC.
    b. Any abnormal elevation in direct bilirubin pending evaluation by a hepatologist.
    c. One or more participants as experienced an SAE of any grade.
27. Section 16.10: Clarified that in the event that a pause is triggered, both enrollment and administration of study products will pause until review by the SMC has been completed.

28. Consent form Section 19.6: Removed text about certificate of confidentiality per discussion with IRB.

25.8 Version 2.0 – October 6, 2020

1. Protocol Synopsis:
    a. Corrected numbers in inclusion/exclusion criteria
    b. Changed inclusion criterion 3 to age less than or equal to 65 at the time of enrollment.
    c. Specified that individuals must be on a regimen that does not include an NNRTI within 4 weeks of the first study product administration.
    d. Specified that individuals need to have continuous suppression for 12 months, not 24 months.
    e. Removed "indirect bilirubin < ULN" from inclusion criteria. This is a calculated value without a normal range in the ZSFG clinical laboratory.
    f. Clarified that additional information on interpretation of Monogram assays is available in the relevant protocol section.
    g. Changed "exposure to any vaccination within 30 days of study enrollment" to "within 7 days of study enrollment" (exclusion criterion 16)
2. Section 6.2:
    a. Updated inclusion criterion 3 to 65 years at time of enrollment.
    b. Updated inclusion criterion 5 to specify that individuals must not be on an NNRTI-based regimen within 4 weeks of first study product administration.
    c. Updated inclusion criterion 7 to indicate 12 months of viral suppression is needed.
3. Section 6.3:
    a. Updated first criterion to indicate subjects receiving an NNRTI will be excluded if unable to switch to a different regimen.
    b. Updated criteria with regard to Monogram assay for bNAb susceptibility, clarifying eligibility criteria.
    c. Changed "exposure to any vaccination within 30 days of study enrollment" to "within 7 days of study enrollment" (exclusion criterion 16)
4. Section 8.1: Removed line about ongoing IAVI product management.
5. Section 9.11:
    a. Updated criteria with regard to Monogram assay for bNAb susceptibility, clarifying eligibility criteria.
    b. Updated timing of innate immune cell phenotypes
6. Section 9.12: Updating timing of T cell immunogenicity assays
7. Section 9.13: Updated timing of transcriptomic measurements.
8. Section 9.15: Updated timing of virologic assays
9. Section 10.3:
    a. Updated screening section to include updated language regarding Monogram assay.

   b. Clarified that serum may be collected at B1. Serum must be collected at B2D0 or within 4 weeks prior. It could occur at the B1 visit, an interim visit, or at the B2D0 visit.
10. Section 10.4: Clarified that the Week 1 visit will take place 7 +/- 2 days from the date of the B2D0 visit.
11. Section 10.6: Removed PAXGene from W20.
12. Section 10.7: Serum will be collected and stored at week 24.
13. Section 10.8:
    a. Added PAXGene to W38 and W52
    b. Removed PAXGene from "End" of study
    c. Serum will be collected and stored at week 34.
    d. Specified that when laboratory testing is performed remotely, storage of specimens may be deferred.
14. Section 11.2: Corrected broken link to Table 1.
15. Section 11.5: Corrected references to IAVI to clarify that a representative may be on the SMC.
16. Section 16.9:
    a. Relabeled "Regulatory and Data Monitoring"
    b. Removed IAVI, who are unable to conduct on-site monitoring visits, from this section.
    c. Specified that an internal monitoring team will conduct all study monitoring related to Regulatory and Data – note this does NOT affect safety monitoring.
17. Section 16.10: Specified that elevated bilirubin should be confirmed on repeat testing.
18. Schedule of Events:
    a. Indicated serum collection at week 24 and 34
    b. Added note to indicate the specimen storage may be deferred in cases where visits occur remotely.
19. Consent Form
    a. Clarified duration of W24 visit
    b. Updated total blood volume to 3300mL
    c. Updated payments for IV infusions to $300
    d. Added peer-reviewed consent language related to COVID-19
20. Appendices
    a. Added appendix B: Risk mitigation
    b. Added appendix C: COVID-19
    c. Relabeled Protocol Updates as appendix D

25.9 Version 2.1 – March 6, 2021

1. Synopsis
   a. Study Design
      i. Changed 10 to 9 doses of lefitolimod

ii. Updated to indicate lefitolimod starts at Week 25

2. Section 1.3
    a. Updated to indicate 9 weekly doses of lefitolimod
3. Section 2.3
    a. Updated figure to indicate lefitolimod starts at Week 25
4. Section 4.1
    a. Updated to indicate lefitolimod starts at Week 25
5. Section 9.5
    a. Update section to remove lefitolimod
6. Section 9.12 Update weekly doses of lefitolimod from 10 to 9
7. Section 10.7
    a. Updated to indicate lefitolimod starts at Week 25, defined as 1 week (+/- 3 days) and that following the initial dose, weekly lefitolimod doses may be administered on any day of the week provided the doses are at least 5 days apart.
    b. Update number of lefitolimod doses
8. Section 10.12
    a. Updated to indicate lefitolimod starts at Week 25
    b. Updated Week 25 window following bNAbs from 7 days +/-1 day to 7 days +/-3 days
    c. Updated weekly lefitolimod doses following the initial dose may be administered on any day of the week provided doses are at least 5 days apart.
9. Section 16.10
    a. Updated information about study pause related to ophthalmologic AEs to reflect that other IP administrations can continue but bNAbs must be held until SMC review
10. Appendix C
    a. Added COVID-19 vaccine considerations
11. Appendix D
    a. Renamed prior Appendix D to Appendix E
    b. Appendix D addresses reproductive health concerns

25.10 Version 2.2 – Sept 21, 2021

Note: All updates in this version are related to severe liver adverse events that are possibly related to lefitolimod and/or the broadly neutralizing antibodies.

1. Added Appendix E – protocol updates related to recent Liver Function Testing abnormalities and revised consent form.
2. The following sections were updated to indicate that no further lefitolimod will be administered
    a. 1.3
    b. 2.3
    c. 4.1
    d. 8.8

    e. 9.5
    f. 10.7
    g. 10.12
    h. 15.3
    i. Appendix C
3. Section 10.8 – Added information about Staggered Entry into Week 34
4. Section 10.8 – Added information about LFT monitoring on day of bNAb visit, as well as weekly LFT monitoring during week 34-38 and every other week monitoring for Week 38 to 42.
5. Section 10.12 – Added updated LFT monitoring
6. Section 16.10 – Added pause criteria for grade 2 or greater LFT elevations in new Stage 5 entrants
7. Section 9.5-- Added note that no more lefitolimod will be administered as of this Protocol Version
8. Section 9.12—Added wording to note that lefitolimod will not be administered as of this protocol which may lead to variation in time points for cellular measurements.
9. 10.12 Added wording to note that since lefitolimod was withheld for some participants the number of weeks between the End of Study Visit and the last lefitolimod date will not be consistent for all participants.
