# Supplementary material for: Correlates of HIV-1 control after combination immunotherapy
Source: Nature. 2025 Dec 1;650(8100):187–95. doi: 10.1038/s41586-025-09929-5 (PMC12872443; doi:10.1038/s41586-025-09929-5)
Supplement: Supplementary file 2 — Reporting Summary [file 41586_2025_9929_MOESM2_ESM.pdf]

nature portfolio

Corresponding author(s): Steven Deeks, Rachel Rutishauser

Last updated by author(s): 10/22/2025

Reporting Summary

Nature Portfolio wishes to improve the reproducibility of the work that we publish. This form provides structure for consistency and transparency in reporting. For further information on Nature Portfolio policies, see our Editorial Policies and the Editorial Policy Checklist.

Statistics

For all statistical analyses, confirm that the following items are present in the figure legend, table legend, main text, or Methods section.

n/a	Confirmed	
☐	☒	The exact sample size (*n*) for each experimental group/condition, given as a discrete number and unit of measurement
☐	☒	A statement on whether measurements were taken from distinct samples or whether the same sample was measured repeatedly
☐	☒	The statistical test(s) used AND whether they are one- or two-sided<br>*Only common tests should be described solely by name; describe more complex techniques in the Methods section.*
☒	☐	A description of all covariates tested
☒	☐	A description of any assumptions or corrections, such as tests of normality and adjustment for multiple comparisons
☐	☒	A full description of the statistical parameters including central tendency (e.g. means) or other basic estimates (e.g. regression coefficient) AND variation (e.g. standard deviation) or associated estimates of uncertainty (e.g. confidence intervals)
☐	☒	For null hypothesis testing, the test statistic (e.g. *F*, *t*, *r*) with confidence intervals, effect sizes, degrees of freedom and *P* value noted<br>*Give P values as exact values whenever suitable.*
☒	☐	For Bayesian analysis, information on the choice of priors and Markov chain Monte Carlo settings
☒	☐	For hierarchical and complex designs, identification of the appropriate level for tests and full reporting of outcomes
☐	☒	Estimates of effect sizes (e.g. Cohen's *d*, Pearson's *r*), indicating how they were calculated

Our web collection on statistics for biologists contains articles on many of the points above.

Software and code

Policy information about availability of computer code

Data collection	No software used for data collection.
Data analysis	SpectroFlo (v3.0.3 or v3.2.1), CellEngine (no version numbers available), FlowJo (v10), R (v4.3 or 4.4.2), GraphPad Prism (v10), cyCombine (0.2.19), Catalyst (1.28.0), ggplot2 (v4.0), ComplexHeatMap (v2.18), Monolix software (Lixoft v2023R1), Trim Galore (v0.6), FastQC (v0.11.2), Bowtie2 (v2.4.2), STAR (v2.7.10b), lmerTest (v3.1), Premessa (0.3.4). Bulk RNAseq analysis code: https://github.com/galelab/Rutishauser_post_intervention_HIV_control

For manuscripts utilizing custom algorithms or software that are central to the research but not yet described in published literature, software must be made available to editors and reviewers. We strongly encourage code deposition in a community repository (e.g. GitHub). See the Nature Portfolio guidelines for submitting code & software for further information.

Data

Policy information about availability of data

All manuscripts must include a data availability statement. This statement should provide the following information, where applicable:

- Accession codes, unique identifiers, or web links for publicly available datasets
- A description of any restrictions on data availability
- For clinical datasets or third party data, please ensure that the statement adheres to our policy

Bulk RNAseq data available here: GSE288962. The human genome used for RNAseq alignment (GRCh38, Ensembl) is available here: https://www.ncbi.nlm.nih.gov/

datasets/genome/GCF_000001405.26/. Full processed (gated and/or clustered) mass cytometry and flow cytometry data are available in Supplementary Table 4. De-identified raw fcs files with mass cytometry data were deposited on Mendeley: doi: 10.17632/3wfk6rrht.1. Raw flow cytometry fcs files will be made available upon request.

Research involving human participants, their data, or biological material

Policy information about studies with human participants or human data. See also policy information about sex, gender (identity/presentation), and sexual orientation and race, ethnicity and racism.

Reporting on sex and gender	This study enrolled 9 cisgender men and 1 transgender woman; sex and gender was determined based on self-reporting. Sub-analyses based on gender were not performed given the small size of the sample.
Reporting on race, ethnicity, or other socially relevant groupings	We report on race and ethnicity using standard terminology required in NIH reporting. Race and ethnicity was determined based upon participant self-report. Sub-analyses based on race/ethnicity were not performed given the small size of the sample.
Population characteristics	All participants were people living with HIV. For the interventional clinical trial: The median age of participants in the study was 36 years (range 32-55); the trial excluded children and adults >age 65 years. 7 participants had initiated ART during the early phase of HIV infection and 3 participants had initiated ART during the chronic phase of HIV infection. For the prospective ATI trial: The median age of participants in the study was 60 years (range 32-75); the trial excluded children. 7 participants were controllers prior to ART initiation, 13 were prior non-controllers.
Recruitment	For both clinical studies: Participants were recruited from an existing observational cohort of adults living with HIV in the San Francisco Bay Area (SCOPE). Participants in SCOPE who had expressed interest in hearing about clinical trials were informed about the existence of this clinical trial in the context of a SCOPE visit. They were given the opportunity to hear more about the study and, if interest, schedule a dedicated screening visit. Participants who opted in were likely to be highly engaged in HIV research and able to commit to the intensive study schedule; as the trial was conducted during the COVID-19 pandemic they were also likely to be willing to tolerate the risk of travel to the research center and interaction with study staff during the pandemic. We do not anticipate that these potential biases would impact the biological results of the studies.
Ethics oversight	UCSF IRB

Note that full information on the approval of the study protocol must also be provided in the manuscript.

Field-specific reporting

Please select the one below that is the best fit for your research. If you are not sure, read the appropriate sections before making your selection.

☒ Life sciences ☐ Behavioural & social sciences ☐ Ecological, evolutionary & environmental sciences

For a reference copy of the document with all sections, see nature.com/documents/nr-reporting-summary-flat.pdf

Life sciences study design

All studies must disclose on these points even when the disclosure is negative.

Sample size	No sample size was pre-specified for the analysis included here. This was designed as a proof-of-concept mechanistic study.
Data exclusions	1. For analyses of immune responses related to rebound, the participant with no rebound was excluded.<br>2. For analyses including the early "postR1" timepoint, one participant who had experienced rebound was excluded due to no early post-rebound PBMC sample available comparable to the timing (<28 days) and viral load (<2,600 copies/mL) of the other participants in the study.
Replication	The clinical and immunologic results of this study cannot be replicated because there is not another clinical trial with the same design, however we have highlighted several studies with similar approaches are now underway or planned and, in some cases, designed to build upon the work that was done in this study.
Randomization	This was a single-arm study.
Blinding	Laboratory researchers were blinded to outcomes during initial analyses.

Reporting for specific materials, systems and methods

We require information from authors about some types of materials, experimental systems and methods used in many studies. Here, indicate whether each material, system or method listed is relevant to your study. If you are not sure if a list item applies to your research, read the appropriate section before selecting a response.

Materials & experimental systems

n/a	Involved in the study
☐	☒ Antibodies
☒	☐ Eukaryotic cell lines
☒	☐ Palaeontology and archaeology
☒	☐ Animals and other organisms
☐	☒ Clinical data
☒	☐ Dual use research of concern
☒	☐ Plants

Methods

n/a	Involved in the study
☒	☐ ChIP-seq
☐	☒ Flow cytometry
☒	☐ MRI-based neuroimaging

Antibodies

Antibodies used	All antibodies used are commercially available and extensively used. All antibodies and their clone names are listed in Supplemental Table 7. Flow cytometry antibodies: CD45RA (BUV395, clone 5H9, BD, Cat# 740315), Zombie UV viability dye (BioLegend, Cat# 423107), CX3CR1 (BUV563, clone 2A9-1, BD, Cat# 749357), CD27 (BUV805, clone M-T271, BD, Cat# 742012), CD14 (BV510, clone M5E2, BioLegend, Cat# 301842), CD19 (BV510, clone HIB19, BioLegend, Cat# 302242), TCRγδ (BV510, clone B1, BioLegend, Cat# 331220), CD8 (BV570, clone RPA-T8, BioLegend, Cat# 301037), CD39 (BV605, clone A1, BioLegend, Cat# 328236), CD103 (BV650, clone Ber-ACT8, BD, Cat# 743653), CXCR5 (BV750, clone RF8B2, BD, Cat# 747111), PD-1 (BV785, clone EH12.2H7, BioLegend, Cat# 329929), TIGIT (PerCP-eFluor710, clone MBSA43, Thermo Fisher, Cat# 46-9500-42), CXCR3 (PE-CF594, clone 1C6, BD, Cat# 562451), CD95 (PE-Cy5, clone DX2, BioLegend, Cat# 305610), CCR7 (PE-Cy7, clone G043H7, BioLegend, Cat# 353226), CD4 (SparkNIR685, clone SK3, BioLegend, Cat# 344657), CD127 (APC-f750, clone A019D5, BioLegend, Cat# 351349), MIP1β (BV421, clone D21-1351, BD, Cat# 562900), IFNγ (eF450, clone 4S.B3, Thermo Fisher, Cat# 48-7319-42), CD3 (BV480, clone UCHT-1, BD, Cat# 566105), IL-2 (BV711, clone MQ1-17H12, BioLegend, Cat# 500345), Perforin (PerCP-Cy5.5, clone B-D48, BioLegend, Cat# 353314), Granzyme B (PE-Cy5.5, clone GB11, Thermo Fisher, Cat# GRB18), TNFα (R718, clone Mab11, BD, Cat# 557996), T-bet (Alexa Fluor 488, clone 4B10, BioLegend, Cat# 644829), TCF1 (PE, clone 7F11A10, BioLegend, Cat# 655207), TOX (APC, Miltenyi, Cat# 130-118-335), CD107a (RY586, clone H4A3, BD, Cat# 568484), CD8 (APC-R700, clone SK1, BD, Cat# 565192), CD45RA (APC-Cy7, clone HI100, Biolegend, Cat# 304128), CD3 (BV650, clone SK7, BD, Cat# 563999), CD4 (BV711, clone OKT4, Biolegend, Cat# 317440). Mass cytometry antibodies: CD45 (clone HI30, Fluidigm, Cat# 3089003B), CD11b (clone ICRF44, BioLegend, Cat# 301302), CD3 (clone UCHT1, BioLegend, Cat# 300402), CD19 (clone H1B19, BioLegend, Cat# 302202), CD69 (clone FN50, BioLegend, Cat #310902), CD123 (clone 6H6, BioLegend, Cat# 306002), CD4 (clone RPA-T4, BioLegend, Cat# 300502), CD8 (clone RPA-T8, BioLegend, Cat# 301002), CD11c (clone Bu15, BioLegend, Cat# 337202), CD20 (clone 2H7, BioLegend, Cat# 302302), CD138 (clone DL-101, BioLegend, Cat# 352302), CD14 (clone M5E2, BioLegend, Cat# 301802), CD21 (clone Bu32, BioLegend, Cat# 313502), CXCR3 (clone G025H7, BioLegend, Cat# 353733), CD45RA (clone HI100, BioLegend, Cat# 304102), CD40 (clone 5C3, BioLegend, Cat# 334302), PD-L1 (clone 29E.2A3, BioLegend, Cat# 329702), CD68 (clone Y1/82A, BioLegend, Cat# 333802), CD27 (clone O323, BioLegend, Cat# 302802), CCR2 (clone K036C2, BioLegend, Cat# 357202), T-bet (clone 4B10, BioLegend, Cat# 644802), CD303 (clone 201A, BioLegend, Cat# 354202), CD80 (clone L307.4, BD, Cat# 557223), CD86 (clone IT2.2, BioLegend, Cat# 305401), CX3CR1 (clone 2A9-1, BioLegend, Cat# 341602), CD24 (clone MI5, BioLegend, Cat# 311102), CD141 (clone AD5-14H12, Miltenyi, Cat# 130-108-033), CD38 (clone HIT2, Fluidigm, Cat# 3167001B), Ki-67 (clone B56, Fluidigm, Cat# 3168007B), CD71 (clone CY1G4, BioLegend, Cat# 334102), IgD (clone IA6-2, BioLegend), CXCR5 (clone RF8B2, Fluidigm, Cat# 3171014B), BDCA1 (clone L161, BioLegend, Cat# 331502), IgM (clone MHM-88, BioLegend, Cat# 314502), HLA-DR (clone L243, BioLegend, Cat# 307602), PD-1 (clone EH12.2H7, BioLegend, Cat# 329902), CD56 (clone HCD56, Fluidigm, Cat# 3176008B), CD16 (clone 3G8, Fluidigm, Cat# 3209002B), Granzyme B (clone GB11, Bio-Rad, Cat# MCA2120), Granzyme A (clone CB9, BioLegend, Cat# 507202), CD95 (clone DX2, BioLegend, Cat# 305602), ICOS (clone C398.4A, BioLegend, Cat# 313539), γδ TCR (clone 11F2, Fluidigm, Cat# 3152008B), CD28 (clone CD28.2, BioLegend, Cat# 302902), CD39 (clone A1, BioLegend, Cat# 328202), Perforin (clone B-D48, Abcam, Cat# Ab7225), TCF1 (clone 7F11A10, BioLegend, Cat# 655202), CTLA-4 (clone 14D3, Fluidigm, Cat# 3161004B), FOXP3 (clone PCH101, Fluidigm, Cat# 3162011a), EOMES (clone WD1928, Thermo Fisher, Cat# 14-4877-82), CD127 (clone A019D5, BioLegend, Cat# 351302), TIGIT (clone A15153G, BioLegend, Cat# 372702), CCR7 (clone G043H7, BioLegend, Cat# 353202), Bcl-2 (clone 100, BioLegend, Cat# 658702), CD103 (clone Bcr-ACT8, BioLegend, Cat# 350202).
Validation	All antibodies used for flow cytometry and mass cytometry are commercially available and have been previously validated by the manufacturing companies and extensively used. Validation information for each antibody can be found on the vendor's website.

Clinical data

Policy information about clinical studies
All manuscripts should comply with the ICMJE guidelines for publication of clinical research and a completed CONSORT checklist must be included with all submissions.

Clinical trial registration	NCT04357821, NCT00187512, NCT04359186
Study protocol	A redacted version of the protocol has been made available.
Data collection	The clinical data and biospecimens were collected between 2020-2023 at our research center in San Francisco, California.
Outcomes	Primary safety outcome: proportion of individuals experiencing a grade 3 or greater adverse event, definitely, probably or possibly related to study treatment (see methods and extended data/supplementary tables for summary). Primary efficacy outcome: proportion of individuals achieving post-intervention control. Secondary/Other Efficacy Endpoints: cell-associated HIV DNA and RNA measures assessed via ddPCR and HIV transcription profiling; magnitude of T cell responses assessed by intracellular cytokine staining; proliferative capacity of T cell responses assessed by T cell proliferation assays; broad immunophenotyping by mass

cytometry; transcriptional signatures by bulk RNA sequencing; levels of broadly neutralizing antibodies assessed by Meso Scale Discovery-based platform; phenotypic susceptibility of HIV to bNabs assessed by PhenoSense mAb assay

Plants

Seed stocks	n/a
Novel plant genotypes	n/a
Authentication	n/a

Flow Cytometry

Plots

Confirm that:

☒ The axis labels state the marker and fluorochrome used (e.g. CD4-FITC).

☒ The axis scales are clearly visible. Include numbers along axes only for bottom left plot of group (a 'group' is an analysis of identical markers).

☒ All plots are contour plots with outliers or pseudocolor plots.

☒ A numerical value for number of cells or percentage (with statistics) is provided.

Methodology

Sample preparation	Sample preparation for flow cytometry is described in detail in the methods section.
Instrument	Cytek 5L Aurora
Software	Data were collected with SpectroFlo (v3.0.3 or v3.2.1) and analyzed with FlowJo v10
Cell population abundance	no sorting performed; Abundance of populations are indicated in the gating figures of the manuscript.
Gating strategy	described in detail in supplementary information

☒ Tick this box to confirm that a figure exemplifying the gating strategy is provided in the Supplementary Information.
