# Supplementary material for: Correlates of HIV-1 control after combination immunotherapy
Source: Nature. 2025 Dec 1;650(8100):187–95. doi: 10.1038/s41586-025-09929-5 (PMC12872443; doi:10.1038/s41586-025-09929-5)
Supplement: Supplementary file 11 — bNAb population PK model parameters. [file 41586_2025_9929_MOESM11_ESM.docx]

**Table S8:** bNAb population pharmacokinetic (PK) model parameters.

| bNAb | Parameter^2,3^ | Estimate | S.E.^1^ | %R.S.E.^1^ |
| --- | --- | --- | --- | --- |
| 10-1074 | **𝛳** _CL_ | 0.0038 | 0. 00027 | 7.17 |
|  | **𝛳** _V Central_ | 0.12 | 0.0034 | 2.84 |
|  | **Ω** _CL_ | 0.22 | 0.049 | 22.5 |
|  | **𝜎** _Proportional Error_ | 0.37 | 0.022 | 5.95 |
| VRC07-523LS | **𝛳** _CL_ | 0.002 | 0.000084 | 4.12 |
|  | **𝛳** _V Central_ | 0.1 | 0.0093 | 9.32 |
|  | **𝛳** _Q_ | 0.0013 | 0.00039 | 31.0 |
|  | **𝛳** _V Peripheral_ | 0.026 | 0.0047 | 17.9 |
|  | **Ω** _CL_ | 0.12 | 0.029 | 24.3 |
|  | **Ω** _V Central_ | 0.2 | 0.082 | 42.2 |
|  | **𝜎** _Proportional Error_ | 0.14 | 0.0083 | 5.81 |

1. S.E. is the standard error, %R.S.E. is the relative standard error
2. **𝛳**’s are the typical population estimates, **Ω**’s are the estimates for between subject variability and **𝜎**’s are the estimates for residual unexplained variability.
3. Clearance (CL) in L/day/kg, Volume of Distribution (V) in L/kg, Inter-compartmental CL (Q) in L/day/kg
